# An optimization-driven hierarchical deep learning approach using the Gray Langurs algorithm for data-driven seismic activity prediction

**DOI:** 10.1038/s41598-026-56169-2

**Published:** 2026-06-17

**Authors:** Mahmoud Shabrawy, El-Sayed M. El-Kenawy, Nahla B. Abdel-Hamid, Mohamed M. Abdelsalam

**Affiliations:** 1 Computer Engineering and Control Systems Department, Faculty of Engineering, Mansoura University, Mansoura, Egypt; 2 Department of Communications and Electronics, Delta Higher Institute of Engineering and Technology, Mansoura, 35111 Egypt; 3 Faculty of Engineering, Mansoura National University, Gamasa, 35712 Egypt

**Keywords:** Data-driven seismic activity prediction, Neural hierarchical interpolation (N-HITS), Gray Langurs optimizer (GLO), Metaheuristic hyperparameter optimization, Seismic time-series trend modeling, Engineering, Mathematics and computing, Natural hazards, Solid Earth sciences

## Abstract

The statistical prediction of seismic activity patterns from historical earthquake catalog data remains a major challenge in data-centered seismic hazard analysis because seismic time series are non-stationary, multi-scale, and clustered in nature. Existing data-driven seismic prediction pipelines often emphasize architectural innovation while giving less attention to systematic hyperparameter optimization, which is essential for achieving strong predictive performance. This work is motivated by the need for an integrated and computationally efficient data-driven time-series modeling framework. Accordingly, a hierarchical deep learning-metaheuristic optimization paradigm is proposed based on the Neural Hierarchical Interpolation for Time Series Forecasting (N-HITS) algorithm and the Gray Langurs Optimizer (GLO). We conduct a systematic benchmarking of N-HITS against state-of-the-art deep time-series prediction models trained under identical preprocessing and training conditions, followed by adaptive hyperparameter optimization. Baseline analysis showed that N-HITS, with a coefficient of determination ($R^2$) of 0.921 and a Mean Squared Error (MSE) of 0.00234, was the strongest standalone model. Following GLO-based hyperparameter optimization, performance improved to an $R^2$ of $0.9892 \pm 0.0049$ and an MSE of 7.980e-05 ± 7.980e-07, indicating substantial error reduction and higher convergence stability. These results highlight the importance of optimization intelligence in catalog-based statistical seismic activity prediction and position hierarchical deep learning with adaptive metaheuristic search as a scalable architecture for seismic trend monitoring. However, the proposed model relies only on historical seismic catalog patterns and does not incorporate tectonic processes or geophysical drivers; therefore, its outputs should be interpreted as statistical trend estimates rather than physically reliable earthquake predictions.

## Introduction

Earthquake forecasting is one of the most scientifically challenging and societally important problems in geophysical research because seismic processes are inherently nonlinear, non-stationary, and multi-scale^1–3^. Unlike many physical systems that can be modeled using deterministic frameworks with direct observability, seismic phenomena arise from intricate interactions of stress accumulation and release occurring over heterogeneous spatial and temporal domains^4,5^. The absence of direct measurements of stress fields at seismogenic depths necessitates reliance on earthquake catalogs as the primary observable proxy for tectonic evolution. Accordingly, recent advances in deep learning and computational intelligence have been increasingly employed to extract latent spatiotemporal patterns from large-scale seismic datasets.

However, forecasting performance is not determined only by the sophistication of the learning architecture. It is also strongly influenced by the quality, consistency, and structural properties of the underlying seismic data^6,7^. Earthquake catalogs are subject to multiple sources of uncertainty, including temporal inconsistencies, magnitude incompleteness, depth estimation errors, and spatial imprecision. These factors can introduce systematic biases into the data distribution, thereby affecting the reliability of learned representations. As a result, seismic forecasting must be approached as a data-centric problem, in which preprocessing, feature representation, and model design are tightly coupled components of a unified predictive framework.

A second major challenge lies in hyperparameter configuration. Modern time-series forecasting architectures, including residual-based models, recurrent neural networks, convolutional-recurrent hybrids, and transformer-based attention mechanisms, possess strong representational capabilities, but their performance depends heavily on proper tuning of parameters such as learning rate, batch size, network depth, and regularization strength^8,9^. The associated optimization problem is typically high-dimensional, non-convex, and computationally expensive, making traditional approaches such as manual tuning or grid search impractical. Furthermore, deterministic optimization strategies often suffer from premature convergence to local optima, particularly in complex model landscapes.

Metaheuristic optimization algorithms offer a practical solution to this difficulty. Inspired by natural, biological, and physical processes, these algorithms use population-based search mechanisms to balance exploration and exploitation in complex search spaces. Techniques such as Particle Swarm Optimization (PSO), Genetic Algorithms (GA), Bat Algorithm (BA), Biogeography-Based Optimization (BBO), Differential Evolution (DE), Stochastic Fractal Search (SFS), and Multiverse Optimization (MVO) have demonstrated strong capabilities in solving high-dimensional optimization problems^10,11^. Their adaptability and robustness make them particularly suitable for hyperparameter tuning in deep learning-based seismic forecasting systems^12,13^.

Motivated by these challenges, this study aims to develop an integrated and scalable seismic forecasting framework that jointly addresses data integrity, deep representation learning, and hyperparameter optimization. Such integration is essential for achieving reliable and generalizable predictive performance, especially when dealing with large-scale and heterogeneous datasets such as national earthquake catalogs. In this work, the Canadian earthquake catalog is selected as a representative case study due to its long temporal coverage and diverse tectonic settings^14,15^.

Despite the growing body of research in this field, several important gaps remain. First, existing studies often treat deep learning architectures and optimization strategies as independent components, without systematically analyzing their interaction and combined impact on forecasting performance. Second, many empirical evaluations are conducted on limited datasets or short temporal windows, which restricts the generalizability and robustness of the conclusions. Third, there is a lack of standardized benchmarking frameworks that ensure consistent preprocessing, fair comparison across models, and statistically rigorous evaluation protocols. These limitations hinder the development of a comprehensive understanding of how model architecture and optimization strategy jointly influence seismic forecasting performance.

The novelty of the proposed framework does not lie solely in combining deep learning with metaheuristic optimization, since this general direction has been explored in prior studies. Rather, its novelty lies in the unified and reproducible benchmarking design through which multiple forecasting architectures and multiple optimization algorithms are evaluated under identical preprocessing, training, and evaluation conditions. In this setting, N-HITS is not only integrated with GLO, but also systematically positioned against alternative deep learning models and competing optimizers, allowing the contribution of architecture, optimization strategy, and their interaction to be isolated more clearly. This provides a more rigorous understanding of model–optimizer synergy in seismic forecasting than studies that focus on a single architecture or a single optimization method in isolation.

To address these gaps, this study proposes a unified benchmarking framework for earthquake forecasting that integrates standardized data preprocessing, multiple deep learning architectures, and a diverse set of metaheuristic optimization algorithms within a consistent experimental setting. By evaluating both model design and optimization strategy under identical conditions, the proposed framework enables a fair and systematic comparison of their respective contributions to predictive performance.

The main contributions of this study are summarized as follows:

- We develop an integrated and reproducible earthquake forecasting framework that combines deep learning architectures with metaheuristic hyperparameter optimization, thereby bridging the gap between model design and optimization strategy.
- We conduct a comparative analysis of multiple state-of-the-art time-series forecasting models, including residual networks, recurrent architectures, convolutional-recurrent hybrids, and transformer-based models, under standardized preprocessing and training conditions.
- We introduce the Gray Langurs Optimizer (GLO) as a novel adaptive optimization strategy and benchmark it against a wide range of established metaheuristic algorithms.
- We investigate the impact of optimization strategies on forecasting accuracy, training stability, and generalization performance, highlighting the critical role of optimization intelligence in deep learning systems.
- We provide a detailed computational efficiency analysis, including training time, memory consumption, and processing requirements, to assess the feasibility of deploying optimized forecasting systems in real-world seismic monitoring applications.
- We propose a scalable and transferable evaluation pipeline that can be readily adapted to other regional and national earthquake catalogs, facilitating future research in data-driven seismic hazard assessment.

The remainder of this paper is organized as follows. Section “Related work” presents the Related Work. Section “Material and methods” describes the materials and methods, including the dataset, preprocessing pipeline, forecasting models, and optimization strategies. Section “Empirical results” presents the experimental results. Section “Discussion” discusses the findings and their implications for seismic hazard monitoring. Finally, “Limitations of the study” concludes the paper and outlines directions for future research.

## Related work

Machine learning (ML) and deep learning (DL) have been widely applied in earthquake-related research, including catalog-based forecasting, precursor analysis, ground-motion prediction, deformation monitoring, early warning, and seismic hazard assessment^16^. However, because the present study focuses on catalog-based seismic time-series forecasting and hyperparameter optimization, this section has been shortened and reorganized to emphasize studies most directly related to earthquake catalog modeling, deep temporal forecasting, benchmarking, and optimization.

Catalog-based seismic forecasting remains central in data-driven earthquake research because earthquake catalogs provide long-term observations of event occurrence time, magnitude, depth, and location. Adaptive forecasting frameworks have shown that prediction performance can be improved by optimizing temporal windows, magnitude thresholds, and regional clustering criteria^17^. Short-horizon catalog-based magnitude-category prediction has also been studied using feature engineering and ensemble learning, demonstrating the value of catalog-derived predictors for near-term seismic activity estimation^18^. Comparative studies have further shown that deep learning models, particularly LSTM-based approaches, can outperform classical statistical methods such as ARIMA and singular spectrum analysis when modeling nonlinear seismic time series^19^. Hybrid statistical models, such as SARIMAX with exogenous geological or geodetic variables, have also demonstrated that additional contextual information can improve forecasting performance when such variables are available^20^.

Deep temporal models have increasingly been adopted for earthquake magnitude and frequency forecasting. Transformer-based models have shown promising performance by capturing long-range temporal dependencies more effectively than recurrent models in some regional datasets^21^, although broader comparative studies indicate that the optimal architecture may vary across regions and catalog characteristics^22^. More advanced frameworks, including the Earthquake Prediction Transformer (EPT), have combined attention mechanisms, gated feature extraction, and imbalance-aware loss functions to improve mainshock prediction^23^. Hybrid CNN–BiLSTM–attention models have also been used for short-term regional forecasting of earthquake magnitude and frequency^24^. In addition, frequency-aware architectures based on discrete cosine transform channel attention have shown the benefit of explicitly modeling frequency-domain patterns in time-series forecasting^25^. These studies collectively support the need for systematic benchmarking of multiple forecasting architectures under consistent preprocessing and evaluation conditions.

Data quality and preprocessing are important in catalog-based forecasting because seismic catalogs may contain aftershock clustering, spatial heterogeneity, and uneven event distributions. Catalog declustering methods based on kernel density, inter-event statistics, and spatial-distance analysis have been used to separate background seismicity from clustered events, improving the stability of long-term seismic analysis^26^. Related studies in spatial seismic regression and hazard modeling have also shown that spatial relationships and regional transferability can influence predictive performance, as demonstrated by graph neural networks for intensity prediction and ensemble learning for peak ground acceleration estimation^27,28^. These findings highlight the importance of standardized preprocessing, spatial-temporal representation, and consistent evaluation protocols.

Although not the main focus of this work, precursor-based studies remain relevant as part of the broader earthquake-prediction literature. Soil radon-based studies have used boosted trees, support vector machines, and k-nearest-neighbor regression for anomaly detection^29^, while stress-cycle proxy modeling has used correlation-based indicators, Receiver Operating Characteristic analysis, and entropy measures to characterize stress accumulation and release patterns^30^. Ionospheric and atmospheric precursor studies have employed encoder–decoder LSTM models for total electron content prediction^31^, real-time TEC disturbance classification using machine learning^32^, Swarm satellite-based LSTM and voting strategies^33^, and multi-sensor atmospheric anomaly analysis^34^. These studies are summarized briefly here because they use non-catalog precursor signals, whereas the present work relies on historical earthquake catalog observations.

Machine learning has also been applied in operational earthquake-related systems and hazard assessment. Ensemble earthquake early-warning systems have used short P-wave windows for rapid detection, magnitude estimation, and localization^35^, while unsupervised autoencoder-based approaches have reduced false alarms in seismic event detection^36^. Other studies have addressed coseismic deformation extraction using autoencoder-based InSAR denoising^37^, post-earthquake structural capacity estimation using decision-tree surrogate models^38^, transfer learning for data-scarce seismic settings^39^, and IoT–edge–cloud monitoring frameworks for low-latency seismic applications^40^. Although these studies differ from the present catalog-based forecasting objective, they demonstrate the broader importance of model generalization, computational efficiency, and deployment-oriented evaluation.

Beyond catalog forecasting, inverse modeling and structural-response reconstruction studies provide additional methodological evidence for the value of deep temporal models. Recent work on vibration-based damage detection has emphasized intelligent, automated, and sensor-efficient frameworks while identifying challenges related to noise, interpretability, and real-world generalization^41^. Deep neural models have reconstructed ground-motion histories from structural responses^42^, while attention-enhanced and multi-scale methods have improved full-profile seismic response reconstruction under sparse sensing conditions^43^. Multi-head attention temporal convolutional models have further addressed long-range dependency and non-stationarity in seismic inversion tasks^44^. These studies are retained in condensed form because they support the methodological relevance of deep temporal modeling for noisy and incomplete seismic data.

Hyperparameter optimization is another key factor in data-driven seismic modeling. Model performance is often sensitive to parameters such as learning rate, batch size, network depth, temporal window length, and regularization strength. Prior catalog-based studies have shown that optimizing temporal and magnitude-related parameters can improve earthquake forecasting performance^17^. In related nonlinear seismic and structural modeling tasks, LS-SVM combined with swarm-based optimization has achieved strong performance^45^, and RBF networks optimized by evolutionary strategies have demonstrated competitive nonlinear inference capability^46^. Together with the strong performance of attention-enhanced LSTM and TCN-based models in seismic reconstruction tasks^42–44^, these findings justify the inclusion of representative benchmark models such as RBF, LS-SVM/LSSVM, SVM, LSTM, Transformer-based models, and TCN-like architectures in the present study.

Overall, the literature demonstrates substantial progress in ML- and DL-based earthquake research, but several limitations remain. Many studies focus on isolated tasks, single model families, limited datasets, or inconsistent evaluation protocols, which restricts fair comparison across forecasting architectures and optimization strategies. The present work differs from precursor-only, operational early-warning, and structural-response inversion studies by focusing specifically on catalog-based temporal seismic forecasting using historical seismicity records. Moreover, instead of evaluating a single architecture or optimizer in isolation, the proposed framework benchmarks multiple forecasting models and metaheuristic optimization algorithms under consistent preprocessing, training, and evaluation conditions.

### Summary of research gap

The main research gap is the lack of a unified and reproducible benchmarking framework for catalog-based seismic forecasting that jointly evaluates model architecture and hyperparameter optimization. Existing studies demonstrate the usefulness of deep learning, statistical modeling, preprocessing strategies, and optimization methods, but they often differ in datasets, feature preparation, training procedures, and validation settings. Consequently, the individual contributions of forecasting architecture, hyperparameter configuration, and optimizer behavior remain difficult to isolate.

Therefore, the present study addresses this gap by developing a controlled benchmarking framework that integrates multiple deep learning architectures and metaheuristic optimization algorithms within a consistent experimental setting. This design enables fairer comparison of forecasting models, clearer interpretation of architecture–optimizer interactions, and more reproducible assessment of catalog-based seismic trend modeling.

Table  1 provides a concise synthesis of the reviewed studies. To maintain citation coverage while improving focus, the table preserves all studies cited in the original section, but the surrounding discussion has been shortened and reorganized around catalog-based forecasting, deep temporal modeling, preprocessing, operational relevance, and hyperparameter optimization.

**Table 1:** Concise comparative synthesis of the reviewed studies.

Ref.	Focus area	Methodology	Key contributions
^29^	Soil radon precursor	Ensemble/individual ML regression (boosted trees, SVM, KNN); repeated CV	Nonlinear ensembles effectively model radon variability and improve anomaly detection linked to seismic activity.
^30^	Stress-cycle proxy modeling	Correlation-based proxies; ROC; entropy analysis	Comparable information content for stress accumulation/release imaging from microseismic patterns.
^16^	ML in seismology survey	Systematic review across catalogs, ground motion, deformation	Identified advances and challenges (imbalance, validation), framing ML tasks in seismic workflows.
^28^	PGA prediction	Hybrid SeisEML ensemble; cross-region evaluation	Reduced PGA error vs attenuation models; demonstrated regional transferability.
^17^	Strong EQ forecasting	Multi-timescale parameter optimization; clustering; AUC maximization	Improved Mw$\ge$6.5 prediction via adaptive temporal/magnitude tuning.
^35^	Early warning (single-station)	Ensemble ML on 3 s P-wave features	Accurate detection and rapid magnitude/location estimation with minimal sensing.
^31^	TEC precursor modeling	Encoder–decoder LSTM; anomaly thresholds	Enhanced TEC forecasting and reliable ionospheric anomaly detection.
^32^	Real-time ionospheric detection	VARION dsTEC/dt + RF/XGBoost	Effective TEC perturbation classification with real-time feasibility.
^27^	Spatial seismic regression	Graph neural network (TISER-GCN)	Leveraged station topology to reduce MSE in intensity prediction.
^18^	Short-horizon magnitude category	Feature engineering + Random Forest	Accurate 30-day maximum magnitude estimation via ensemble trees.
^21^	Regional magnitude forecasting	Transformer vs LSTM/BiLSTM	Transformer improved multi-step magnitude prediction performance.
^19^	Regional time-series forecasting	ARIMA, SSA, CNN, LSTM comparison	LSTM outperformed classical models for nonlinear regional trends.
^38^	Structural capacity assessment	Simulation (OpenSEES) + ML regression	Decision trees enable rapid post-earthquake capacity estimation.
^39^	Transfer learning	Cross-domain time-series transfer; statistical validation	Improved convergence and performance in data-scarce seismic settings.
^33^	Swarm ionospheric precursors	LSTM + voting anomaly detection	Multi-precursor anomalies detected weeks before major events.
^23^	Global-to-local prediction	Gated features + attention + GHMC loss	Enhanced mainshock prediction while addressing imbalance.
^34^	Atmospheric precursors	Multi-sensor statistical + DL analysis	Detected coherent multi-parameter anomalies pre-event.
^20^	Statistical forecasting	SARIMAX with exogenous variables	Demonstrated value of geological/geodetic covariates.
^25^	Frequency-aware architecture	DCT-based channel attention (FECAM)	Improved time-series forecasting via enhanced frequency modeling.
^26^	Catalog declustering	Kernel density + inter-event statistics	Isolated Poissonian background for stable forecasting inputs.
^40^	IoT–edge–cloud monitoring	Edge Bayesian model + cloud ANFIS	Reduced latency and improved reliability in smart monitoring.
^22^	Multi-region magnitude prediction	LSTM/BiLSTM/Transformer comparison	Optimal DL architecture shown to be region-dependent.
^37^	InSAR deformation extraction	Autoencoder DL denoising	Automated coseismic displacement extraction from noisy data.
^36^	Train earthquake detection	Unsupervised autoencoder vs STA/LTA	Reduced false alarms in safety-critical systems.
^24^	Short-term regional forecasting	CNN–BiLSTM–attention + ZOH	Improved prediction of next-month magnitude and counts.

## Material and methods

**Figure 1:**
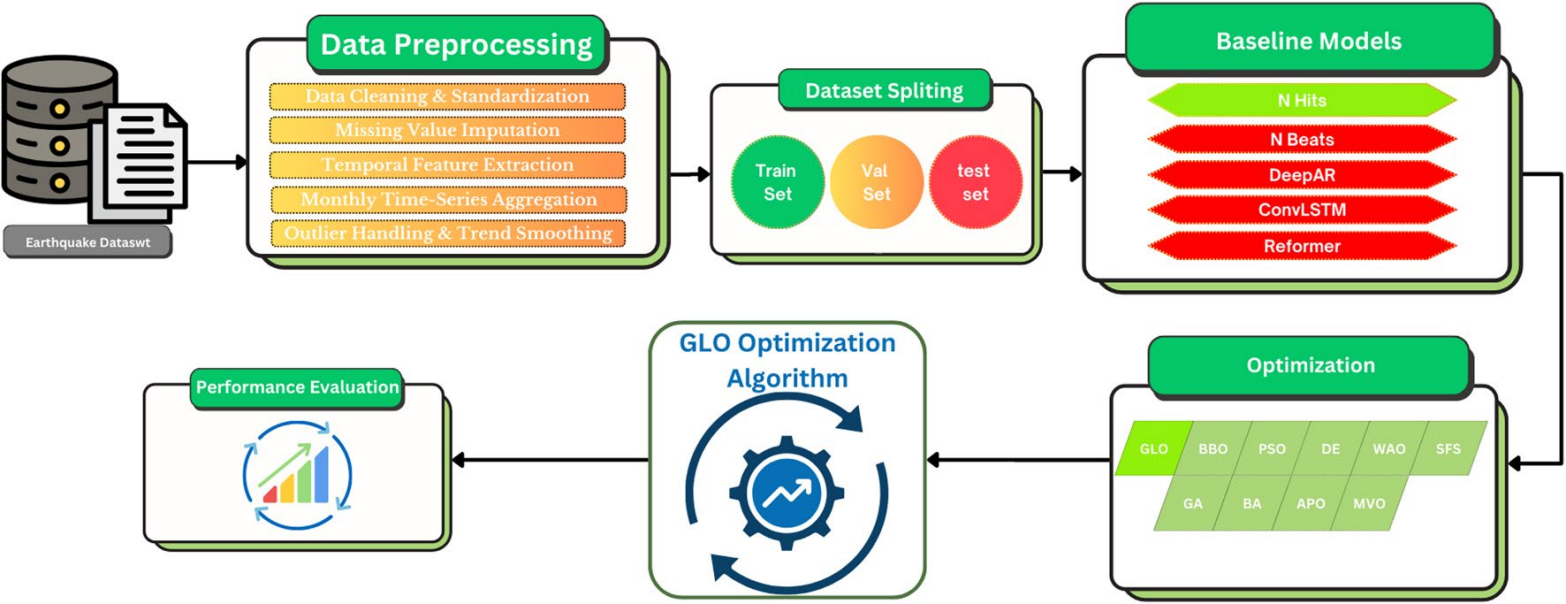
Overall framework of the proposed seismic time-series forecasting methodology, including data preprocessing, dataset splitting, baseline deep learning models, optimization strategies, and final performance evaluation.

In order to give a concise summary of the proposed experimental setup, the general methodology pipeline used in the current study is summarized in Fig. 1. The data preprocessing of the earthquake data starts with a stage of thorough data preprocessing that consists of structural standardization, analysis of missing values, shaping of time features, categorical cleaning, time-series aggregation, and dealing with outliers. After preprocessing, the data is subdivided into training , testing and validation parts so that it is possible to evaluate the models without bias. A number of deep learning architectures are then used as a basic forecasting model, such as the Neural Hierarchical Interpolation of Time Series (N-HITS), Neural Basis Expansion Analysis of Time Series (N-BEATS), Deep Autoregressive Networks (DeepAR), Convolutional Long Short-Memory (ConvLSTM), and Reformer-based architectures. Several metaheuristic optimization algorithms are being investigated in order to optimize predictive performance and reach an optimal search parameter setting. The main optimization strategy used among them is the Gray Langurs Optimizer Optimization-based Learning Optimizer (GLO), and it is compared with a range of known metaheuristics, Particle Swarm Optimization (PSO), Whale Optimization Algorithm (WOA), Genetic Algorithm (GA), Bat Algorithm (BA), Biogeography-based Optimization (BBO), Differential Evolution (DE), Stochastic Fractal Search (SFS), Arithmetic Optimization (APO), and Multi-Verse Optimization (MVO). Lastly, the models that are optimized are evaluated on the basis of extensive performance appraisal measures in order to ascertain forecasting effectiveness, strength, and ability to generalize.

### Dataset description

The seismic record, which was used in the study, was acquired by the Government of Canada in Natural Resources Canada (Earthquakes Canada). The catalog is offered via the comma-separated values (csv) format, and this supports scientific computing environments and enables reproducible experimentation. Its time span spans between 1 January 1985 and 1 January 2020, giving a period of over thirty years of documented seismic activity of the Canadian territory and the surrounding areas. Deep time-series forecasting, in particular, is well suited to this extended temporal horizon that allows both learning short-term variations as well as long-term structural dependencies to seismic sequences.

In this study, the forecasting target is defined as the earthquake magnitude recorded in the seismic catalog. Specifically, each observation corresponds to the magnitude value associated with an individual seismic event. Accordingly, the predictive models are designed to estimate future earthquake magnitudes based on historical seismic observations, rather than predicting event counts or occurrence probabilities (Table 2).

**Table 2:** Summary of the Canadian earthquake dataset features.

Feature	Description	Observed range
Date/time	Event occurrence timestamp	1985–2020
Latitude	Geographic latitude (degrees)	$40.8^{\circ }$ to $83.5^{\circ }$
Longitude	Geographic longitude (degrees)	$-150^{\circ }$ to $-39.3^{\circ }$
Depth	Focal depth (km)	− 2.6 to 214 km
Magnitude	Earthquake magnitude	− 1.9 to 7.7
Magnitude type	Measurement scale identifier	Categorical
Location descriptor	Nearest geographic reference	Textual

The data set is an event level data, and each of the data lines of a table shows an occurrence of an earthquake. Table 1 below, namely tab:dataset features, provides the major features of the dataset. All these properties are the encoders of the temporal, spatial, and geophysical features of seismic events and the basis of built predictive models. As illustrated in Table 21, the latitude range indicates seismic activity stretching further into southern Canada into the high Arctic areas, whereas the longitude distribution is that of the western Pacific margins and eastward intraplate areas. The depths values reveal that most earthquakes are experienced at shallow to intermediate crustal levels, but there are also those that are below 100 km, especially those that are experienced in tectonically active western regions. The magnitude distribution represents the microseismic events that have negative magnitude, which are made possible by sensitive instrumentation networks, and the moderation-to-strong events that extend to magnitude 7.7.

The attribute of magnitude type separates measurement scales of difference, which plays an important part in nurturing constant interpretational coherence spanning seismic records. The location descriptor gives contextual geographic labeling that allows the analysis of regional clustering, whereby the spatial density patterns may be validated qualitatively.

An in-depth statistical analysis of the data shows that there is a great disparity regarding time, magnitude, depth and spatial division.

*Trends of the temporal frequencies.* The number of events is quite different each year. The previous decades show relatively lower documented rates, and the subsequent years show an increase in the rates of identifying the events, which is probably the result of the improvement in seismic measurement tools and coverage. This is a source of temporal non-stationarity, which underlines the need for models with the capability to represent changing seismic regimes instead of assuming the presence of steady statistical properties.

*Magnitude distribution.* The magnitude histogram is highly skewed towards the right, with most of the events falling in the low-to-moderate bracket of magnitude (around 0-3). The frequencies of the events decrease quickly with the magnitude, which is in line with the Gutenberg-Richter relationship. This disequilibrium provides a modeling difficulty, whereby infrequent high-magnitude happenings are associated with inappropriate hazard importance and include somewhat restricted training cases.

*Depth distribution.* The depth histogram shows that the majority of seismic activities are developed in the shallow crustal areas (wholly under 40 km). However, due to the intermediate and deep events, which do go beyond 100 km, there are indicators of multiple seismogenic structures throughout Canadian tectonic provinces. The depth distribution has a multi-modal structure that highlights the significance of keeping depth as a continuum as opposed to discretizing the depth into coarse categories.

Clusters of spatial density. The cluster analysis shows that there is a strong clustering on the western side of Canada, especially on the west of Vancouver Island and British Columbia. Further clusters can also be seen in eastern Canada, such as the Charlevoix Seismic Zone, and scattered activity in the north Arctic. With these spatial density gradients, there is emphasis on tectonic heterogeneity, which provides a reason to incorporate the latitude and longitude directly into the forecasting edifice that can learn spatial influencing scores with a duration negligence.

All in all, the dataset has a good geophysical and geographical variability, extensive temporal coverage and expansive geographic coverage. These qualities render it effective in making deep learning forecasting models and metaheuristic optimization strategies assessment within a consistent national-scale analysis of the seismic.

### Data preprocessing

The preprocessing pipeline was developed to make sure that the data is structurally consistent, statistically robust and coherent over time before developing and assessing models. Seismic catalogs are generally heterogeneous in nature, with a combination of continuous geophysical data (e.g., magnitude and depth), spatial (latitude and longitude), and categorical data (e.g., magnitude type and location names). Such heterogeneity, absent from implementing attention to preprocessing, may contain inconsistencies that could sweep forward into model training and worsen its convergence stability, as well as its generalization. Based on that, all preprocessing steps were applied to achieve two complementary goals in a controlled and sequential development: (i) maintain the physical interpretability of seismic observations, and (ii) increase the suitability of data to deep-learning-based time-series forecasting under reproducible experimental conditions.

***Dataset Structure Standardization***

In order to ensure consistency and clarity throughout all the experimental phases, the raw data schema was refined with semantically explicit conventions of naming the data columns. It is a key step towards reproducibility, and this also avoids ambiguity when the feature engineering, encoding and aggregation are done afterwards. Table 3 shows the final standardized structure. A feature could only be maintained when it contained interpretable information regarding seismic characterization or downstream modeling. Specifically, the temporal attribute (date) should be explicitly named to help the Neo4j database be indexed, time-transformed, and named the geospatial and geophysical variables in a manner that would be compatible with the analysis scripts and modeling libraries.

**Table 3:** Standardized dataset column structure.

Column name	Description
date	Event occurrence timestamp
latitude	Geographic latitude (degrees)
longitude	Geographic longitude (degrees)
depth	Focal depth (km)
magnitude	Earthquake magnitude value
mag_type	Magnitude measurement type
place	Location descriptor
province	Canadian province identifier
extra	Auxiliary attribute

As demonstrated in Table 3, the column extra was used as an additional field and did not add any consistent or physically significant information to predict. It was thus dropped to eliminate redundancy, much unwanted dimensionality and complete the feature space. This is a structural refinement that enhances the interpretability and also reduces the chances of spurious correlations affecting the learning process.

***Missing Value Handling***

A missing-value audit was conducted comprehensively to determine completeness on the variables of numerical, categorical and time. It is also essential in seismic catalogs to make sure that missing values are dealt with well because incomplete entries may occur due to instrumentation limitations, late recording or missing standardized metadata formatting.

Primary feature validation was done on essential seismic attributes initially, such as date, latitude, longitude, depth, formation and finally magnitude. These variables constitute the bare possible description of an event. Recordings that had blank values in these fields were reviewed to identify how they would be dropped or rebuilt. There was no deletion that was necessary, and this implies that the primary seismic measurements are very much intact. In numerical variables, any blank records were filled in by the use of n median substitution. Median imputation was chosen as the seismic variables (especially the magnitude and depth) often follow skewness and heavy-tail distributions. It has been seen that the median is a strong central value that is not as sensitive to extreme values and maintains the overall stability of the distribution.

A similar inspection was conducted with categorical variables (mag type, place, province). Mode imputation (the most common class) was used when there were missing values. The decision not to generate artificial rare categories, nor to reject the consistency of encoding, nor to do the same to categories. Upon completion of such procedures, the dataset had a total of 101,365 rows and 0 entries left out. This is critical to deep learning pipelines, whose missing values will raise unstable gradients or implicit bias when treated differently on batches.

***Date-Time Conversion and Temporal Feature Engineering***

The time-series forecasting depends on temporal coherence. The column date was put in a date type to make it easy to have precise indexing, resampling and temporal windowing. On top of type conversion, temporal decomposition was carried out by taking out year, month, day, and hour. These derived features facilitate the description of multiple-scale temporal interactions, making it possible to make a model learn seasonal variations, inter-annual variability, and possible diurnal reporting biases. Although the ultimate forecasting frequency may be monthly, retaining such decomposed elements enhances the exploratory analysis, helps to check further stratification assumptions and allows aggregation functions to be made consistently.

***Province Data Cleaning and Standardization***

The feature province offers a regional identifier, which offers spatial stratification and categorical analysis. Any entries in the province that were missing were substituted with unknown to preserve the structural integrity and avoid losing any samples. Moreover, all the province strings were made uppercase in order to remove artificial duplication that is a result of inconsistent casing (e.g. BC vs. bc). This is necessary before arriving at categorical coding because unstandardized labeling may cause irrelevant division of features as well as decreased statistical power.

***Monthly Time-Series Aggregation***

The raw earthquake is of an event type in which every record is actually a seismic event. In this case, however, the forecasting objective is specified on a time scale that is aggregated. Thus, the data were restructured as a monthly frequency time series by defining resampling of data at a monthly frequency as the index and the monthly event count. Such a transformation is intended to convert irregular event timestamps to a regular time series that can be used in forecasting models based on deep learning, ensuring driving consistency in inputs and outputs in supervised learning.

In order to concentrate on modern seismic activity to decrease historical detection biasness that was related to previous instrumentation and recording methods, time-series modeling only included the records of 2000 and after. The move adds more reliability to the frequency series by highlighting the window during which one can get better catalog completeness and monitoring technology.

***Dataset Splitting***

The prepared dataset was partitioned into three mutually exclusive subsets to ensure a reliable and unbiased evaluation of the proposed forecasting framework. Specifically, 70% of the data was allocated to the training set, which was used to learn the underlying temporal patterns and model parameters. The remaining data was divided equally between validation (15%) and testing (15%) subsets. The validation set was employed during the training phase to tune hyperparameters and prevent overfitting, enabling the optimization algorithm to guide model selection effectively. The test set, which remained completely unseen during both training and validation, was used exclusively for final performance evaluation to assess the generalization capability of the model. This splitting strategy ensures a balanced trade-off between model learning capacity and robust performance assessment, which is particularly important for non-stationary seismic time-series forecasting.

Because the dataset represents a temporal seismic sequence, the splitting was performed in a chronological time-based manner rather than random sampling. Specifically, earlier observations were assigned to the training set, while more recent observations were reserved for validation and testing. This approach prevents information leakage from future events into past data and provides a more realistic evaluation of forecasting performance in real-world seismic applications.

It is important to emphasize that this strict chronological partitioning ensures temporal consistency and avoids the overly optimistic performance estimates that may arise from random data splitting in time-series forecasting problems. While the current study adopts a fixed train–validation–test split for reproducibility and controlled comparison, more advanced validation strategies such as rolling-origin or walk-forward validation could further assess model robustness under evolving temporal conditions and will be considered in future work.

***Smoothing Techniques***

Several smoothing techniques were checked on the frequency series of monthly earthquakes to analyse the dynamics of underlying trends and to minimize high-frequency variations. Analytical, not manipulative, is the purpose of smoothing here; whereas a key use of smoothing is to facilitate interpretation of trend structures and volatility regimes, and one purpose of smoothing is to give an indication of the complexity with time with which forecasting models need to be modelled.

Simple moving averages (SMA) of 3, 6, and 12 months were calculated in order to capture the short-run movements, medium-run movements and long-run yearly or regime-wide tendencies. Exponential weighted moving average (EWMA) has also been used to focus on recent data, and that is especially important in those sequences where sudden changes happen, and the sequences have burst reactions. Both trend and variability were considered as a rolling mean, and a rolling standard deviation was calculated to obtain a volatility-sensitive measure of the evolution of seismic frequencies. The Savitzky -Galley filter was also used as it is a good filter to smooth and then to retain the local peaks and troughs, i.e., the filter is essential in preventing artificial suppression of burst-like seismic clusters. Lastly, the elementary change of the percentage was calculated in order to measure month-to-month momentum and volatility movements, in order to enable rapid changes to be detected, which can pose a challenge to forecast stability.

**Table 4:** Statistical summary of monthly earthquake frequency.

Mean	241.35
Standard deviation	141.30
Minimum	54.00
Maximum	1348.00

Table 4 summarizes the statistical characteristics of the resulting monthly series. Such values give a quantitative seabed of comprehending variability and justification of the multi-scale forecasting style. According to Table 4 The standard deviation is large with respect to the mean, representing strong month-to-month variability. The wide disparity between maximum and minimum further indicates bursts of seismic events, which is why forecasting models that would help in the analysis of non-linear processes and abrupt regime change, as well as the clustered effects of the time scale, are necessary.

***Outlier Detection and Treatment Strategies***

There are valid extreme observations included in seismic catalogs, like the large magnitude events or deep earthquakes, which are often not common, but they are physically significant. Therefore, the outlier processing should be done with caution, not to exclude informative phenomena. Instead of comparing the modeling performance with only one outlier treatment, four strategies were considered and used to measure performance sensitivity to extreme-value treatment. The statistical characteristics of every strategy are highlighted in Table 5.

**Table 5:** Comparison of outlier handling strategies.

Strategy	Rows	Mag mean	Mag std	Depth mean	Depth std
Original	101,365	2.062	0.931	13.59	9.78
Winsorized	101,365	2.061	0.906	13.47	9.26
Capped	101,365	2.067	0.918	13.59	9.78
Removed	73,121	2.072	0.896	13.01	9.41

The Winsorized technique limits the extremities of distributions to distributional percentiles, while preserving the whole sample size. The capped strategy limits values to physically realistic values, which maintain the sample size and ensure domain consistency. The removal strategy uses a Z-score with the adjusted Z-score criterion to remove identified extremes and makes the dataset smaller with a change in the dispersion aspects. Testing several strategies is relevant to show strength: in case the predictive performance does not show a significant deviation between these treatments, then the forecasting structure can be said to be less reliant on assumptions of extreme-value preprocessing.

In summary, the preprocessing pipeline will create a clean and standardized dataset that will train a reliable deep learning model. Limiting its phases to schema normalization, robust imputation, temporal feature construction, aggregation to a regular monthly sequence and a systematic outlier strategy assessment results in the pipeline providing a statistically consistent and physically understandable baseline to further forecasting as well as optimization experiments.

### Data analysis

**Table 6:** Consolidated summary statistics of earthquake magnitudes by longitude-based region and for the full catalog (1985–2019).

Region (longitude)	Count	Share (%)	Mean ± SD	Median	Min	Max	P95
West coast ($< -120$)	75,410	74.4	$2.10 \pm 0.92$	2.00	− 0.50	7.70	3.60
Central ($$[-120,-80)$$)	6395	6.3	$$2.32 \pm 0.76$$	2.20	− 0.60	5.90	3.60
East coast ($\ge -80$)	19,560	19.3	$1.82 \pm 0.96$	1.90	− 1.90	6.30	3.30
Global (all regions)	101,365	100.0	$\mathbf {2.06 \pm 0.93}$	–	− 1.90	7.70	–

Mean magnitudes are reported as $\mu \pm \sigma$.

Notes: Date range: 1985-01-01 11:01:00+00:00 to 2019-12-31 23:54:02+00:00. P95 denotes the 95th percentile of magnitudes. Negative magnitudes can occur for very small events depending on the magnitude scale and reporting convention.

**Figure 2:**
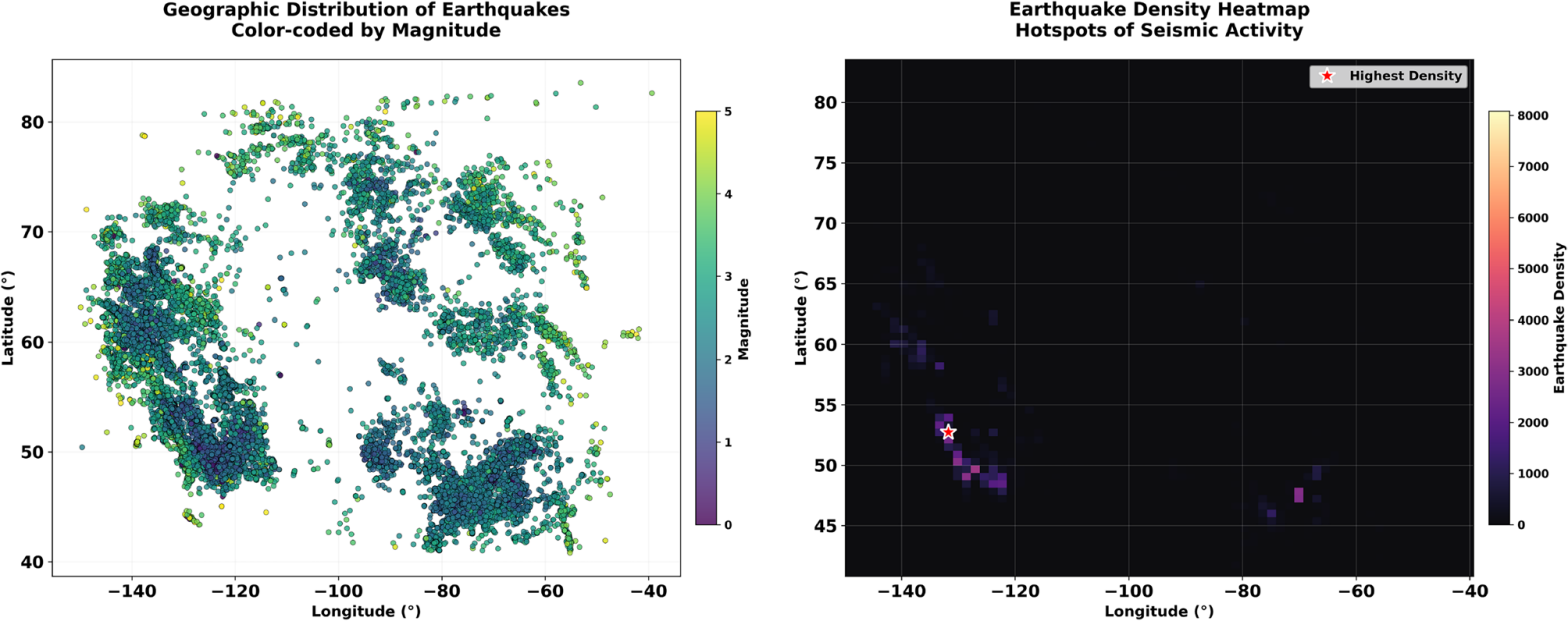
Spatial distribution of earthquakes. **Left:** event locations color-coded by magnitude. **Right:** earthquake density heatmap identifying hotspots of seismic activity (highest-density cell marked).

On top of the visualization of seismicity (space), we are summarizing the statistics of the magnitude of earthquakes with the use of longitude-based regional division (West, Central, East). With this consolidation, it is possible to have an interpretable comparison of seismic productivity (number of events) and magnitude properties (central tendency and characteristics in the upper-tail using the 95th percentile). The present distance heterogeneity of earthquake occurrence and magnitude distributions. Sometimes, such descriptive statistics can be applied in earthquake catalog studies to describe spatial heterogeneity in the occurrence and magnitude distributions of earthquakes that are based on standardized catalog products (e.g., ComCat) and similar measures of magnitude (e.g., moment magnitude when available) (Table 6). Figure 2 combines two orthogonal spatial representations: (i) a point-based geographic density of events represented by colour-coded magnitude and (ii) a density heatmap of hotspots in seismicity. The explanations presented in the above description find this dual representation important due to its value to highlight both the event-level variability (including magnitude heterogeneity) of information in scattered maps and the clustering and spatial pattern of concentrations in density maps that can be less evident in the immediate point clouds. This combination is consistent with the conventional methods of earthquake catalog data visualization.

One of the fundamental procedures in the process of exploratory seismicity analysis is the quantification of the variation in the earthquake occurrence by the varied aggregation scales. Daily, weekly, monthly, and quarterly reporting counts are the best way to understand the level of activity in the basement (mean rate), whether there can be bursty clustering (maximum) or whether there are quiescent periods (minimum). Such summaries of frequency are a common analysis in catalog-based studies due to their general interpretable description of frequency variability before modeling, as well as being suitable for time-series preprocessing functions like binning and resampling. The U.S. Geological Survey (USGS) ComCatsystem is the common source of event archiving and retrieval in the case of catalog-driven analyses to enable the same temporal indexing and frequency calculations, which are reproducible.

Table 7 summarizes the frequency statistics provided in the frequency table of the frequency statistics into one, thus, all the time scales can be directly compared in this table. The totals are the same on all scales since they represent the same underlying event set, whereas averages and extrema are not the same as the aggregation window. The descriptive results put these temporal plots into context, and allow viewing times when activity is the strongest as being concentrated in the counts of bins and not in catalog size.

**Table 7:** Consolidated earthquake frequency statistics across multiple time scales for the analyzed catalog.

Time scale	Average	Maximum	Minimum	Total
Daily	7.93	166	0	101,365
Weekly	55.48	628	10	101,365
Monthly	241.35	1348	54	101,365
Quarterly	724.04	3025	282	101,365

Notes: Frequencies are computed by binning earthquake origin times into non-overlapping calendar intervals (day, week, month, quarter) and counting events per bin. This approach is consistent with standard time-indexed catalog aggregation practices in seismology.

**Figure 3:**
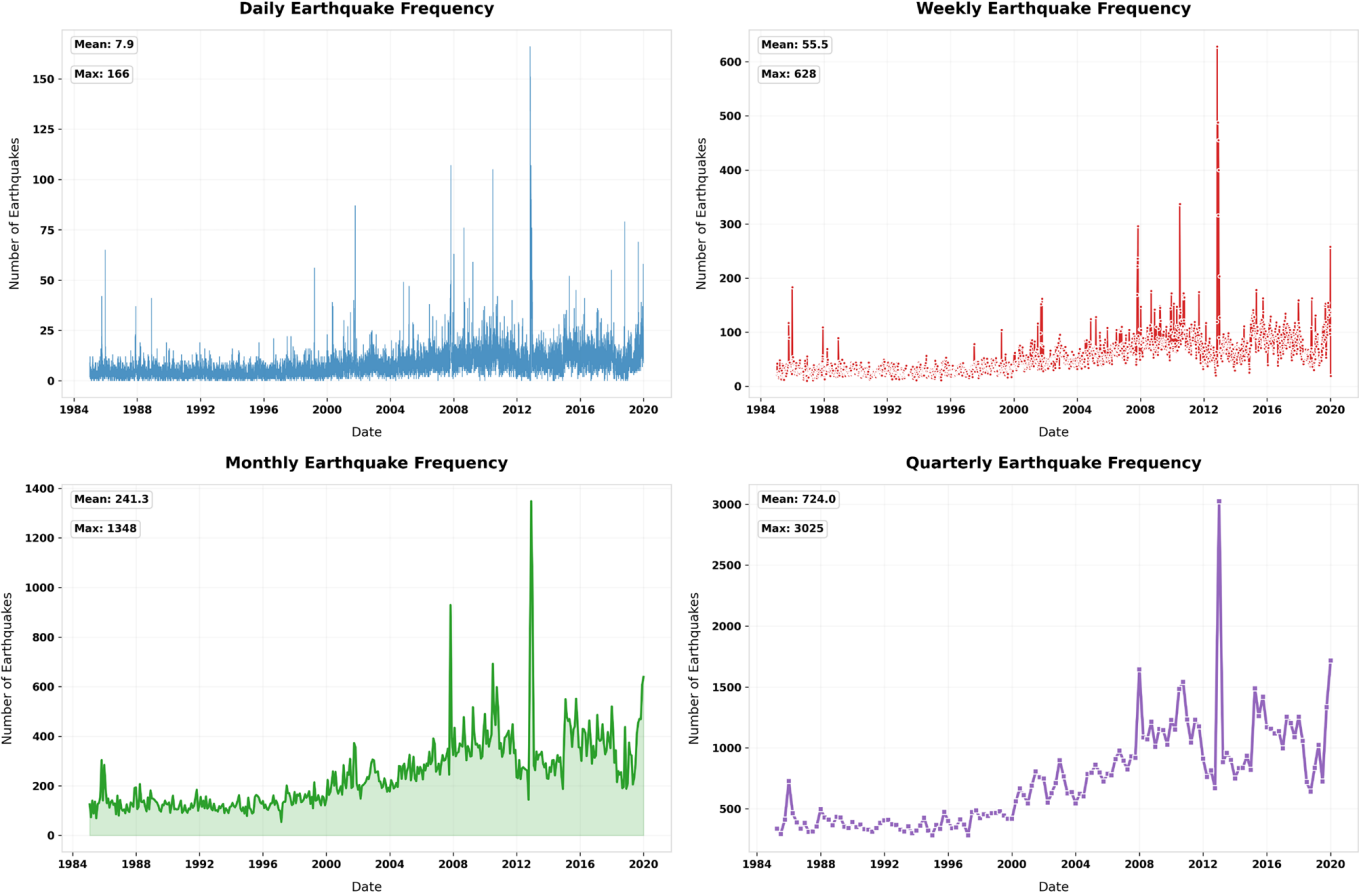
Earthquake frequency time series at daily, weekly, monthly, and quarterly aggregation scales for the analyzed catalog.

Figure 3 plots the same frequency data as a time series at four aggregate levels. The daily panel underlines the high-frequency variability at the moment with short intervals and sharp spikes, but weekly and monthly displays regularize the high-frequency variability and give more focus on the trends on the multi-year scale. The quarterly panel also presents the consistent regime changes and significant clustered periods. Simultaneous demonstration of these resolutions comes in handy when analyzing scale-dependent trends prior to choosing a suitable modeling horizon or making granularity forecasts.

To investigate interannual variability with seismics behavior, earthquake magnitudes were summarized in every calendar year from 2005 to 2019. The aggregate per annum gives an understanding of the temporal changes in the central tendency, dispersion and the occurrences of extreme events. These annual summations are common in seismic catalog investigations to evaluate alterations in magnitude distributions and also to observe years that have high seismic energy or greater percent fluctuation.

**Table 8:** Consolidated yearly earthquake magnitude statistics (2005–2019).

Year	Count	Mean	Median	Std Dev	Min	Max
2005	3171	1.75	1.60	0.99	− 1.60	5.90
2006	3553	1.84	1.80	0.95	− 0.40	5.80
2007	4318	1.87	1.70	0.77	− 0.80	6.00
2008	4379	2.21	2.10	0.95	− 0.60	6.50
2009	4557	2.02	2.00	0.79	− 0.70	6.50
2010	5409	1.99	1.90	0.78	− 0.90	5.40
2011	4361	2.06	2.00	0.80	− 1.00	6.30
2012	5271	2.35	2.30	0.88	− 0.50	7.70
2013	3490	2.24	2.20	0.89	− 0.60	7.50
2014	3424	2.24	2.20	0.85	− 0.80	6.50
2015	5336	2.11	2.10	0.81	− 1.00	6.30
2016	4411	2.03	2.00	0.81	− 1.40	5.50
2017	4806	2.18	2.10	0.77	− 0.90	6.30
2018	3253	2.13	2.00	0.84	− 0.70	6.80
2019	4800	2.05	2.00	0.77	− 1.00	6.70

**Figure 4:**
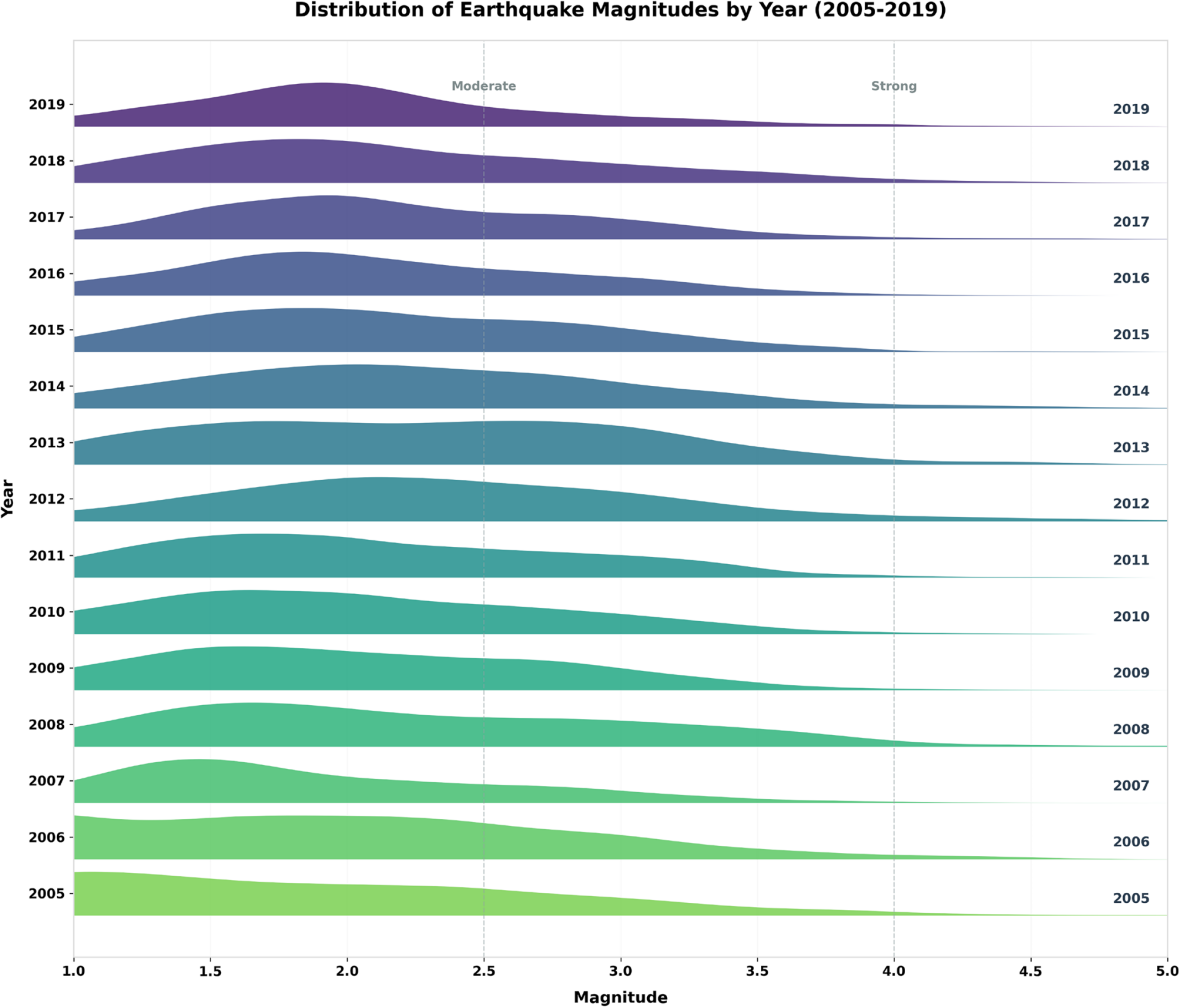
Distribution of earthquake magnitudes by year (2005–2019). Density curves illustrate annual magnitude variability and highlight moderate and strong magnitude thresholds.

Table 8 summarizes all annual statistics into a unified table, allowing a direct comparison according to the years. The table gives figures of the number of events, the mean and median magnitude, standard deviation and the lowest and highest magnitude observed per year. The structure enables the identification of the years of high seismic productivity, greater average magnitude, or the abnormally strong intensity events. In Fig. 4, the magnitude distribution during the year was shown using smoothed density profiles. This graph complements the numerical table to show changes in production distribution shape and trend, spread and tail. Those vertical reference lines denote thresholds of magnitude in relation to moderate and strong events, which are easy to interpret in terms of the frequency of larger magnitude events in this or that year.

The combined table and figure of distribution serve as a good analytical view of the interannual seismic variability that portrays several years that have larger magnitude averages or a more divergent distribution.

**Figure 5:**
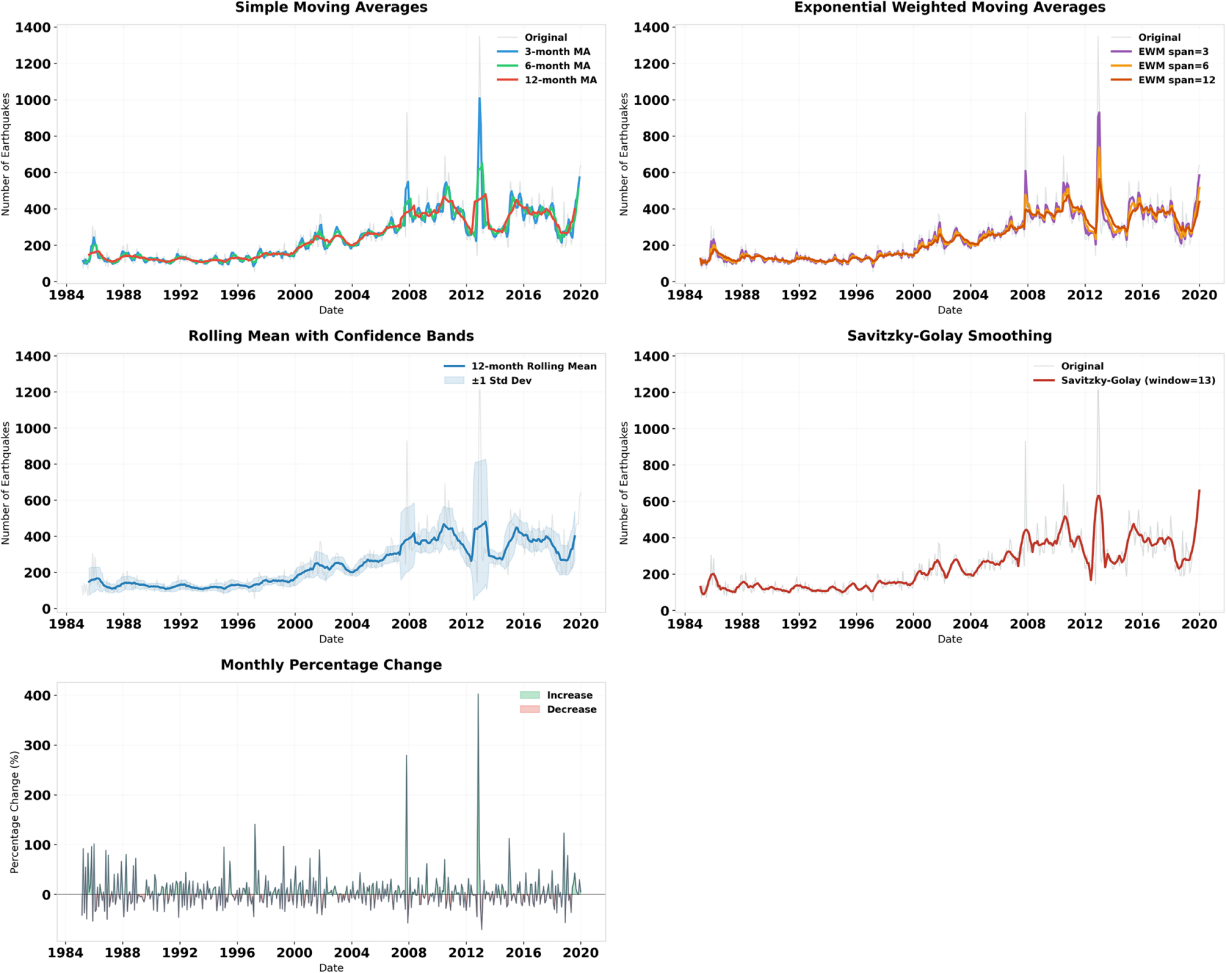
Monthly earthquake-frequency smoothing and volatility diagnostics using multiple techniques (SMA, EWM, rolling mean with variability bands, Savitzky–Golay smoothing, and percentage change).

The fundamental features of earthquake-count time series include high variance caused by connected bursting, high-scale structure due to catalog completeness effects, aftershocks association and background-rate variability in the long term. In order to have the strength of the interpretation and later models, it is thus usual to utilize the smoothing and transformation strategies that can reveal the latent trends without having such plausible, diagnostically crucial attributes as peaks, regime change, and volatility. Other methods, used in both time-series analysis and also tweezing methods in other contexts, are moving-average and exponential-smoothing families to remove high-frequency noise, and rolling statistics (e.g., mean with dispersion bands), which measure uncertainty and stability in time. Polynomial-based filters, which include Savitzky-Golay, are frequently used where local extrema are required, and percentage-change transformations will offer a direct representation of momentum and volatility relative to each other. Table 9 summarizes all the descriptions of smoothing techniques, descriptive statistics of the starting monthly series and provides them in one consolidated table. This makes the methodological aspect of each technique recorded along with the underlying scale and variability of data that is necessary in examining the significance of the smoothed trends, as well as the interpretation of the volatility spiking. Figure 5 plots all the smoothing and diagnostic perspectives of the monthly epidemic-frequency series, such as simple moving averages, exponentially weighted moving averages, rolling mean and uncertainty bands, Savitzky-Golay smoothing and the monthly percentage change. The consistent appearance of trends in the shown panel of moving-average and EWM charts suggests cross-techniques validation: trends that are consistent in both charts tend to have a lower likelihood of being created by a specific smoother, whereas the absence of these similarities indicates the existence of on-off spikes of local variability in the percentage-change view that the trend attempts to fit.

**Table 9:** Consolidated summary of smoothing/diagnostic techniques applied to the monthly earthquake-frequency series, including baseline descriptive statistics of the original series.

Technique	Primary purpose/best use case
Simple moving average (3-month)	Short-term trend identification and noise reduction.
Simple moving average (6-month)	Medium-term trend analysis with moderate smoothing.
Simple moving average (12-month)	Long-term trend extraction and attenuation of seasonal components.
Exponential weighted moving average (EWM)	Emphasizes recent observations; responds faster to changes than simple moving averages.
Rolling mean with std dev bands	Visualizes trend with variability/uncertainty using rolling dispersion around the mean.
Savitzky–Golay smoothing	Smooths while preserving local peaks/valleys via local polynomial fitting.
Monthly percentage change	Measures relative volatility and momentum through month-to-month rate-of-change.
**Original Monthly Series Statistics:** Mean $=241.35$, Std Dev $=141.30$, Min $=54.00$, Max $=1348.00$.	

*Notes:* Moving averages and exponential smoothing are canonical approaches for trend extraction in forecasting practice, while Savitzky–Golay filtering is widely used when peak preservation is required

Time series of earthquake counts are often non-stationary and burst-like and misleading this low-level analysis when observed directly in the raw-count space. Deviations relative to (i) the mean over the long term, (ii) a rolling trend estimate, and (iii) a seasonal-adjusted year-over-year (YoY) baseline are more likely to improve quantifying the departures of typical behavior. All these complementary considerations divide absolute anomaly magnitude (mean deviation), short to medium-term irregularity following detrending (rolling-mean deviations) and interannual volatility under placid seasonal positioning (same-month YoY differences). These diagnostics are typically a part of time-series exploratory analysis since they help understand the proportional importance of trend, variability, and episodic shocks before specifying the model. Table 10 summarizes all reported horizon-plot statistic scores into one table, which includes baseline monthly variability (standard deviation and coefficient of variation), detrended variability (standard deviation after subtracting 12 months of rolling mean), and YoY change features (average change, sign balance, extreme differences). The combination of these results allows for a consistent explanation of the fact that the observed instability is either trend-driven, seasonally organized, or contains bursts.

**Table 10:** Consolidated horizon-style diagnostics for monthly earthquake counts (2000–2019), including baseline variability, detrended variability, and year-over-year change characteristics.

Category	Metric (unit)	Value
Baseline (monthly counts)	Mean monthly count (events/month)	241.35
	Monthly std deviation (events/month)	141.30
	Coefficient of variation (–)	0.585
Detrended (12-month rolling mean removed)	Rolling mean std dev (events/month)	80.16
	Remaining variability (% of original std)	56.7
Year-over-year (same month comparison)	Average change (events/month)	7.42
	Positive changes (months)	219
	Negative changes (months)	185
	Max increase (events/month)	1003.00
	Max decrease (events/month)	− 1118.00

Notes: The coefficient of variation (CV) is defined as $\textrm{CV}=\sigma /\mu$, providing a scale-free measure of relative dispersion. The detrended standard deviation summarizes residual variability after subtracting a 12-month rolling mean trend estimate. Year-over-year (YoY) differences compare each month with the same calendar month in the previous year, reducing seasonal confounding

**Figure 6:**
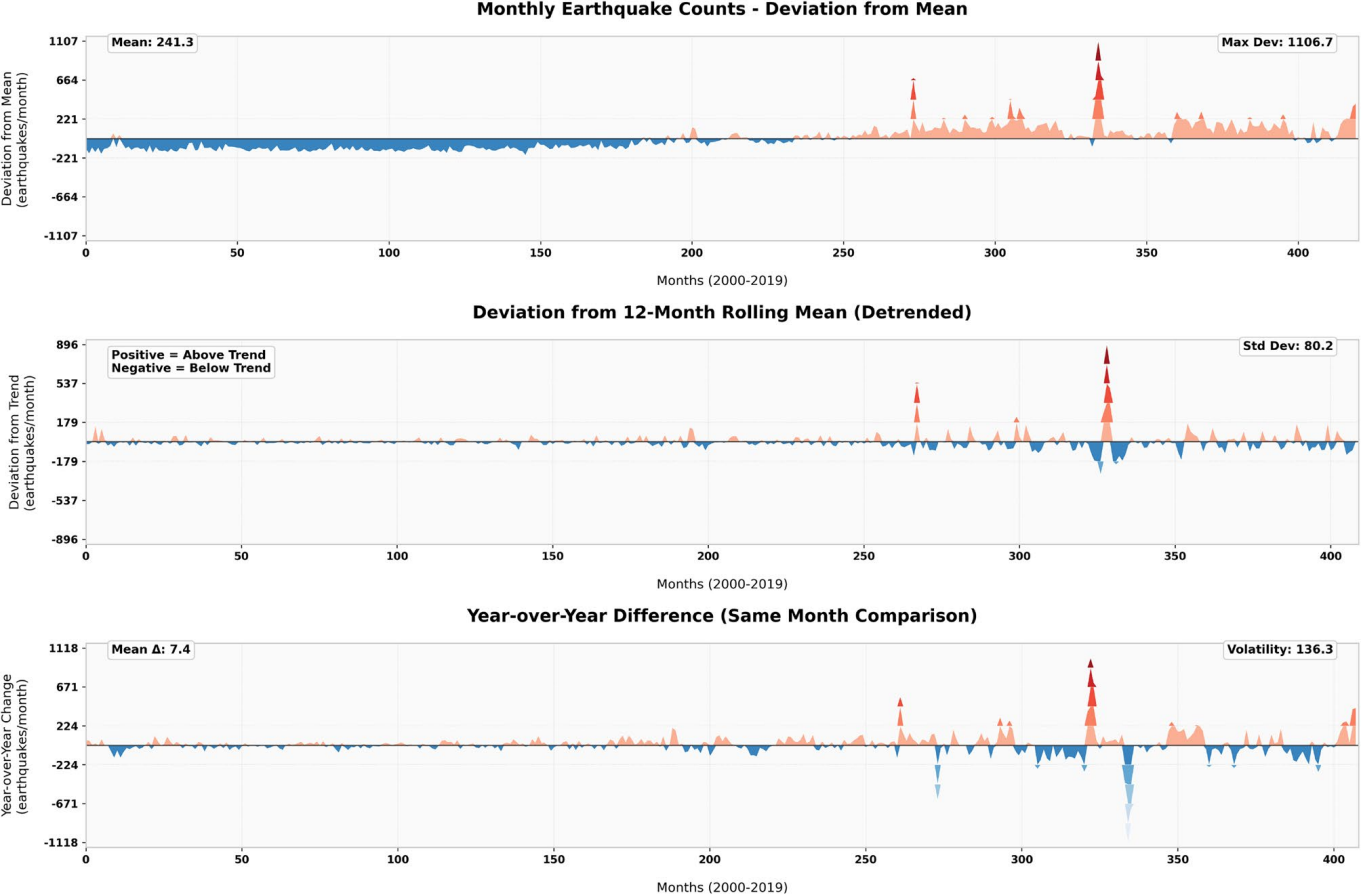
Horizon-style deviation diagnostics for monthly earthquake counts (2000–2019): (top) deviation from global mean, (middle) deviation from 12-month rolling mean (detrended), and (bottom) year-over-year differences for the same month.

Figure 6 presents these diagnostics as three superimposed deviation panels: (i) deviations relative to the world monthly mean, (ii) deviations relative to the 12 months of rolling means (detrended anomalies), and (iii) year-over-year differences by the same month. Positive deviations mark above-baseline activity, of positive deviations whereas negative deviations mark below-baseline activity. Combination: The panels give a systematic interpretation of the anomalies and make a distinction between long-term departures, trend-adjusted changes, and interannual changes.

Extreme or unusual items may be contained in earthquake catalogs either as a result of consciously rare events, reporting bias, location/magnitude bias, or variable network coverage. Since outliers may have a significant effect on descriptive statistics (e.g., mean, variance, kurtosis) in addition to destabilizing data-driven forecasting models, it is natural to compare a variety of outlier-handling methods and measure their effect on both data preservation and distributional regularity. Common methods are winsorization (quantile capping), physically motivated range capping and explicit capping, depending on strong outlier scores (e.g., modified Z-score). These are popular in statistical information preprocessing so as to lessen the power of obsolete values without increasing interpretability.

**Table 11:** Consolidated comparison of outlier-handling strategies for earthquake magnitude and depth.

Strategy	Rows	Pres. (%)	Mag $\mu$	Mag $\sigma$	Mag Skew	Mag Kurt	Mag IQR	Mag range	Depth $\mu$	Depth $\sigma$	Depth skew	Depth kurt
Original	101,365	100.0	2.062	0.931	0.242	0.553	1.300	[− 1.90, 7.70]	13.59	9.78	1.28	6.13
Winsorized	101,365	100.0	2.061	0.906	0.204	− 0.124	1.300	[− 0.10, 4.50]	13.47	9.26	0.65	0.50
Capped	101,365	100.0	2.067	0.918	0.349	0.344	1.300	[0.00, 7.70]	13.59	9.78	1.28	6.13
Removed	73,121	72.1	2.072	0.896	0.354	− 0.191	1.300	[− 0.50, 5.10]	13.01	9.41	0.59	− 0.16

Additional quality indicators: Normality score (lower is better)—Original: 0.486; Winsorized: 0.104; Capped: 0.487; Removed: 0.076.

*Strategy definitions:* Original (raw); Winsorized (1st/99th percentile capping); Capped (physically plausible bounds); Removed (modified Z-score outlier removal).

Notes: Skewness and kurtosis summarize asymmetry and tail/heaviness.

**Figure 7:**
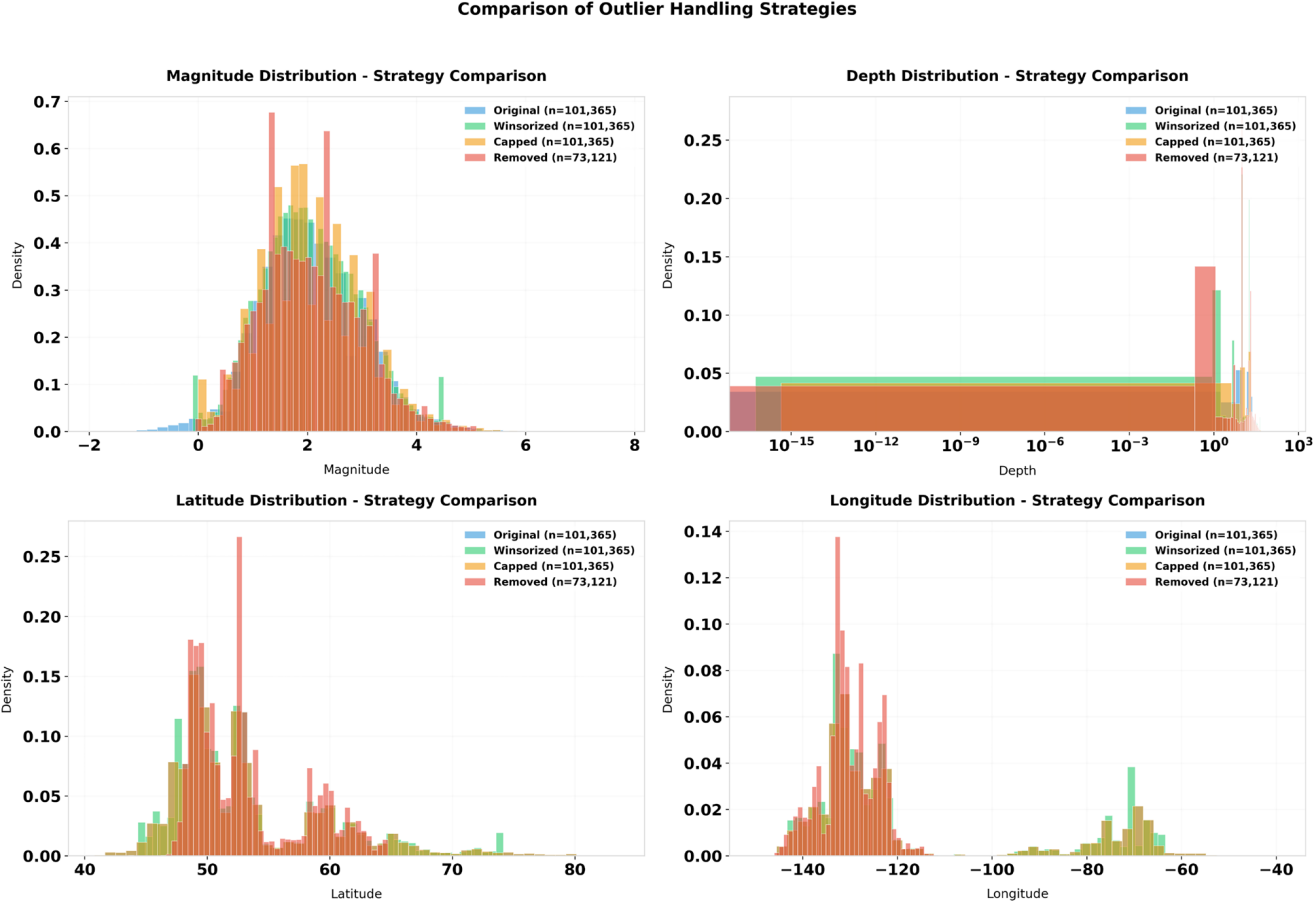
Comparison of outlier-handling strategies via overlaid distributions for magnitude, depth, latitude, and longitude.

Table 11 summarizes all the strategy-level statistics into one table, which presents (i) size retention of the data sets, (ii) first-order statistics of the data sets (magnitude, depth, etc.), (iii) higher-order statistical descriptors (skewness, kurtosis, IQR, range), and (iv) quality measures (data-preservation percentage, normality score which is lower with increasing normality). Such a combined perspective provides a clear opportunity to compare the trade-off attained between distributional regularity and not all the observations are saved. Figure 7 visualizes how each strategy alters the empirical distributions of magnitude, depth, latitude, and longitude. Comparing the overlaid histograms clarifies whether a strategy primarily truncates tails (winsorization/capping) or changes sample composition (removal). In particular, the depth panels are informative for diagnosing whether extreme-depth values dominate higher-order moments, while the geographic panels (latitude/longitude) verify that preprocessing does not inadvertently distort spatial coverage.

**Figure 8:**
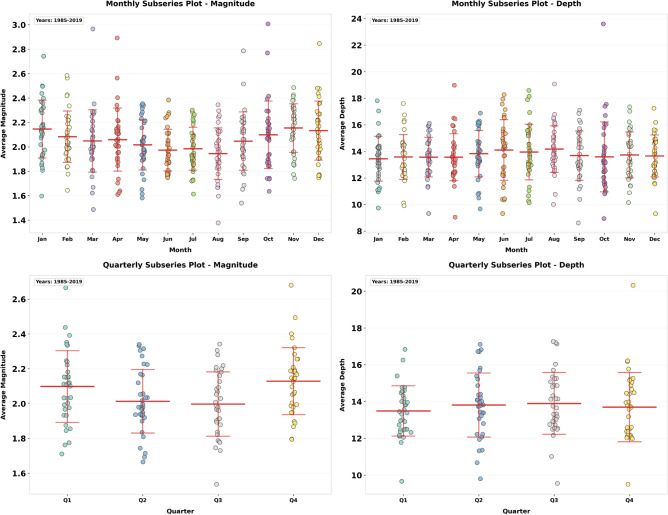
Monthly and quarterly subseries plots for earthquake magnitude and depth (1985–2019). Red horizontal bars represent monthly/quarterly means with variability intervals.

To complement the monthly and quarterly subseries visualizations shown in Fig. 8, all descriptive statistics are consolidated into a single comprehensive table. This unified presentation integrates magnitude and depth statistics across temporal resolutions, enabling direct comparison of central tendency, dispersion, and sample size. The table also highlights seasonal variability patterns, including the most stable and most variable months based on standard deviation of magnitude (Table 12).

**Table 12:** Consolidated monthly and quarterly earthquake statistics (1985–2019).

Period	Mag $\mu$	Mag $\sigma$	Mag *n*	Depth $\mu$	Depth $\sigma$	Depth *n*
Jan	2.183	0.956	8276	13.02	9.43	8276
Feb	2.070	0.945	7582	13.23	9.56	7582
Mar	2.046	0.932	8331	13.31	9.59	8331
Apr	2.053	0.916	7815	13.47	9.46	7815
May	2.005	0.909	7970	13.69	9.71	7970
Jun	1.929	0.892	8418	14.07	9.78	8418
Jul	1.957	0.918	8177	13.69	9.85	8177
Aug	1.928	0.935	8300	13.90	10.17	8300
Sep	2.054	0.942	8500	13.17	10.00	8500
Oct	2.112	0.962	9796	14.05	10.05	9796
Nov	2.192	0.905	9125	13.77	9.97	9125
Dec	2.182	0.903	9075	13.57	9.59	9075
Q1	2.100	0.946	24,189	–	–	–
Q2	1.994	0.907	24,203	–	–	–
Q3	1.980	0.933	24,977	–	–	–
Q4	2.161	0.925	27,996	–	–	–

*Most consistent months (lowest magnitude variability):* June ($\sigma =0.892$), December ($\sigma =0.903$), November ($\sigma =0.905$).

*Most variable months (highest magnitude variability):* October ($\sigma =0.962$), January ($\sigma =0.956$), February ($\sigma =0.945$).

**Figure 9:**
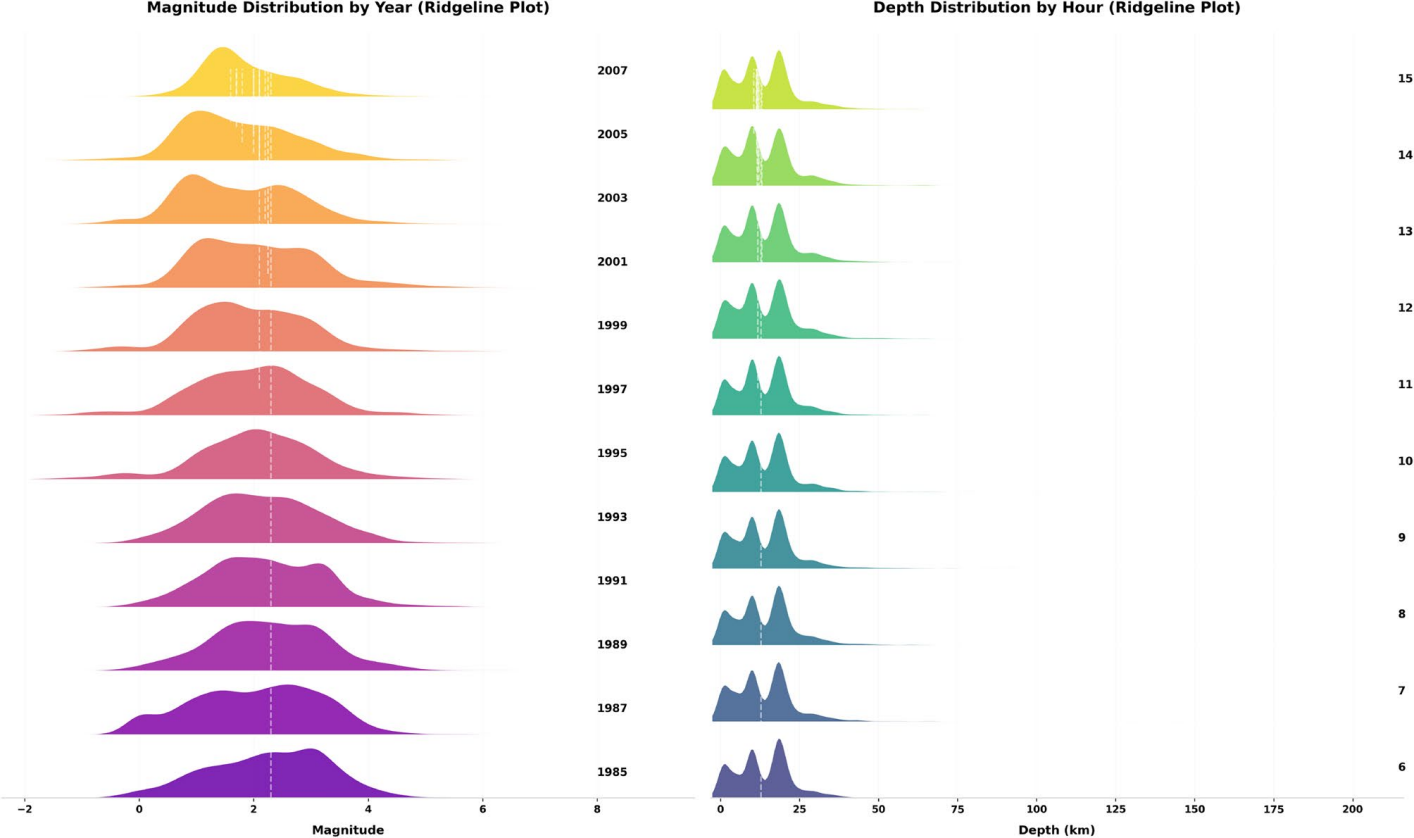
Ridgeline plots illustrating (left) annual magnitude distributions and (right) hourly depth distributions (06:00–21:00). Vertical dashed lines indicate median values within each sub-distribution.

All annual magnitude statistics and hourly depth statistics are stacked into one table to supplement the ridgeline visualizations shown in Fig. 9. This consolidated representation allows the investigation of interannual and intraday variability simultaneously, so that the temporal distribution variability at a wide range of scales can be compared (Table 13).

**Table 13:** Consolidated annual magnitude and hourly depth distribution statistics.

Magnitude distribution by year					Depth distribution by hour (06:00–21:00)				
Year	Mean	Median	Std	Count	Hour	Mean	Median	Std	Count
2008	2.21	2.10	0.95	4379	6	13.85	12.80	9.71	4618
2009	2.02	2.00	0.79	4557	7	13.65	11.82	9.95	4706
2010	1.99	1.90	0.78	5409	8	13.99	13.10	9.80	4589
2011	2.06	2.00	0.80	4361	9	13.67	11.60	9.86	4656
2012	2.35	2.30	0.88	5271	10	13.81	12.20	10.05	4712
2013	2.24	2.20	0.89	3490	11	13.68	12.00	9.42	4360
2014	2.24	2.20	0.85	3424	12	13.86	11.40	10.07	4227
2015	2.11	2.10	0.81	5336	13	13.78	11.60	9.80	4185
2016	2.03	2.00	0.81	4411	14	13.75	10.50	10.20	3982
2017	2.18	2.10	0.77	4806	15	13.39	11.30	9.67	3889
2018	2.13	2.00	0.84	3253	16	13.50	10.52	10.58	3721
2019	2.05	2.00	0.77	4800	17	13.75	11.10	10.45	3765
					18	13.30	11.60	9.62	3827
					19	12.94	10.70	9.38	3925
					20	13.16	11.40	9.39	3818
					21	13.22	10.90	9.79	4015

Notes: Annual magnitude statistics reflect interannual variability in seismic energy release, while hourly depth statistics evaluate potential diurnal patterns in focal depth distribution. Standard deviation quantifies dispersion around the mean, and count denotes the number of recorded events.

**Figure 10:**
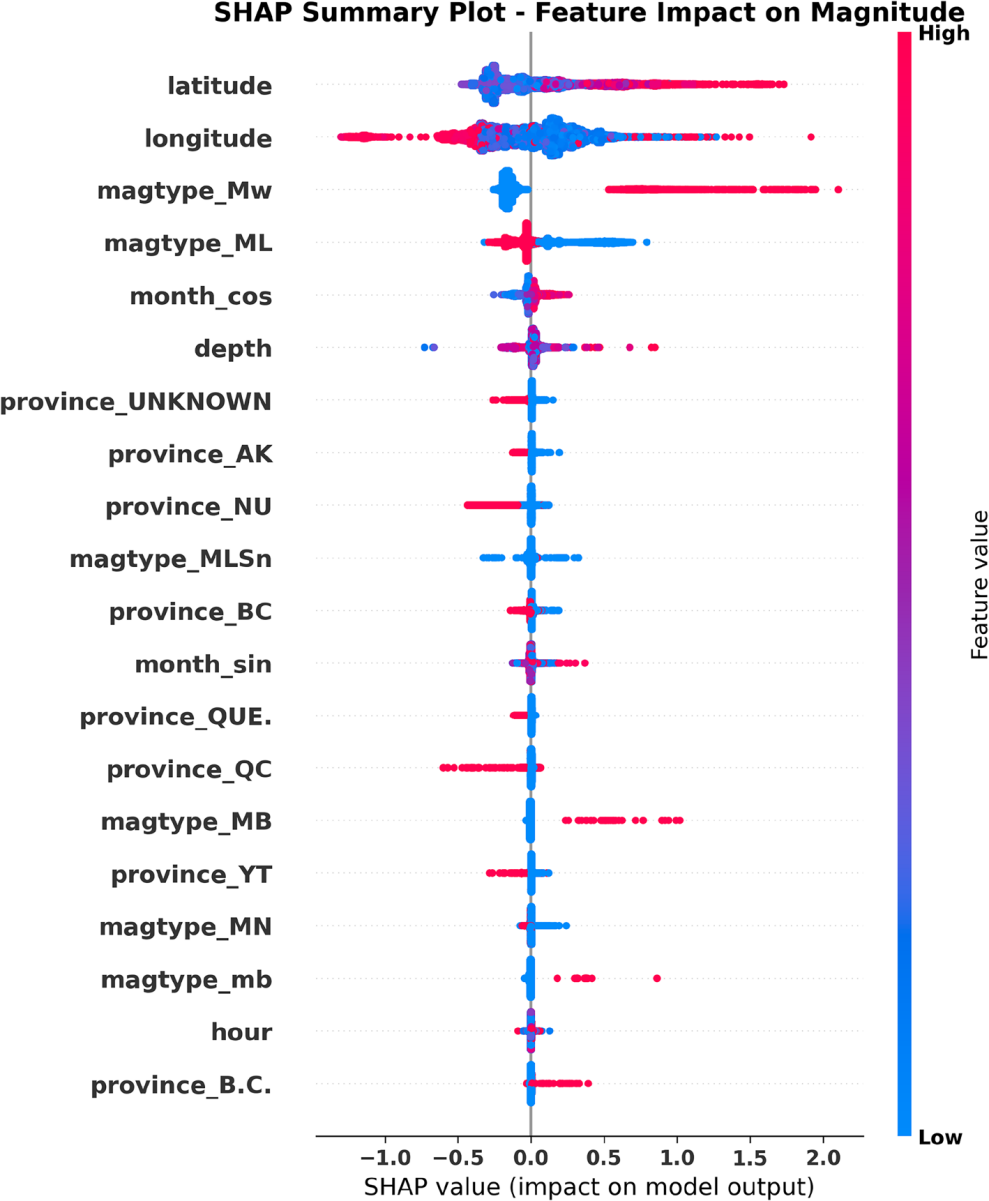
SHAP summary plot illustrating the contribution of input features to earthquake magnitude prediction.

To enhance the interpretability of the proposed predictive framework, SHapley Additive exPlanations (SHAP) were employed to quantify the contribution of each input feature to the model predictions. As illustrated in Fig. 10, the SHAP summary plot presents the relative importance and directional influence of key features in earthquake magnitude forecasting. Features with positive SHAP values increase the predicted magnitude, while negative values reduce it. For example, variables associated with recent seismic activity and higher event frequency tend to increase predictions, reflecting seismic clustering and stress accumulation, whereas features related to lower activity or longer quiescent periods contribute negatively. Furthermore, the spread of SHAP values indicates the strength and variability of feature influence, where wider distributions reflect stronger and nonlinear effects. These results confirm that the model integrates multiple interacting factors rather than relying on a single variable, consistent with the multifactorial nature of seismic processes, and demonstrate that the learned representations capture meaningful geophysical patterns while maintaining model transparency.

### Deep learning models

The forecasting model used in the given study combines deep learning architectures that are particularly focused on nonlinear time-series modeling. Sequences of earthquake occurrences have complicated statistical characteristics, such as clustering in time, non-stationary behavior, heavy-tailed magnitude distribution, and bursts of activity. These features require models that can highly characterize hierarchical time structures, long-range dependencies and nonlinear interactions without using restrictive parametric assumptions.

This resulted in the choice of four deep learning structures to evaluate using the baseline on their proven performance with regard to time-series prediction: N-BEATS, DeepAR, ConvLSTM, and Reformer. Moreover, N-HITS was deployed and subsequently chosen as the reference architecture for metaheuristic optimization, because of its better baseline performance and theoretical applicability of multi-scale forecasting to it.

***N-BEATS***

N-BEATS is a residual deep architecture that is specifically formulated to learn engaging time-series prediction featured by interpretability. It is trained in the function of blocks of stacked fully connected layers organized in forward and backward residual blocks. Every block is taught a basis expansion, which breaks the input signal down into trend and seasonality terms^47^. In contrast to recurrent or convolutional networks, N-BEATS only consists of deep fully connected layers, which makes it easy to build architecturally, but also allows it to express complex functions.

The residual stacking scheme enables a more and more exact forecast to be refined, which means that there is a falsification of redundancy among the blocks. This format is especially beneficial to seismic frequency information, where decomposition of temporal patterns on a hierarchy basis, e.g., seasonal changes or long-term tectonic movement, can be used to make the information more interpretable and predictive.

***DeepAR***

DeepAR is a recurrent neural network based on probabilistic forecasting of time- stanza and is designed as an autoregressive. It uses recursive units as the unit of modeling of sequential dependencies, and at the same time approximates parameters of a predictive probability distribution. DeepAR is autoregressive, which means that past observations directly affect the occurrence of future predictions, but the forecast has continuity in time^48^.

DeepAR can also be described probabilistically, which makes the approach especially useful in seismic forecasting, where quantification of uncertainty is of paramount importance because earthquake of occurrence is a stochastic process. DeepAR is used to predict variation of monthly patterns of seismic frequencies and magnitude through learning conditional distributions instead of deterministic point forecasts. Nonetheless, recurrent architectures can be faced with constraints when representing an extremely long dependency scale when compared to a hierarchical or attention-based architecture.

***ConvLSTM***

ConvLSTM is based on the traditional Long Short-term memory model but adds convolutional operations into the recurrent gating model. This design allows the modeling of spatial and temporal correlations at the same time. On the data that consists of geographic coordinates of the earthquakes, ConvLSTM has the advantage of showing the spatial locality via convolutional kernels while preserving recurrence in a sequence^49^.

ConvLSTM has the potential of learning localized spatiotemporal interactions in the Canadian seismic context, where spatial clustering is clear along tectonic edges and intraplate regions. However, convolutional recurrent models might not be scalable in scenarios where there is a requirement to model long-term horizons without hierarchical decomposition mechanisms.

***Reformer***

Reformer is a transformer architecture that aims to solve the quadratic nature of self-attention mechanisms of traditional systems in terms of their computational complexity. Reformer is able to use a locality-sensitive hashing of approximate attention and reversible layers of the residual (severely limiting the memory usage) and does not require maintaining long-range dependencies^50^.

Attention-based models should be sufficiently well to represent global temporal interactions, which can potentially be due to the delayed stress redistribution. Transformer architectures may, however, be expensive in both data and hyperparameter optimization to converge with permanently unstable sequences like time series of earthquake frequencies.

***N-HITS***

The N-HITS (Neural Hierarchical Interpolation for Time Series Forecasting) model was proposed as a hierarchical extension of the N-BEATS framework, with the objective of improving the efficiency of learning multi-scale temporal representations and long-horizon forecasting. Its design is based on hierarchical signal decomposition, allowing the model to capture both coarse-grained and fine-grained temporal dynamics within a unified architecture^51^.

The architecture consists of multiple hierarchical blocks, where each block is responsible for learning temporal patterns at a specific level of granularity. At the block level, the transformation from input to output can be expressed as:

1 $$\begin{aligned} y_{\textrm{out}}=\textrm{Block}(y_{\textrm{in}})=\textrm{ReLU}(W\cdot y_{\textrm{in}}+b) \end{aligned}$$

where $y_{\textrm{in}}$ and $y_{\textrm{out}}$ denote the input and output of a block, respectively, *W* represents the learnable weight parameters, and *b* is the bias term. This formulation shows that each block performs a nonlinear transformation of the input signal in order to extract informative temporal features.

The hierarchical nature of N-HITS can be understood through a decomposition of the original time series into multiple signal levels and a residual term:

2 $$\begin{aligned} Y=\sum _{l=1}^{L} S_l + R \end{aligned}$$

where *Y* is the original time series, $S_l$ represents the signal captured at level *l* of the decomposition, and *R* is the residual component. This representation reflects the principle that different levels of the architecture specialize in modeling distinct temporal structures of the series.

At each hierarchical level, the corresponding block processes its assigned component independently. The output of the block at level *l* is written as:

3 $$\begin{aligned} y_{\textrm{out},l}=\textrm{Block}_l(y_{\textrm{in},l})=\textrm{ReLU}(W_l\cdot y_{\textrm{in},l}+b_l) \end{aligned}$$

where $y_{\textrm{in},l}$ and $y_{\textrm{out},l}$ are the input and output at level *l*, while $W_l$ and $b_l$ denote the trainable parameters associated with that level. In this way, each block focuses on a particular portion of the time series and contributes to the overall hierarchical representation.

This hierarchical structure is particularly suitable for seismic frequency time series, which often exhibit complex non-stationary behavior across multiple temporal scales. For example, long-term tectonic trends and short-term seismic bursts may be captured at different hierarchical levels, improving the model’s ability to represent temporal variability more effectively.

To ensure a fair comparison among the five forecasting architectures considered in this study, namely N-BEATS, DeepAR, ConvLSTM, Reformer, and N-HITS, all models were implemented using the same preprocessing strategy and standardized training conditions. Following baseline experimentation, N-HITS demonstrated stronger predictive stability and a more effective representation of seismic temporal patterns than the alternative architectures.

Accordingly, N-HITS was selected as the reference model for the second-stage metaheuristic optimization process. In that stage, only the hyperparameters of N-HITS were optimized using state-of-the-art metaheuristic algorithms, thereby isolating the contribution of the optimization procedure while preserving architectural consistency.

The inclusion of different forecasting paradigms, including residual architectures, autoregressive recurrent models, convolutional-recurrent hybrids, and transformer-based attention models, provides a comprehensive benchmarking framework. This systematic comparison enables a rigorous assessment of how architectural design influences earthquake forecasting performance before and after the application of advanced optimization strategies.

### Metaheuristic optimization

Deep learning models like N-HITS are also rich in terms of representational ability, but their predictive capability is extremely sensitive to the hyperparameter settings. Hyperparameters are used to define the structural complexity and learning dynamics of the net before training, unlike model weights, which are learned using gradient-based backpropagation. Misuse of these parameters can all result in under-fitting, over-fitting, unsteady convergence or poor learning in the case of hierarchical architectures. As a result of this, we need systematic hyperparameter optimization to be able to take advantage of the full modeling capabilities of N-HITS in seismic time-series prediction.

The formalization to search the hyperparameter search space in an adaptive and structured way, therefore, used metaheuristic optimization algorithms. These algorithms utilize the population-based search methods that refine candidate solutions correspondingly based on a given fitness function. The visibility of local refinement (exploitation) over global search (exploration) enables metaheuristics to be an effective exploration technique (boosting nearest neighbor score) at navigating the very nonlinear and multimodal environment of hyperparameters of deep learning solutions.

Metaheuristic algorithms have become strong optimization methods in complex, high-dimensional search spaces in which standard deterministic or gradient-based methods might be ineffective. Both the feature selection and the hyperparameter optimization are combinatorial and nonlinear optimization problems in the seismic forecasting context. The uneven spread of the seismic variables, which can be multicollinear features, and the non-stationarity of the time dynamics contribute to the complexity of these tasks even more. The population-based metaheuristic approaches, therefore, give a flexible and adaptive alternative that can explore such hostile search landscapes.

#### Role of metaheuristics in feature selection

The importance of feature selection is to achieve better predictive power, less important computational cost, and easier interpretation. In earthquake forecasting data, the number of features can not be very high, but during the process of interaction between spatial, temporal, and geophysical characteristics, redundancy and noise can emerge. The use of a good subset of features will mean that only informative patterns are captured by the model, and not accidental correlations.

Binary metaheuristic algorithms are most appropriately applied to feature selection problems as these algorithms work in discrete search spaces, whereby each candidate solution is a binary vector indicating the choice of a feature (1) or its rejection (0). The natural Selection of subsets- Combinatorics lends itself to this representation. In contrast to greedy or threshold-based methods of filters, binary metaheuristics are used to assess the feature subsets in their entirety within a fitness function, which frequently carries predictive error and subset size evaluations jointly. Such a multi-guide to adequate ensuring that the algorithm optimizes dimensionality reduction reflects the predictor.

In comparison with standard filtering techniques (e.g., correlation-based ranking, mutual information), using metaheuristic-based feature selection takes into consideration the inter-feature correlations instead of trying to consider each feature in isolation. Filter methods can be calculated very fast, yet can not resolve nonlinear interactions, which are key in seismic time-series modeling. The embedded techniques include feature selection in training the model, e.g., L1-regularized regression, or importance ranking methods based on trees. Although effective in some sense, embedded approaches are usually bound to a particular model architecture and are unlikely to be more broadly applicable than that architecture.

By contrast, metaheuristic feature selection is model-agnostic, and it can optimize a subset of features directly in terms of a desired predictive goal. This can be especially beneficial in the case of nonlinear deep learning models, in which the features can be viewed as uninterpretable, and their importance can be readily noted. Dimensional reduction with preservation of predictive ability makes binary metaheuristics more cost-effect to compute and curb overfitting, as well as enhances the predictability of seismic forecasting models.

#### Role of metaheuristics in model optimization

In addition to the aspects of feature selection, metaheuristics are the main component in the optimization of hyperparameters in deep learning models. Learning rate, network depth, batch size, regularization coefficients and architectural configurations are hyperparameters that play a significant role in determining the training dynamics and ultimate predictive accuracy. The space of hyperparameters is usually, however, large, nonlinear, and in part discrete, resulting in exhaustive search being computationally infeasible.

Metaheuristic optimization is a mechanism giving adaptive space exploration. Search strategies can be applied based on the population, which has the benefit of simultaneously evaluating many candidate configurations, which is more likely to break local minima and find solutions that are competitive on a global scale. Metaheuristics have a greater ability to converge in comparison with grid search or random search approaches, in which sampling patterns are established beforehand.

The role of hyperparameter optimization in deep learning-based seismic forecasting is that it has a direct effect on the convergence time, generalization, and training stability. Correctly tuned learning rates eliminate gradient explosion or vanishing effect, whereas regularization parameters are tuned to decrease overfitting in swamp seismic sequences. Also, balanced architectural parameters contribute towards the higher capacity of the model to support multi-scale temporal patterns without dragging the irrelevant complexity into the model.

One more significant benefit of metaheuristic optimization is the strength, in other words, robustness. It has the ability to search through a set of solutions to find out, at least by a few folds or validation splits of a problem, which of their specific configurations can resist slight changes in the training data. This increases reduced variance and higher reproducibility, which are the key to working seismic hazard monitoring systems.

In general, the metaheuristic techniques have been used as smart search tools that supplement deep learning structures. When applied to earthquake forecasting, they facilitate the systematic control of dimension and hyperparameter-refinement, which eventually leads to predictive reliability and efficiency.

The selection of the Gray Langurs Optimizer (GLO) in this study is motivated by its suitability for the hyperparameter optimization landscape associated with deep learning-based seismic forecasting models. Although several metaheuristic algorithms such as Particle Swarm Optimization (PSO), Genetic Algorithms (GA), Bat Algorithm (BA), Biogeography-Based Optimization (BBO), and Multiverse Optimization (MVO) have been widely applied in similar contexts, their performance can be limited by premature convergence and reduced population diversity when dealing with high-dimensional and non-convex search spaces.

In contrast, GLO introduces a hierarchical population structure combined with an adaptive role-based search mechanism, which enhances both global exploration and local exploitation. Unlike conventional algorithms that rely on uniform update strategies, GLO employs coordinated movement behaviors, including leader-guided updates, stochastic perturbation, and migration, allowing it to maintain diversity while refining promising solutions. These properties are particularly important for optimizing deep neural networks, where multiple interacting hyperparameters create a complex and highly nonlinear optimization landscape.

Furthermore, GLO demonstrates improved convergence stability and reduced sensitivity to initialization, which are critical for ensuring consistent performance across repeated runs. For these reasons, GLO is selected as the primary optimization algorithm in this study, while other metaheuristic methods are included as benchmark baselines to enable a fair and comprehensive comparative evaluation under identical experimental conditions.

#### Gray Langurs optimizer (GLO): mathematical formulation and algorithmic structure

Gray Langur Optimizer (GLO) is a metaheuristic population-based implementation that an imitation of the social structure and social foraging behavior of the gray langur groups. The algorithm replicates group interactions governed by hierarchies, where the role of people is determined by the hierarchical structure (alpha, male, female and child), leadership authority, adaptive searching behavior and migration^52^.

Let *N* denote the population size and *mt* the maximum number of iterations. Each candidate solution $\textbf{X}_i \in {\mathbb {R}}^d$ represents a hyperparameter vector $\boldsymbol{\theta }$ for N-HITS. The fitness of each individual is evaluated using the validation loss . The best solution found so far is denoted by $\textbf{X}_{GB}$ with fitness value $F_{GB}$.

***Population Initialization***

At iteration $t=0$, gray langurs are randomly distributed within the predefined search space:

4 $$\begin{aligned} \textbf{X}_i^{0} = \textbf{X}_{\min } + r_i \odot (\textbf{X}_{\max } - \textbf{X}_{\min }), \end{aligned}$$

where $r_i$ is a random vector uniformly distributed in $[0,1]^d$, and $\odot$ denotes element-wise multiplication.

***Hierarchical Division***

The population is sorted based on fitness and divided into three main groups:

- One-male group
- Multi-male group
- All-male group

Within each group, individuals are assigned roles (alpha, male, female, child) according to fitness ranking.

***Alpha Position Update***

The alpha langur (best individual within a group) updates its position toward the global best using:

5 $$\begin{aligned} \textbf{X}_{\alpha }^{t+1} = \textbf{X}_{\alpha }^{t} + A \cdot (\textbf{X}_{GB}^{t} - \textbf{X}_{\alpha }^{t}), \end{aligned}$$

where *A* is an adaptive coefficient controlling exploitation intensity.

***Male Position Update***

Male individuals update their positions based on dominance interaction:

6 $$\begin{aligned} \textbf{X}_{m}^{t+1} = \textbf{X}_{m}^{t} + B \cdot (\textbf{X}_{\alpha }^{t} - \textbf{X}_{m}^{t}), \end{aligned}$$

where *B* regulates convergence toward the alpha solution.

***Female Position Update***

Female langurs explore around the alpha using stochastic perturbation:

7 $$\begin{aligned} \textbf{X}_{f}^{t+1} = \textbf{X}_{\alpha }^{t} + C \cdot \textbf{R}, \end{aligned}$$

where *C* is a scaling factor and $\textbf{R}$ is a random vector promoting exploration.

***Child Position Update***

Child individuals perform broader exploratory movements:

8 $$\begin{aligned} \textbf{X}_{c}^{t+1} = \textbf{X}_{c}^{t} + D \cdot (\textbf{X}_{\alpha }^{t} - \textbf{X}_{c}^{t}) + E \cdot \textbf{R}, \end{aligned}$$

where *D* and *E* balance guided exploitation and random exploration.

***Autonomy Mechanism***

GLO incorporates an autonomy phase in which all individuals may independently update positions using:

9 $$\begin{aligned} \textbf{X}_i^{t+1} = \textbf{X}_i^{t} + \omega \cdot (\textbf{X}_{GB}^{t} - \textbf{X}_i^{t}), \end{aligned}$$

where $\omega$ controls adaptive convergence pressure.

***Migration Strategy***

To prevent premature convergence, a migration operator is applied based on dynamic parameters $\omega$ and $\varphi$:

10 $$\begin{aligned} \textbf{X}_i^{t+1} = \textbf{X}_i^{t} + \varphi \cdot (\textbf{X}_j^{t} - \textbf{X}_i^{t}), \end{aligned}$$

where $\textbf{X}_j$ is a randomly selected individual, and $\varphi$ determines migration intensity.

***Greedy Selection***

After each update, feasibility constraints are enforced and fitness is recalculated. A greedy selection mechanism retains improved solutions:

11 $$\begin{aligned} \textbf{X}_i^{t+1} = {\left\{ \begin{array}{ll} \textbf{X}_i^{t+1}, & \text {if } F(\textbf{X}_i^{t+1}) < F(\textbf{X}_i^{t}), \\ \textbf{X}_i^{t}, & \text {otherwise}. \end{array}\right. } .\end{aligned}$$

The algorithm iterates until $t = mt$, at which point the global best solution $\textbf{X}_{GB}$ and its fitness $F_{GB}$ are returned. The structured workflow of GLO is formally presented in Algorithm 1. The procedure begins with random initialization of the gray langur population within the predefined search space. After computing the fitness of all individuals and identifying the global best solution $(X_{GB}, F_{GB})$, the population is divided into hierarchical groups. Iterative updates are then performed for the one-male, multi-male, and all-male groups. At each stage, feasibility checks and greedy selection ensure that only improved candidate solutions are retained. Autonomy and migration mechanisms are applied dynamically to enhance exploration and prevent premature convergence. The algorithm terminates after reaching the maximum number of iterations and returns the best solution discovered.


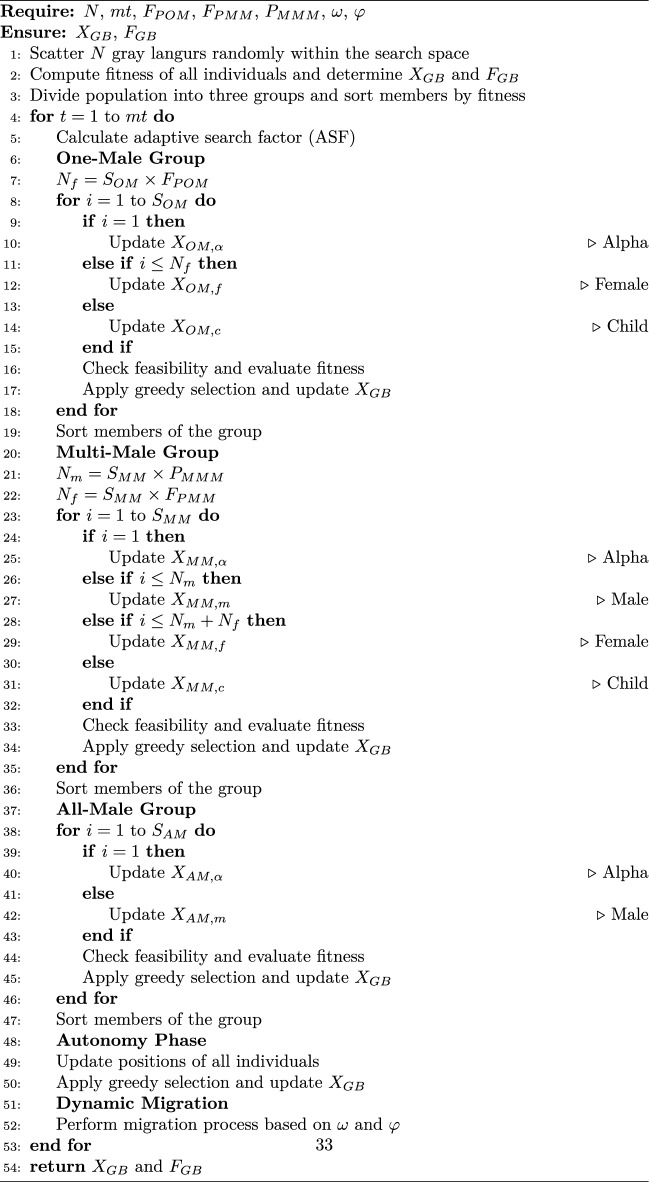


### Problem formulation and hyperparameter optimization setup

This subsection formally defines the seismic time-series forecasting problem, the N-HITS forecasting function, the hyperparameter search space, and the GLO-based optimization objective. Let the preprocessed seismic time series be represented as

12 $$\begin{aligned} {\mathcal {Y}}=\{y_t\}_{t=1}^{T}, \end{aligned}$$

where $y_t$ denotes the forecasting target at time step $t$, and $T$ is the total number of time-indexed observations after preprocessing. Given a lookback window of length $L$ and a forecasting horizon $H$, the input sequence is defined as

13 $$\begin{aligned} \textbf{x}_t=[y_{t-L+1},y_{t-L+2},\ldots ,y_t], \end{aligned}$$

and the corresponding future target sequence is defined as

14 $$\begin{aligned} \textbf{y}_{t+1:t+H}=[y_{t+1},y_{t+2},\ldots ,y_{t+H}]. \end{aligned}$$

Accordingly, the forecasting task is to learn a nonlinear mapping from the historical input window $\textbf{x}_t$ to the future seismic target sequence $\textbf{y}_{t+1:t+H}$.

In the proposed framework, this mapping is modeled using N-HITS as

15 $$\begin{aligned} \hat{\textbf{y}}_{t+1:t+H} = f_{\mathrm {N\text {-}HITS}}(\textbf{x}_t;\boldsymbol{\theta },\boldsymbol{\lambda }), \end{aligned}$$

where $\hat{\textbf{y}}_{t+1:t+H}$ is the predicted output sequence, $f_{\textrm{N}\text {-}\textrm{HITS}}$ denotes the N-HITS forecasting function, $\boldsymbol{\theta }$ represents the trainable network parameters learned during model training, and $\boldsymbol{\lambda }$ represents the hyperparameter configuration optimized by GLO.

The hyperparameter vector is defined as

16 $$\begin{aligned} \boldsymbol{\lambda } = (L,n_s,n_b,n_l,\textbf{q},u,B,\eta ,\rho ,S,a,r,\omega ,\gamma ,p) \in \Lambda , \end{aligned}$$

where $L$ is the input size, $n_s$ is the number of stacks, $n_b$ is the number of blocks, $n_l$ is the number of layers, $\textbf{q}$ is the frequency downsampling configuration, $u$ is the number of hidden units, $B$ is the batch size, $\eta$ is the learning rate, $\rho$ is the dropout rate, $S$ is the maximum number of training steps, $a$ is the activation function $r$ is the interpolation mode, $\omega$ is the weight-decay coefficient, $\gamma$ is the learning-rate decay factor, $p$ is the pooling mode, and $\Lambda$ denotes the predefined mixed discrete-continuous search space reported in Table 14.

The need to ensure reliable data-driven seismic forecasting requires reproducibility and transparency in methodology, especially when model performance is influenced by both architectural design and optimization stability. Therefore, the complete range of N-HITS hyperparameters tested in this work, along with the final configuration selected after optimization, is reported in Table 14. Simultaneously, the structure of the Gray Langurs Optimizer (GLO) is described in Table 15 to clarify the adopted search protocol, convergence controls, and evaluation strategy.

The hyperparameter search was designed within a restricted and interpretable space, following best practices in deep time-series forecasting. Discrete ranges were defined for architectural parameters, including the number of stacks, number of blocks, and number of layers, whereas continuous ranges were used for training and regularization parameters, including the learning rate, dropout rate, weight decay, and learning-rate decay. This structure ensures that the optimization process remains computationally feasible and scientifically interpretable, rather than searching through an unconstrained space that may generate unstable or impractical model configurations.

Table 14 summarizes the N-HITS hyperparameter domains and the final selected values. The selected configuration reflects a balance between representational capacity and regularization strength. Specifically, the lookback window, represented by $L$ or input_size, determines the temporal context available to the model. The hierarchical depth parameters, including $n_s$, $n_b$, and $n_l$, control the expressive capacity of the decomposition process, allowing the architecture to distribute learning across multiple levels rather than relying on a single temporal scale. The downsampling configuration $\textbf{q}$ further supports multi-scale modeling by organizing the temporal resolutions used across the N-HITS stacks.

During training, the learning and regularization parameters were optimized to support convergence stability and generalization. The selected batch size balances gradient stability and computational throughput. The learning rate $\eta$ and learning-rate decay $\gamma$ control the speed of parameter updates, which is particularly important for non-stationary seismic signals with abrupt temporal variations. The dropout rate $\rho$ and weight-decay coefficient $\omega$ reduce overfitting and improve robustness to temporal variability. Finally, the selected activation, interpolation, and pooling settings support stable nonlinear transformation and hierarchical signal reconstruction.

**Table 14:** N-HITS hyperparameter search space and selected configuration.

Parameter/setting	Description/role	Search space/value	Selected/chosen
input_size	Lookback window (lags)	{24, 48, 72, 96, 120}	96
n_stacks	Hierarchical stacks	{2, 3, 4, 5}	3
n_blocks	Blocks per stack	{1, 2, 3, 4}	2
n_layers	FC layers per block	{2, 3, 4, 5}	4
n_freq_downsample	Downsampling factors	{[168,24,1], [24,12,1], [12,4,1]}	[24, 12, 1]
mlp_units	Hidden units	{128, 256, 512, 1024}	512
batch_size	Batch size	{64, 128, 256, 512}	256
learning_rate	Learning rate	[1$\times$10$^{-4}$, 1$\times$10$^{-2}$]	4.7$\times$10$^{-4}$
dropout_rate	Dropout probability	[0.0, 0.5]	0.12
max_steps	Gradient steps	{500, 1000, 2000, 3000}	2000
activation	Activation function	{ReLU, GELU, Tanh, SiLU}	ReLU
interpolation_mode	Interpolation type	{linear, nearest, cubic}	linear
weight_decay	L2 regularization	[1$\times$10$^{-5}$, 1$\times$10$^{-3}$]	3.2$\times$10$^{-5}$
lr_decay	LR scheduler decay	[0.3, 0.9]	0.72
pooling_mode	Temporal pooling	{MaxPool1d, AvgPool1d}	MaxPool1d

For each candidate hyperparameter configuration $\boldsymbol{\lambda }$, the model parameters $\boldsymbol{\theta }$ are trained on the training subset, and the resulting validation error is used as the fitness value. The GLO-based hyperparameter optimization task is therefore formulated as

17 $$\begin{aligned} \boldsymbol{\lambda }^{*} = \arg \min _{\boldsymbol{\lambda }\in \Lambda } J(\boldsymbol{\lambda }), \end{aligned}$$

where $J(\boldsymbol{\lambda })$ denotes the validation-based objective function. Since five-fold cross-validation is used during optimization, the objective function is computed as

18 $$\begin{aligned} J(\boldsymbol{\lambda }) = \frac{1}{K} \sum _{k=1}^{K} \textrm{MSE}_{\textrm{val}}^{(k)}(\boldsymbol{\lambda }), \qquad K=5, \end{aligned}$$

where $\textrm{MSE}_{\textrm{val}}^{(k)}(\boldsymbol{\lambda })$ is the validation mean squared error obtained in the $k$ -th fold using the N-HITS model configured with $\boldsymbol{\lambda }$. Thus, GLO searches the predefined hyperparameter space $\Lambda$ to identify the optimal configuration $\boldsymbol{\lambda }^{*}$ that minimizes the average validation MSE. The final optimized forecasting model is expressed as

19 $$\begin{aligned} \hat{\textbf{y}}_{t+1:t+H} = f_{\textrm{N}\text {-}\textrm{HITS}}(\textbf{x}_t;\boldsymbol{\theta }^{*},\boldsymbol{\lambda }^{*}). \end{aligned}$$

Although Table 14 specifies the model configuration, the effectiveness of the resulting selection depends strongly on the optimization process by which the hyperparameter space is explored. Deep-learning hyperparameter spaces are nonlinear, mixed discrete-continuous, and usually contain multiple local minima. Therefore, population-based optimization is used to improve the probability of identifying effective configurations while maintaining convergence stability.

Gray Langurs Optimizer was configured to provide sufficient exploration of the hyperparameter space while maintaining controlled exploitation around promising configurations. The complete optimization setup is reported in Table 15. A population of 25 agents was used to ensure search diversity while keeping computational requirements manageable. The optimization was executed for 100 iterations, resulting in 2,500 objective evaluations. The objective function was defined as the validation-fold MSE so that the optimizer emphasizes generalization performance rather than training-set fit. In addition, a five-fold cross-validation strategy was adopted to reduce sensitivity to a single validation split and improve the robustness of the fitness estimate.

The initial population was generated using Latin Hypercube Sampling (LHS), which provides broad and stratified coverage of the search space at the beginning of the optimization process. This is particularly useful in seismic forecasting tasks, where model performance can be sensitive to initial hyperparameter choices. The convergence criterion was based on the change in fitness across successive iterations, allowing the optimization process to terminate when no meaningful improvement was observed while avoiding unnecessary additional computation.

**Table 15:** Gray Langurs optimizer (GLO) configuration for hyperparameter tuning.

Parameter/setting	Description/role	Value
Optimization algorithm	Metaheuristic optimizer	GLO
Number of iterations	Optimization cycles	100
Population size	Search agents	25
Objective function	Fitness criterion	MSE (validation fold)
Evaluation strategy	Cross-validation	5-fold CV
Search strategy	Exploration–exploitation	Gradient surrogate + local refinement
Initialization	Population sampling	Latin Hypercube Sampling (LHS)
Convergence criterion	Termination rule	$\Delta$fitness < 1$\times$10$^{-6}$ (15 iterations)
Total evaluations	Function calls	2500 (100 $\times$ 25)

Overall, this formulation explicitly connects the seismic input-output forecasting task, the N-HITS forecasting function, the hyperparameter search space, and the GLO-based optimization objective. Together, Tables 14 and 15 provide a complete and reproducible description of the optimized forecasting model and the adopted optimization procedure, supporting transparent comparison with future optimization-aware deep learning pipelines for seismic time-series prediction.

### Benchmark algorithms

In order to strictly assess the performance of the proposed GLO, nine well-developed metaheuristic optimization techniques were chosen as benchmark methods. These optimizers’ paradigm will include a variety of methodological paradigms such as swarm intelligence, evolutionary computation, physics-inspired search methods and stochastic diffusion mechanisms. The results were generated with all algorithms having the same population sizes, hyperparameter search scales, and termination requirements in order to be fairly methodological. All optimizers were to be used with the validation loss as the fitness function.

**PSO** is a swarm-based optimization algorithm that is based on the flocking patterns observed in fish schools and bird flocks. All of these particles are considered to be a candidate solution and modify their movement depending on their previous greatest experience and the greatest solution observed over the swarm. It is also known that PSO converges very fast and is simple, albeit it tends to premature convergence in search spaces characterized by a high multitude^53^.

**WOA** emulates the hunting of humpback whales with their bubble-net. It switches between a surrounding of the current optimal solution and the spiral shape search patterns of the area. The adaptive exploration/exploitation enables the WOA to weigh changing global and local search, thus it is applicable to the nonlinear optimization problem^54^.

**GA** It is an algorithm in evolutionary computing that is based on natural selection and hereditary principles. There are selection, crossover and mutation operators used to evolve candidate solutions generations by generations. Solutions of high quality will most probably transmit their properties to the next generations, whereas mutation will promote diversity aimed at preventing stagnation. The popularity of GA is based on its strength and the capacity to operate in both continuous and discrete search spaces^55^.

**BA** is based on echolocation by bats. It actively modulates the frequency, loudness and pulse rate of emission parameters in order to regulate the switch between the exploration and exploitation phases. The adaptive aspect of BA is making it sharpens search around promising solution when keeping the ability to explore the whole world^56^.

**BBO** is a model that is used to describe the migration of species across habitats. The candidate solutions correspond to each habitat with its suitability index, and information exchange is via probabilistic migration. Habitats of high-quality have characteristics of low-quality habitats, which facilitate stable convergence and maintain diversity of the population^57^.

**DE** is an evolutionary population-based optimizer based on the differentiation mutation and recombination approaches. It produces candidate solutions which are generated by mixing weighted differing amongst randomly selected persons. DE has been known to be robust and perform well in continuous optimization problems having complicated landscape^58^.

**SFS** is based on fractal diffusion. It combines local search based on diffusion and stochastic updating processes in order to improve exploration in many-modal spaces. The fractal character of the search increases the coverage of the search space, as well as minimizing the possibility of local entrapment^59^.

**APO** is based on adaptive foraging and interaction behaviour of protozoa. It simulates the competitive and cooperative movement strategies in individuals, and then it actively adapts search directions due to the feedback of the environmental situation. ApO gives priority to adaptive step sizes so as to strike the right balance between convergence and diversity exploration^60^.

**MVO** is premised on cosmological ideas like wormholes, white holes, and black holes. In candidate solutions, the exchange of information occurs randomly, and there are extra operators that enhance searching around a solution that is performing well. Early iterations are defined by intense exploration throughout the globe, and later steps are defined by refined exploitation of MVO^61^.

This framework of benchmark suites offloads the capacity to test the optimization performance of GLO in the process of optimizing the hyperparameters of the N-HITS of the forecasting structure by including swarm intelligence, evolutionary measures, cosmological models and biologically inspired mechanisms into a single experimental framework.

### Evaluation metrics

In order to make the assessment of the performance of the forecasting operation rigorous and statistically transparent, several evaluation metrics were used, which are based on multiple regression. The metrics are a combination of measurements of the accuracy of prediction, systematic bias, scale-normalised error, goodness-of-fit and efficiency in explaining the accuracy by the observed series. The fact that the complementary indicators are included does not allow over-dependence on one criterion, and the advantage of a multidimensional evaluation of predictive behavior is introduced. A summary of the entire list of metrics along with their mathematical version is provided in Table 16. Let $y_i$ denote the observed value, ${\hat{y}}_i$ the predicted value, ${\bar{y}}$ the mean of observed values, and *n* the total number of evaluation samples. Based on these definitions, the regression performance measures presented in Table 16 quantify different aspects of forecasting accuracy.

Particularly, Mean Squared Error (MSE) and Root Mean Squared Error (RMSE) focus on bigger deviation as this penalization is quadratic and thus it is prone to big errors in prediction. Mean Absolute Error (MAE) is a simpler and more interpretable measure of absolute error, which is linear. Relative Root Mean Squared Error (RRMSE) scales RMSE in terms of the observed mean, and can be compared across scales. Mean Bias Error (MBE) measures the systematic errors of overestimation or underestimation. Mean Absolute Percentage Error (MAPE) and Symmetric Mean Absolute Percentage Error (SMAPE) represent the accuracy of prediction in percentage, which makes them easier to understand at different levels of magnitude. Lastly, goodness-of-fit can also be assessed as the Coefficient of Determination (R2 ) and Nash-Sutcliffe Efficiency (NSE), with the latter commonly used in environmental and hydrological modeling scenarios. Table 16 gives the formal mathematical expressions of all metrics.

**Table 16:** Regression evaluation metrics used for model performance assessment.

Metric	Name	Mathematical expression
MSE	Mean squared error	$\text {MSE} = \frac{1}{n}\sum _{i=1}^{n}(y_i - {\hat{y}}_i)^2$
RMSE	Root mean squared error	$\text {RMSE} = \sqrt{\frac{1}{n}\sum _{i=1}^{n}(y_i - {\hat{y}}_i)^2}$
MAE	Mean absolute error	$\text {MAE} = \frac{1}{n}\sum _{i=1}^{n}|y_i - {\hat{y}}_i|$
RRMSE	Relative RMSE	$\text {RRMSE} = \frac{\text {RMSE}}{{\bar{y}}}$
MBE	Mean bias error	$\text {MBE} = \frac{1}{n}\sum _{i=1}^{n}( {\hat{y}}_i - y_i )$
MAPE (%)	Mean absolute percentage error	$\text {MAPE} = \frac{100}{n}\sum _{i=1}^{n}\left| \frac{y_i - {\hat{y}}_i}{y_i}\right|$
SMAPE (%)	Symmetric MAPE	$\text {SMAPE} = \frac{100}{n}\sum _{i=1}^{n} \frac{|y_i - {\hat{y}}_i|}{\left( |y_i| + |{\hat{y}}_i|\right) /2}$
$R^2$	Coefficient of determination	$R^2 = 1 - \frac{\sum _{i=1}^{n}(y_i - {\hat{y}}_i)^2}{\sum _{i=1}^{n}(y_i - {\bar{y}})^2}$
NSE	Nash–Sutcliffe efficiency	$\text {NSE} = 1 - \frac{\sum _{i=1}^{n}(y_i - {\hat{y}}_i)^2}{\sum _{i=1}^{n}(y_i - {\bar{y}})^2}$

MSE and RMSE, as mentioned in Table 16, focus on large deviations because they are squared, and MAE, in turn, gives a linear reference of the absolute error. RRMSE is RMSE divided by the mean of the observed value, which allows a comparison not dependent on the scale. MBE is the measure of systematic overestimation or underestimation. MAPE and SMAPE provide forecast error as a percentage, which is more conducive to decision-making aims, whereas R2 and NSE indicate goodness-of-fit compared to expectation variation in the observed time series; NSE is common in hydrological and environmental modelling modes of prediction and offers an interpretation of predictive ability to efficiency.

### Feature selection evaluation metrics

Besides the predictive performance metrics, the usefulness of the feature selection process was also measured by several statistical indicators of fitness calculated between several independent optimization runs. These measures not only determine the predictive quality of the chosen subsets but also measure the stability, robustness and dimensional compactness of the solutions. All of the metrics that were used to evaluate feature selection are summarized in Table 17 along with their mathematical expression.

Let $F_j$ denote the fitness value obtained in run *j*, and $k_j$ represent the number of selected features in run *j*, over *m* independent runs. Based on these definitions, the statistical indicators reported in Table 17 provide complementary insights into optimization behavior.

Precisely, the measures of Average Error and Average Fitness define the central tendency of the obtained fitness values, taking into account the repetitions of executions. Average Select Size is the mean range size of the sampled feature subsets, which is the tradeoff between predictive accuracy and model size. Best Fitness and Worst Fitness explain the limits of performance in the process of optimization, pointing out the quality level of a solution. Lastly, the standard deviation of Fitness measures the stability of an algorithm by measuring the variation in the performance of the algorithm in different runs. All these metrics make a wholesome evaluation of the observation of the effectiveness and reliability of the feature selection mechanism.

**Table 17:** Feature selection evaluation metrics.

Metric	Description	Mathematical Expression
Average Error	Mean error across runs	$\displaystyle \text {AvgError} = \frac{1}{m}\sum _{j=1}^{m} F_j$
Average Select Size	Mean number of selected features	$\displaystyle \text {AvgSize} = \frac{1}{m}\sum _{j=1}^{m} k_j$
Average Fitness	Mean fitness value	$\displaystyle \text {AvgFit} = \frac{1}{m}\sum _{j=1}^{m} F_j$
Best Fitness	Minimum fitness value	$\displaystyle \text {BestFit} = \min _{j=1,\dots ,m} F_j$
Worst Fitness	Maximum fitness value	$\displaystyle \text {WorstFit} = \max _{j=1,\dots ,m} F_j$
Standard Deviation Fitness	Stability of fitness values	$\displaystyle \sigma _F = \sqrt{\frac{1}{m-1}\sum _{j=1}^{m}(F_j - \text {AvgFit})^2}$

The statistical measures of robustness of feature selection are summarized in Table 17. Measures of the central tendency of optimization performance are given by Average Fitness and Average Error. The size of the average Selects captures the size of subsets of selection. The standard deviation of fitness measures the stability of the algorithm in independent runs, whilst the best and Worst Fitness mark boundaries of the performance of the algorithm. Collectively, these measures assure that the evaluation of feature selection takes into consideration both the accuracy and reliability.

## Empirical results

### Baseline deep learning performance (before optimization)

The subsection shows the performance of the underlying deep learning architectures before any metaheuristic optimization is applied. Each model was trained through the same preprocessing practices, data partition, and training parameters in order to obtain methodological fairness. This baseline evaluation is aimed at determining a reference level of performance and identifying the most promising one to optimize further. The regression measures that were established in Sect. 2.7 were calculated on test data. Table 24 provides a summary of the forecasting performance of N-HITS, N-BEATS, DeepAR, ConvLSTM, and Reformer in all the evaluation criteria (Table 18).

**Table 18:** Baseline performance of deep learning models (before optimization).

Model	MSE	RMSE	MAE	RRMSE	MBE	MAPE (%)	SMAPE (%)	$R^2$	NSE
N-HITS	0.00234	0.0484	0.0381	0.152	0.0042	3.95	4.12	0.921	0.918
N-BEATS	0.00308	0.0555	0.0419	0.175	0.0038	4.42	4.57	0.906	0.903
DeepAR	0.00328	0.0573	0.0437	0.181	0.0046	4.68	4.83	0.900	0.896
ConvLSTM	0.00372	0.0610	0.0469	0.185	-0.0056	5.03	5.14	0.893	0.889
Reformer	0.00385	0.0620	0.0478	0.188	0.0031	5.10	5.21	0.887	0.883

N-HITS recorded the lowest MSE (0.00234) and RMSE (0.0484) as demonstrated in Table 1 until Table 9, meaning that it gave the highest accuracy in prediction when compared with other architecture designs. It also generated the lowest MAE (0.0381) as well as the lowest RRMSE (0.152), which is less relative error magnitude. Regarding the percentage-based metrics, N-HITS achieved the lowest MAPE (3.95%) and SMAPE (4.12%), which proves that it is consistent in the evaluation scale on an absolute and normalized basis. With regard to goodness-of-fit, N-HITS had higher values of both the larger $R^2$ (0.921) and the larger NSE (0.918), indicating better explanatory ability and predictive power compared to the randomly observed variance. These findings demonstrate that hierarchical interpolation mechanism of N-HITS has greater ability in people capturing the non-linear and multi-scale seismic time series of the time series.

N-BEATS was second in most measures, then DeepAR. ConvLSTM and Reformer had relatively high values of error and somewhat low values of goodness-of-fit. Though each of the models performed well on predictive performance, the comparative assessment evidently ranks the N-HITS as the most correct and reliable setup in the base case. Consequently, N-HITS was chosen as the reference model to be utilized in the further metaheuristic optimization phase by the findings. The choice is made to make optimization efforts focused on the most promising architectural foundation that has been discovered in the process of conducting a baseline evaluation.

We use complementary polar-based visual analytics in order to achieve a full-fledged and visually easy-to-understand comparison of the predictive abilities of the investigated models. In contrast to traditional tabular displays, polar and radial plots permit multi-metric assessment at once without any loss of the relative preeminence hierarchy of competing methods. This is critical, especially in a multi-criteria performance evaluation, where error-based measures (MSE, RMSE, MAE, MAPE, SMAPE, RRMSE) need to be viewed together with the goodness-of-fit measures (R2 and NSE).

**Figure 11:**
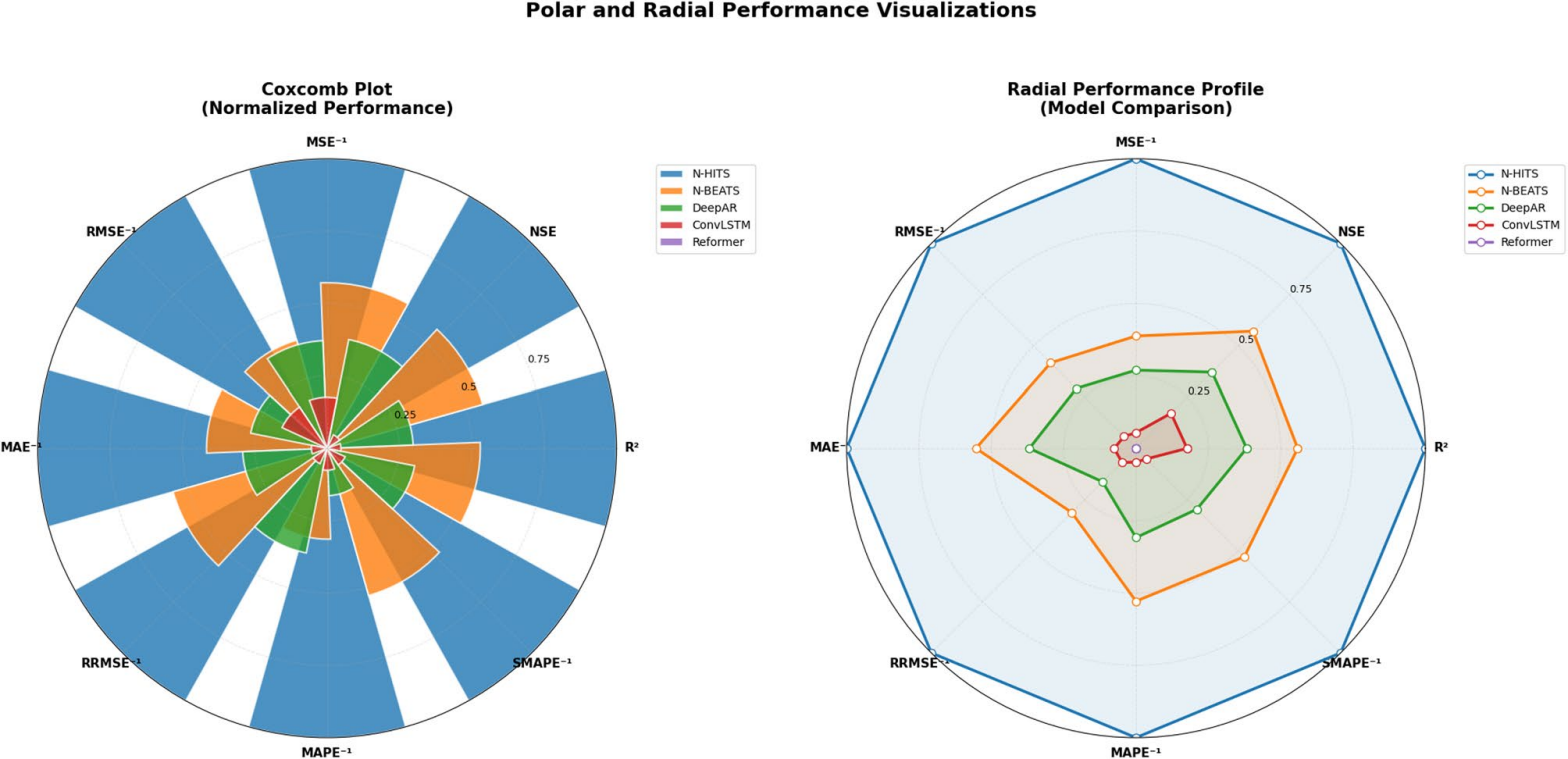
Polar and radial visualizations of normalized model performance across eight evaluation metrics.

Figure 11 displays two synchronized visualisations: a Coxcomb (polar bar) plot showing the magnitude on a normalised scale of the metric metrics and a radial performance profile so direct geometric comparison between models can be made. The metrics that are based on the error are all scaled negatively (indicated by $^{-1}$, as well) so that a homogenous approach where radial distance grows equally reflects good performance. This change allows for a fair visual comparison as the minimization and maximization criteria are put on the same scale. The Coxcomb representation shows the proportionality of each metric in the performance roster, whereas the radial profile shows the patterns of structural predominance across models. The combined visualizations present cohesive knowledge of the accuracy, strength, and overall model performance in all evaluation aspects.

**Figure 12:**
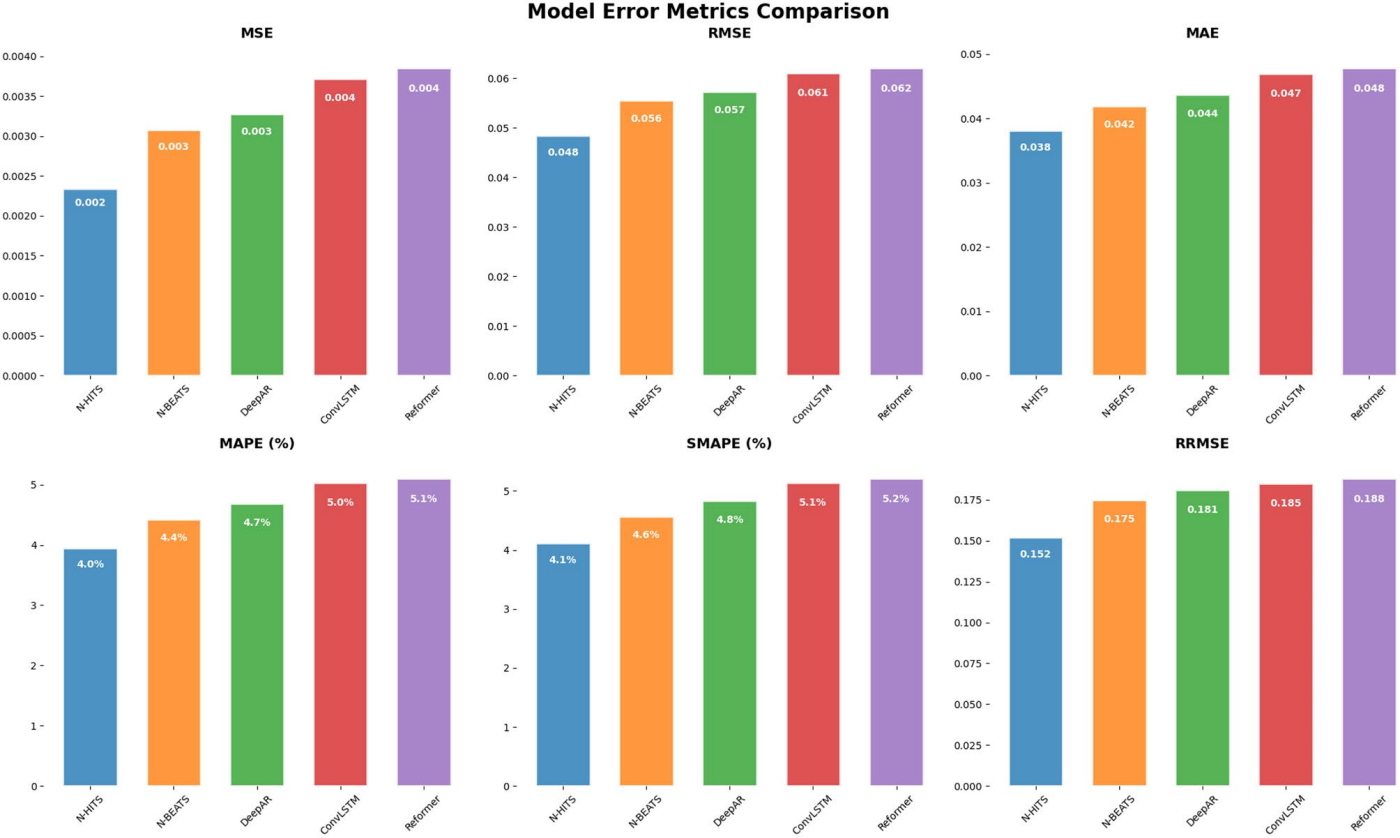
Bar chart comparison of key error metrics across competing predictive models. Lower values indicate better performance for all displayed metrics.

To analyze predictive performance rigorously, one should consider in some detail error-based indicators, which are used to measure how much there is a deviation between the observed and prediction value. Whereas the aggregate summaries give the general picture, the disaggregated metric specific-comparisons allow the model robustness, bias behavior, as well as sensitivity to large deviations to be interpreted in a more straightforward way. Precisely, the squared-error measures (e.g., MSE, RMSE) focus on the penalization of larger errors, whereas the absolute-error ones (e.g., MAE, MAPE) are less interpretable but are more useful as measures of average deviation. The symmetric SMAPE version suppresses scale effects, and RRMSE is a relative measure of the magnitude of observations. To visually compare the six commonly used error metrics by all of the models under test, a bar chart comparison of the six error metrics is provided in Fig. 12. This visualization enables direct comparison of cross-models in any given metric, and the performance trends may be constantly determined. Since the lower values denote a better predictive performance of all the presented figures, relative bar height can serve as a direct indicator of model efficiency. The figure thus contributes to the multi-metric visualizations used in the previous sections, packaging the actually observed error magnitudes and mode of consistency of performance in regard to evaluation measures.

### Optimized N-HITS performance

After the comparison at the baseline level, N-HITS was moved to the metaheuristic level of optimization since it was the architecture that outperformed the others at the pre-optimization stage. The forecasting outcomes after hyperparameter optimization with GLO and nine benchmark optimizers that are presented in Sect. 2.5.2 are provided here. In order to superimpose statistical strength, all optimizers were performed in various independent runs. The values reported are the mean performance of the predictive accuracy ± the standard deviation, hence being indicative of predictive accuracy and stability of convergence. The optimization results in terms of all key indicators are presented in Table 19. As indicated in Table 19, the introduction of the metaheuristic optimization improved the predictive accuracy of N-HITS in every evaluation measure in a valuable way. Among the tested algorithms, The lowest MSE (7.980e-05), smallest RMSE (0.00893), smallest MAE (0.00634), and lowest relative error (RRMSE = 0.0300) were attained by GLO + NHITS, which, at the same time, results in the largest value of R2 (0.9892) and NSE (0.9854). The given standard deviations show that GLO is very stable in terms of convergence in comparison with benchmark optimizers. Even though PSO and WAO realized significant results as well, their gesture values were always significantly higher than those received by GLO. Since GLO outperformed in the search hyperparameter space of N-HITS, the decreasing performance was recorded over GA, BA, BBO, DE, SFS, APO, and MVO.

**Table 19:** Performance of optimized N-HITS using metaheuristic algorithms.

Optimizer	MSE	RMSE	MAE	MAPE (%)	SMAPE (%)	$R^2$	NSE	RRMSE	MBE
GLO+N-HITS	7.980e-05 ± 7.980e-07	0.00893 ± 0.00004	0.00634 ± 0.00006	1.216 ± 0.005	1.263 ± 0.015	0.9892 ± 0.0049	0.9854 ± 0.0059	0.0300 ± 0.0003	1.106e-04 ± 1.327e-06
PSO+N-HITS	1.100e-04 ± 1.173e-06	0.01049 ± 0.00006	0.00749 ± 0.00008	1.310 ± 0.009	1.363 ± 0.018	0.9821 ± 0.0057	0.9798 ± 0.0064	0.0352 ± 0.0004	1.595e-04 ± 2.091e-06
WAO+N-HITS	1.300e-04 ± 1.473e-06	0.01140 ± 0.00006	0.00818 ± 0.00010	1.395 ± 0.014	1.441 ± 0.021	0.9784 ± 0.0064	0.9771 ± 0.0069	0.0383 ± 0.0004	1.909e-04 ± 2.715e-06
GA+N-HITS	1.500e-04 ± 1.800e-06	0.01225 ± 0.00007	0.00884 ± 0.00011	1.478 ± 0.019	1.523 ± 0.025	0.9741 ± 0.0071	0.9723 ± 0.0075	0.0411 ± 0.0005	2.108e-04 ± 3.232e-06
BA+N-HITS	1.700e-04 ± 2.153e-06	0.01304 ± 0.00008	0.00946 ± 0.00013	1.542 ± 0.024	1.593 ± 0.028	0.9706 ± 0.0079	0.9688 ± 0.0080	0.0438 ± 0.0006	2.206e-04 ± 3.628e-06
BBO+N-HITS	1.900e-04 ± 2.533e-06	0.01378 ± 0.00009	0.01005 ± 0.00015	1.608 ± 0.030	1.659 ± 0.032	0.9658 ± 0.0086	0.9634 ± 0.0085	0.0463 ± 0.0006	2.396e-04 ± 4.206e-06
DE+N-HITS	2.100e-04 ± 2.940e-06	0.01449 ± 0.00010	0.01063 ± 0.00016	1.655 ± 0.035	1.723 ± 0.036	0.9601 ± 0.0093	0.9578 ± 0.0089	0.0487 ± 0.0007	2.519e-04 ± 4.702e-06
SFS+N-HITS	2.300e-04 ± 3.373e-06	0.01517 ± 0.00011	0.01118 ± 0.00018	1.718 ± 0.042	1.787 ± 0.040	0.9547 ± 0.0100	0.9521 ± 0.0094	0.0509 ± 0.0007	2.593e-04 ± 5.128e-06
APO+N-HITS	2.500e-04 ± 3.833e-06	0.01581 ± 0.00012	0.01172 ± 0.00020	1.792 ± 0.049	1.858 ± 0.044	0.9483 ± 0.0106	0.9462 ± 0.0099	0.0531 ± 0.0008	2.729e-04 ± 5.701e-06
MVO+N-HITS	2.700e-04 ± 4.320e-06	0.01643 ± 0.00013	0.01224 ± 0.00022	1.845 ± 0.055	1.913 ± 0.048	0.9301 ± 0.0112	0.9284 ± 0.0102	0.0552 ± 0.0009	2.771e-04 ± 6.096e-06

These results indicate that metaheuristic hyperparameter optimization dramatically increases the prediction ability of N-HITS, and GLO offers the best exploration-exploitation ratio among all the optimizers considered. In addition to comparative accuracy assessment, the statistical interest in studying the distributional characteristics of the performance metrics of optimizers is sought. There are a lot of inferential procedures and parametric statistical tests on the assumption that the underlying metric distributions are approximated to be normal. Thus, a measure of the compliance of the measured metric values to the Gaussian distribution offers the added assurance of the strength and interpretability of comparative tests.

**Figure 13:**
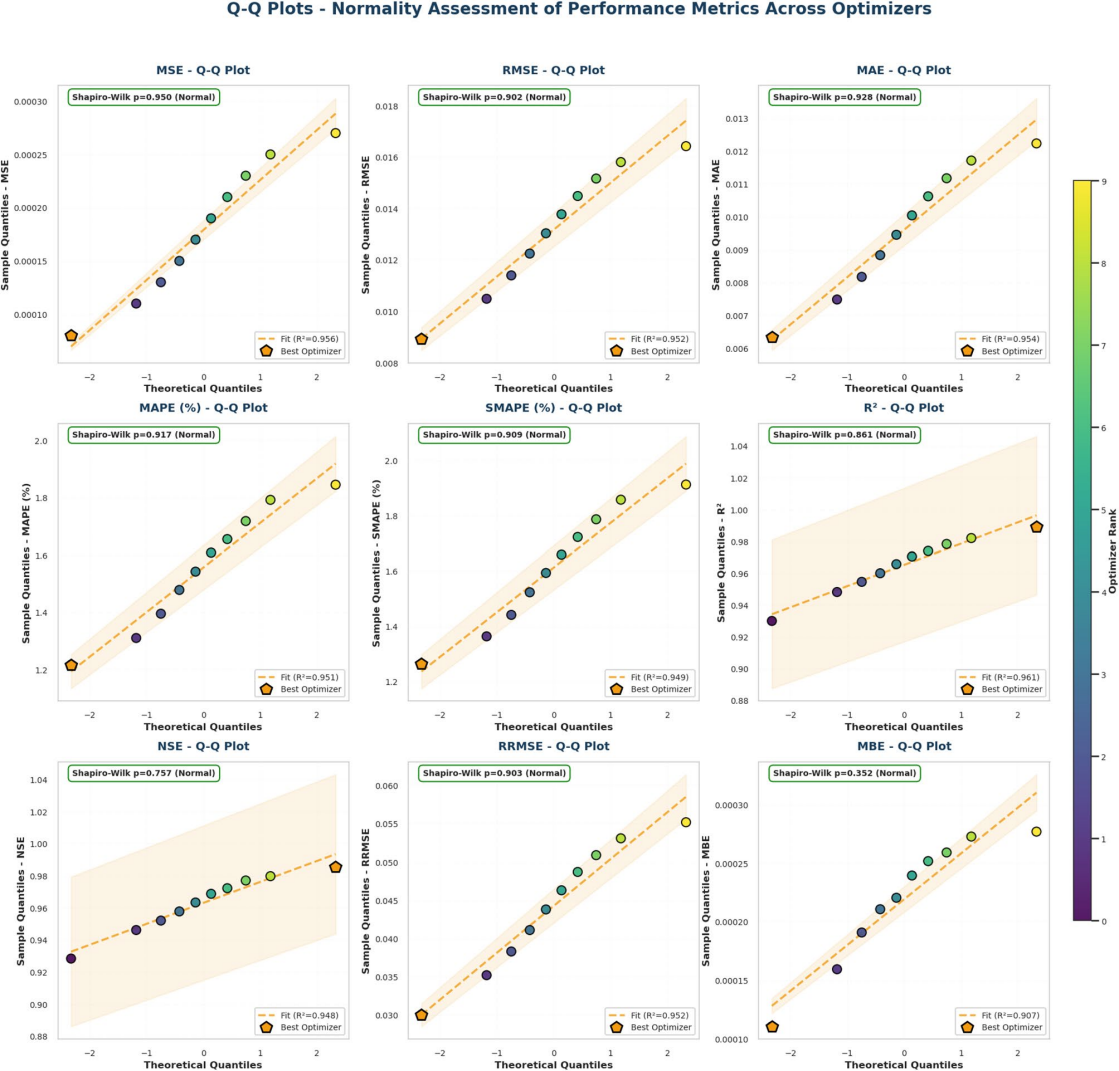
Q–Q plots assessing the normality of performance metrics across optimizers. The dashed line represents the linear fit to theoretical normal quantiles, and shaded regions indicate confidence intervals.

Figure 13 illustrates Q–Q (quantile-quantile) plots of nine metrics of evaluation, MSE, RMSE, MAE, MAPE, SMAPE, and RRMSE and the additional MBE. The plots of the ordered sample quantiles of the metric values against the normal distribution theoretical quantiles of each subplot are presented. The dashed regression curve represents the optimum linear fit, whereas the dark area represents the confidence bandage around the fitted relationship. Non-linearity would indicate non-normality, whereas an excellent fit is an indication of almost Gaussian behavior. The Shapiro–Wilk test is reported in each panel in order to supplement the visual inspection. The related *p*-values are a formal statistical test of normality, such that when any of the values is larger than standard significance levels, then there is no evidence against the assumption of normality. The graphical and statistical diagnostics provide an all-inclusive analysis of the distributional consistency of optimizers, enhancing the validity of any further performance analyses.

**Figure 14:**
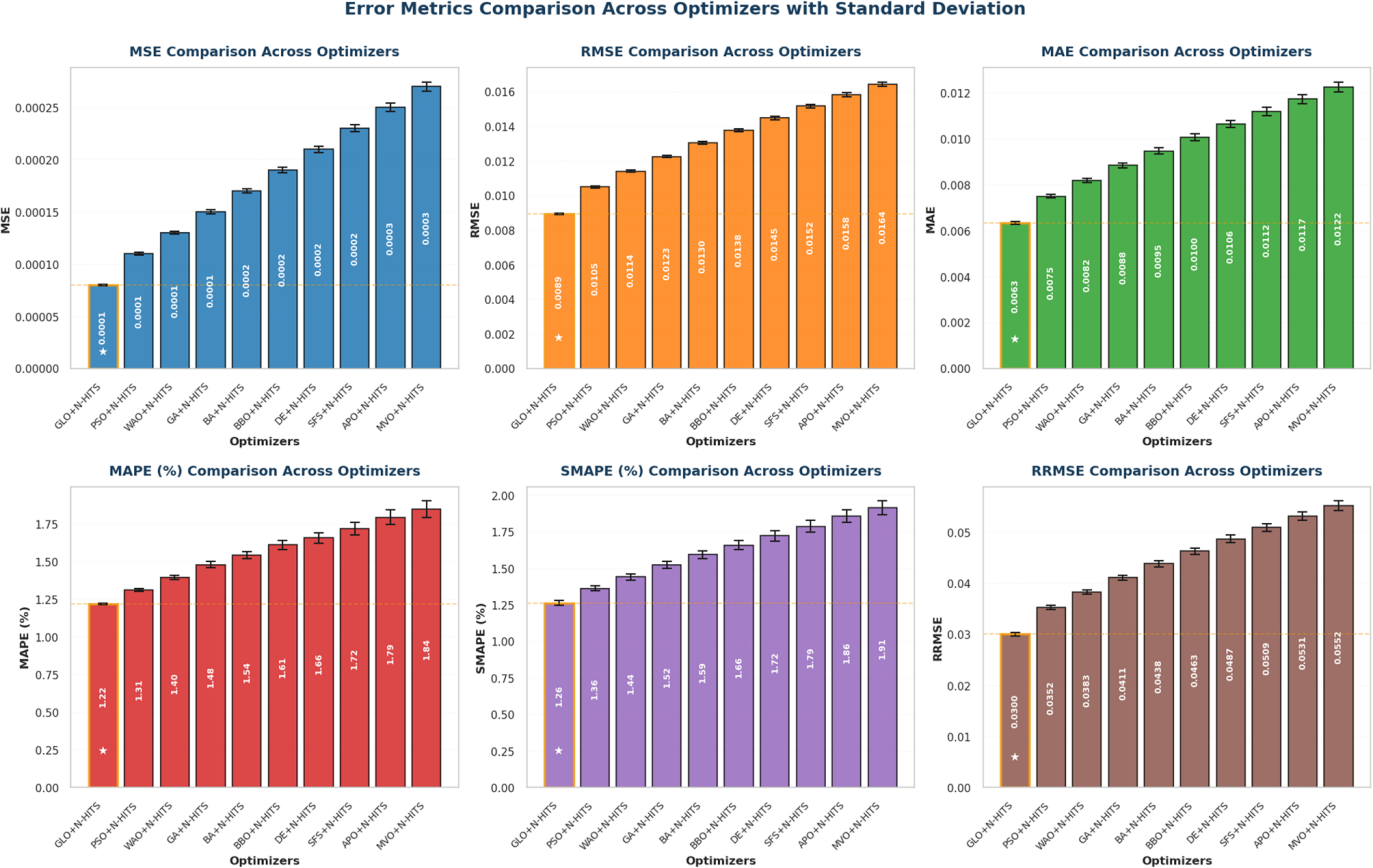
Comparison of error metrics across optimizers with standard deviation indicated by error bars. Lower values indicate better performance for all displayed metrics.

Although mean error values and their values are an estimate of central predictive performance may not correctly represent the stability and robustness of each optimizer. Consistency across repeated runs is as significant as absolute accuracy in the case of practical predictive process applications, particularly in nonlinear and nonstationary environments of practical forecasting. This is why the addition of dispersion measures, e.g., standard deviation, makes it possible to assess the reliability of optimizers in a more comprehensive way. Low variability shows a behavior of convergence stability and decreases with respect to the initial state or stochastic search processes. Figure 14 shows a comparison between six measures of error (MSE, RMSE, MAE, MAPE, SMAPE, and RRMSE) among optimizers investigated with standard deviation as error bars. The representation of the best-performing optimizer by a dashed line, referring horizontally to each of the subplots, indicates that the optimizer is successful, and the star is an additional indicator of its comparative success. The two representation options provide a chance to simultaneously assess the accuracy (mean metric value) and robustness (dispersion around the mean). The combination of central tendency and variability established in one framework can give a better understanding of the variability and stability of the optimization, the reliability of convergence and the consistency of generalization, as detailed in valve of the figure. This kind of analysis is needed not only to determine the best optimizer but also the most reliable one in the repeated experimental conditions.

**Figure 15:**
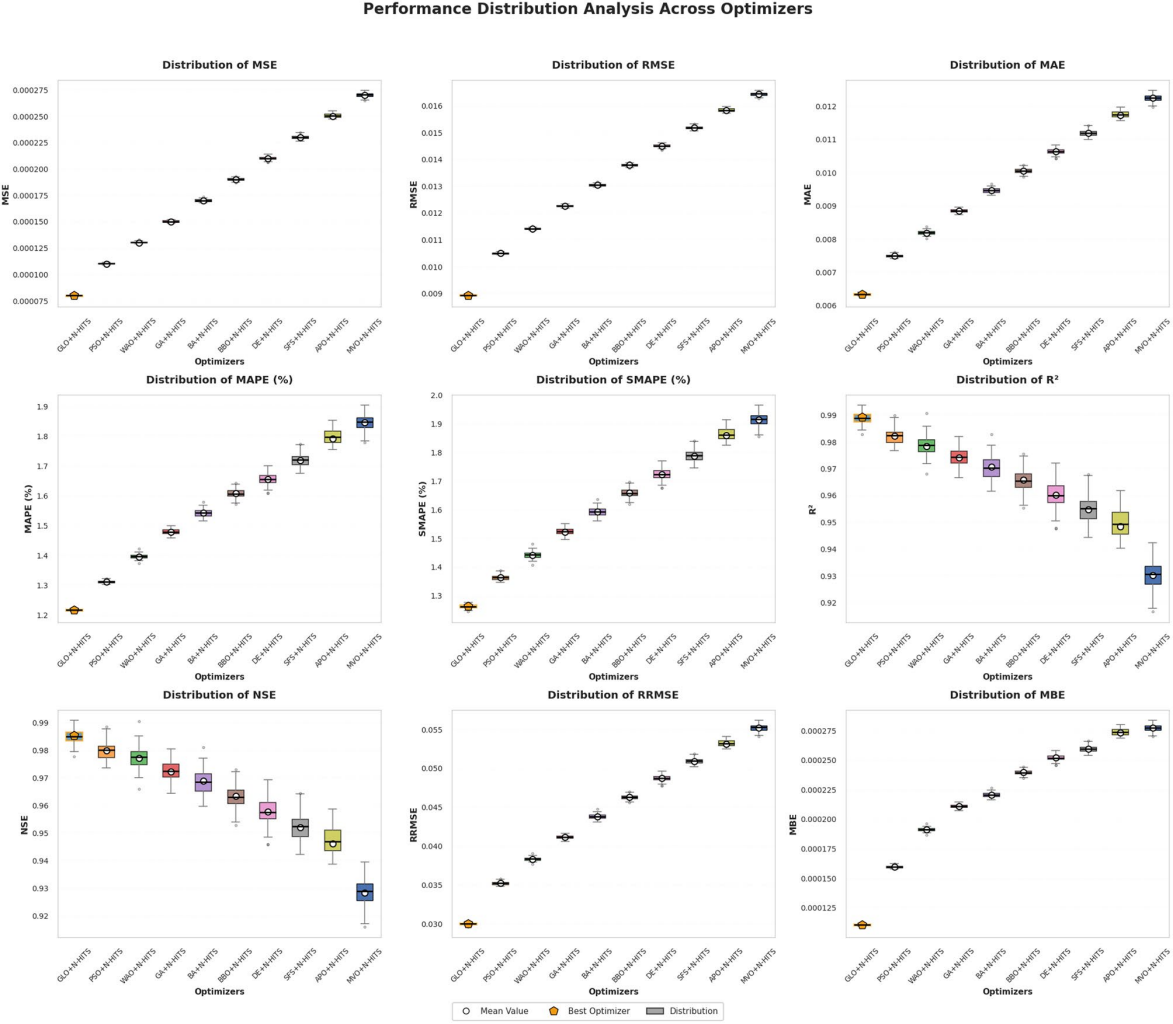
Boxplot-based distribution analysis of performance metrics across optimizers. Lower values indicate better performance for error-based metrics, while higher values indicate better performance for $R^2$ and NSE.

Mean values do not allow evaluation of predictive performance as much as is needed to reveal the facts about dispersion, skewness, and outliers. In stochastic optimization designs, repeating executions will repeatedly have a small fluctuation as they are randomly initialized and explored. This makes distributional analysis necessary in measuring convergence consistency, robustness and perturbation sensitivity. The analysis of full metric distributions can give more insight into the stability properties of each optimizer, other than central tendency measures. Figure 15 shows the boxplot-based distributions of nine performance measures, such as MSE, RMSE, MAE, MAPE, SMAPE, R2, NSE, RRMSE and MBE. The smaller the error-based measure, the better the performance is, whereas the larger the distribution of goodness-of-fit measures (R2 and NSE), the better. Both subplots present the median, interquartile range, dispersion, and possible outliers of optimizers. Mean value marker and best-optimizer indicator also help in comparative interpretation of each of the metrics. This wholesome distributional approach allows the determination of accuracy, variability, and reliability in a concurrent manner. The analysis of the central location and the dispersion and form of every metric distribution shows that the figure gives a solid base for determining optimizers that yield a high predictive accuracy and stable convergence strategy.

### Training and validation dynamics

**Figure 16:**
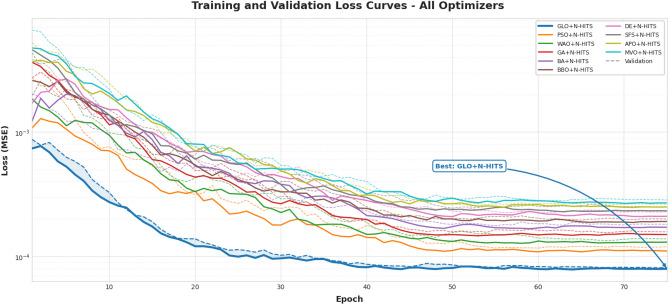
Training and validation loss curves across all optimizers. Solid lines represent training loss, while dashed lines indicate validation loss.

In order to compare the learning behavior and convergence properties of the proposed optimization strategies, we compare training and validation loss curves with the epoch. It is important to monitor these curves to evaluate the stability of optimization, the rate of convergence and whether overfitting is possible. Whereas training loss is used to measure the effectiveness of the model at minimizing the objective function on seen data, validation loss can be used to externally measure the generalization performance. When the two curves steadily decline without much deviation, this is a good indicator of consistent learning and good generalization. Figure 16 shows the training and validation loss curves of all optimizers in a single framework. This comparison on a global basis brings to the fore the speed of convergence in relation to stability tendencies and the end performance. The logarithmic scale of loss is used to better visualize the visualizations of convergence of differences in the later stages. It is worth noting that the optimizer with the best performance has a faster decay, a smaller asymptotic loss, as well as a small difference between the training curve and the validation curve, which shows less overfitting and better generalization performance.

**Figure 17:**
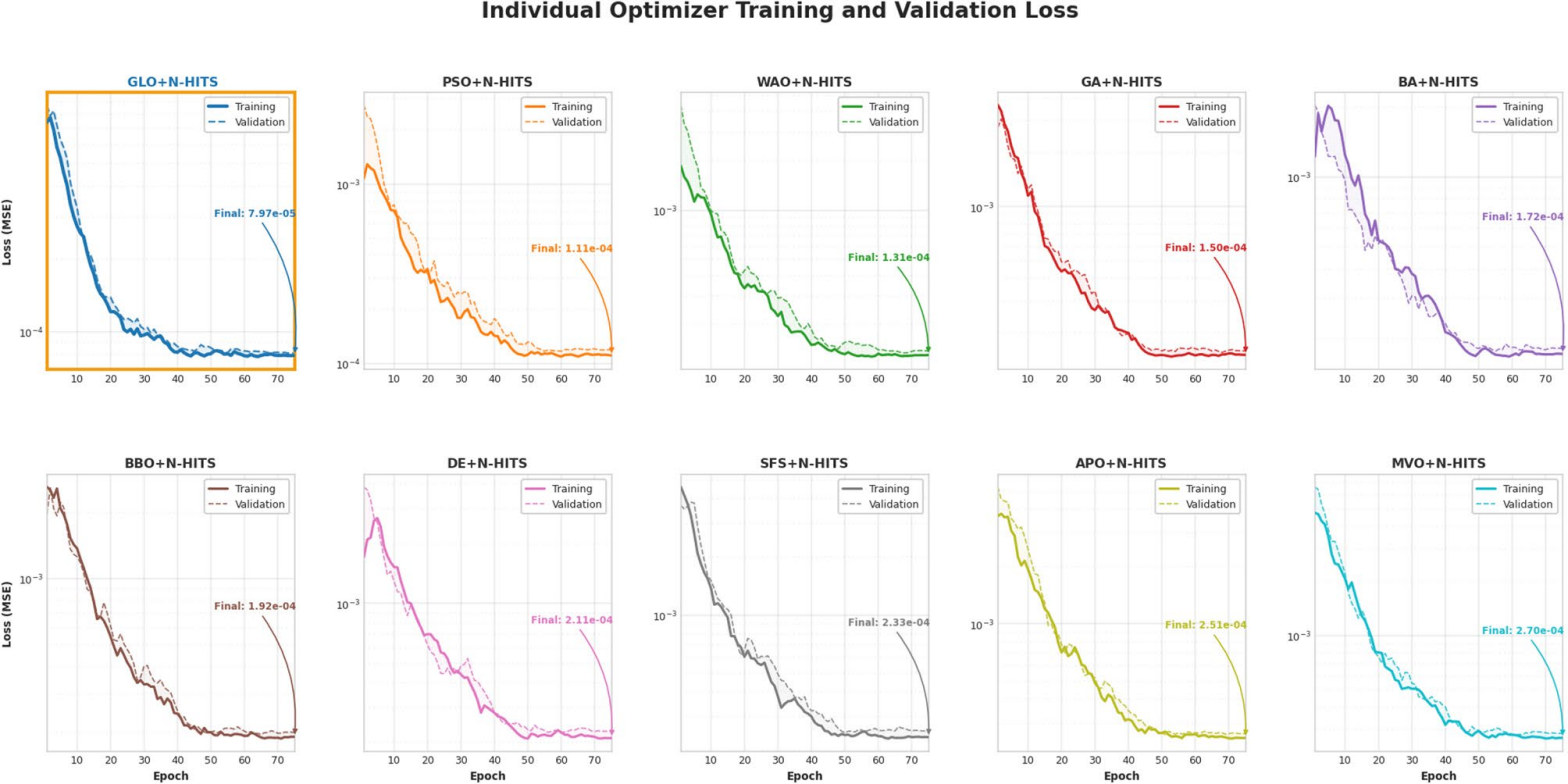
Individual training and validation loss curves for each optimizer, with annotated final loss values.

In order to more intimately examine convergence behavior, Fig. 17 displays the training and validation curves of each optimizer. Such a disaggregated representation permits a closer study of the level of convergence smoothness as well as oscillatory behavior and the ultimate levels of stabilization. The resulting value of loss for each optimizer is annotated to easily compare the results of the asymptotic performance of the optimizers. Using such an in-depth analysis, one can see the variance in efficiency in optimization better. Optimizers with high early convergence rates and flat plateauing rates indicate an optimizer’s good exploration-exploitation balance, where slower convergence can be indicative of weaker search dynamics. The comparative evaluation of the two characters thus avails an overall realization of optimization steadiness, overallization faithful and end predictability.

### Computational resources

Besides predictive accuracy, an extensive analysis of computational efficiency was done to evaluate the realistic feasibility of each optimizer used in combination with the N-HITS architecture. The fact that metaheuristic optimization is based on population-based search mechanisms that cannot be executed in one iteration makes computational cost a key consideration, especially when treating the seismic forecasting system in real-time or with large scale. Thus, all combinations of optimizers and models were systematically trained, and their time, memory usage, CPU usage, and GPU usage were logged. Training time (s) refers to the total time in seconds to be optimized to the last parameter configuration. Memory (MB) is used to measure the highest RAM usage at any time during execution. CPU utilization (%) is defined as the average workload per processor at optimization, whereas GPU utilization (%) is a measure of parallel computation acceleration utilized by each approach. All values are presented as mean which are indicated between two or more experimental runs in order to attain a good statistical reliability. Comparison of the computational resources is given in detail in Table 20.

**Table 20:** Computational resource consumption of optimized N-HITS.

Optimizer	Training time (s)	Memory (MB)	CPU (%)	GPU (%)
GLO+N-HITS	48.3 ± 1.82	312.4 ± 3.82	34.2 ± 1.94	92.4 ± 0.74
PSO+N-HITS	63.7 ± 2.41	487.3 ± 8.74	61.7 ± 3.82	84.6 ± 1.23
WAO+N-HITS	67.4 ± 2.58	412.8 ± 6.93	54.3 ± 3.21	86.2 ± 1.05
GA+N-HITS	71.2 ± 2.94	463.5 ± 7.85	67.2 ± 4.03	83.1 ± 1.34
BA+N-HITS	73.8 ± 3.05	398.2 ± 6.41	58.9 ± 3.58	85.4 ± 1.18
BBO+N-HITS	75.6 ± 2.87	521.4 ± 9.23	71.4 ± 4.47	81.7 ± 1.47
DE+N-HITS	79.3 ± 3.45	476.9 ± 8.15	65.8 ± 4.12	82.8 ± 1.29
SFS+N-HITS	84.7 ± 3.76	503.7 ± 8.97	74.3 ± 4.89	80.3 ± 1.52
APO+N-HITS	91.2 ± 4.23	541.2 ± 9.84	79.6 ± 5.23	78.9 ± 1.63
MVO+N-HITS	108.5 ± 5.14	558.8 ± 10.63	83.2 ± 5.61	76.4 ± 1.71

According to Table 20, GLO+N-HITS has the shortest training time (48.3 s) and the lowest memory footprint (312.4 MB), as well as the least CPU utilization (34.2%) and highest GPU utilization (92.4%). It shows high computational efficiency and good adoption of parallel processing resources. Conversely, MVO+N-HITS has the longest training interval (108.5 s), the highest memory requirement (558.8 MB), as well as, it has more CPU dependency and fewer usages of the GPUs. The computational profiles of other optimizers, such as PSO, WAO, GA, BA, BBO, DE, SFS, and APO, are intermediate as they indicate different ratios between the complexity of exploration and convergence dynamics.

A quantitative comparison of computational efficiency further confirms the advantage of the proposed GLO+N-HITS configuration. As reported in Table 20, GLO+N-HITS achieved the shortest training time (48.3 ± 1.82 s), the lowest memory consumption (312.4 ± 3.82 MB), and the lowest CPU utilization (34.2 ± 1.94%) among all compared optimizers, while also showing the highest GPU utilization (92.4 ± 0.74%). In contrast, competing methods such as PSO, GA, SFS, APO, and MVO required substantially higher training time and memory resources. These quantitative differences demonstrate that the proposed framework offers not only higher predictive accuracy, but also a more favorable accuracy–efficiency trade-off for practical deployment.

On the whole, the computational analysis shows that GLO not only increases predictive accuracy, but it also has a much better efficiency performance trade-off that makes GLO especially well-adapted to scalable seismic forecasting systems. Along with the predictive performance and convergence stability, in order to deploy the optimization strategy practically, it is necessary to pay attention to the efficiency of the computations. Scalability, reproducibility and real-world applicability directly depend on resource consumption, especially in large-scale forecasting or in high-frequency retraining cases. The overall computation throughput is dictated by the training time, hardware feasibility is dictated by the memory footprint, and processing intensity and parallelization efficiency are represented by the utilization of the CPU/GPU units. Thus, performance quality and computational cost should be evaluated in a complete manner.

**Figure 18:**
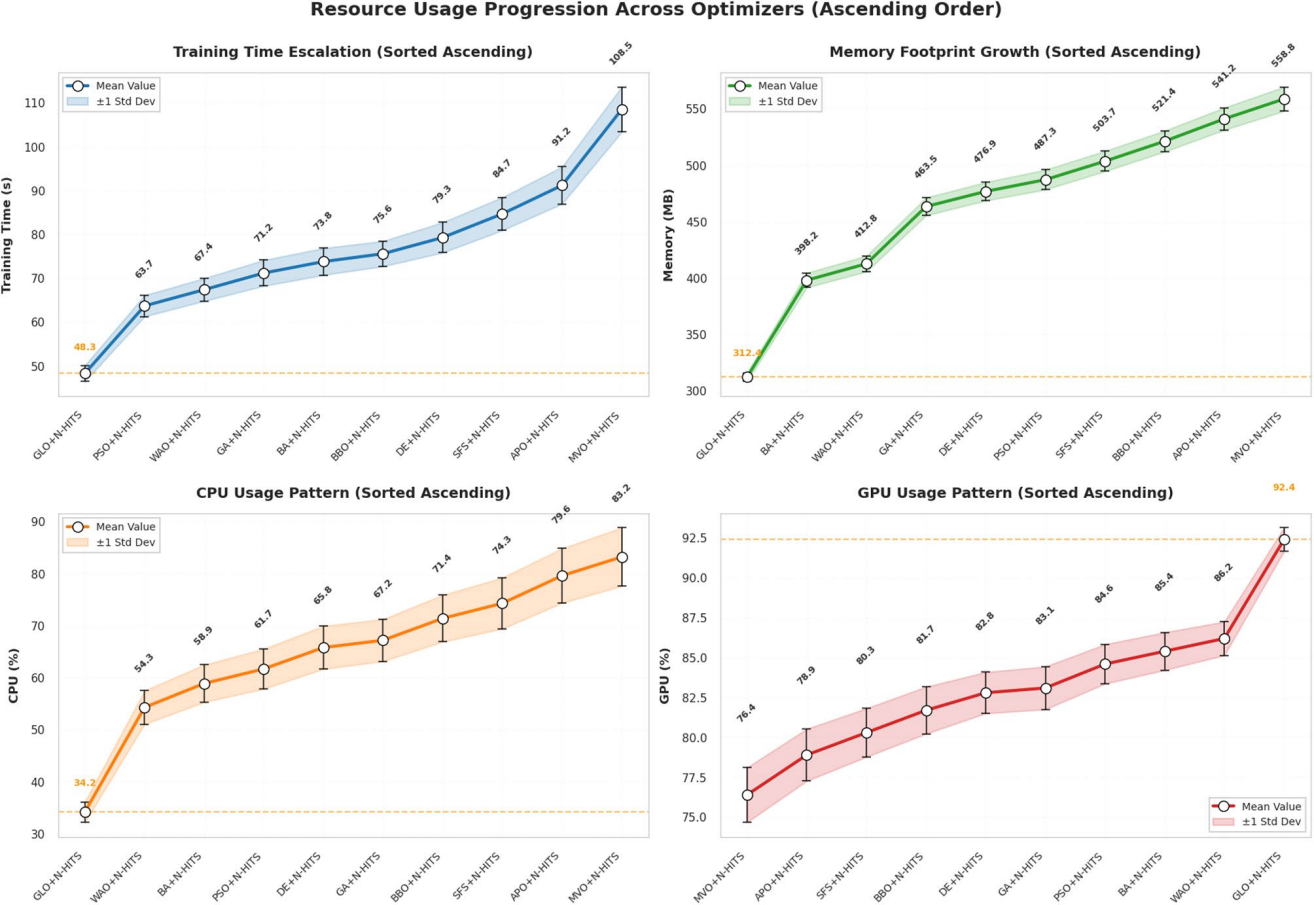
Progression of computational resource usage across optimizers (sorted in ascending order). Mean values and standard deviations are shown for training time, memory footprint, CPU usage, and GPU usage.

Figure 18 will show the progression of the resource usage in each of the optimizers in an ascending order. The analysis of four complementary indicators is conducted: training time (in seconds), memory consumption (in MB), CPU utilization (%) and GPU utilization (%). The mean and the standard deviation of the report make each subplot indicate variability among the repeated runs. Sorting the optimizers in increasing order increases the interpretation induced by showing the relative scale behavior of the demand for the computations. The most computationally efficient configuration within each category of resources is indicated by the dashed line of reference.

This combined exploration allows for locating the optimizers leading to good trade-offs in predictive performance and in computational expense. Optimizers nearer to the bottom of the progression curves are more efficient and scaleable whereas those nearer to the top require higher hardware and time. This type of assessment is critical to the choice of optimization approaches that can both be accurate and operationally feasible in practical implementations.

In addition to predictive behavior and convergence rate, the convergent behavior as well as the predictability behavior of the optimization algorithms are greatly reliant on their efficiency in computation. In real-life forecasting systems, scalability and feasibility of deployment can be hindered by a lot of training time, big memory use, or high processor use. Thus, to augment performance-based comparisons, a systematic assessment of the needs of computational resources is necessary to find optimizers to provide a good combination of accuracy and efficiency.

**Figure 19:**
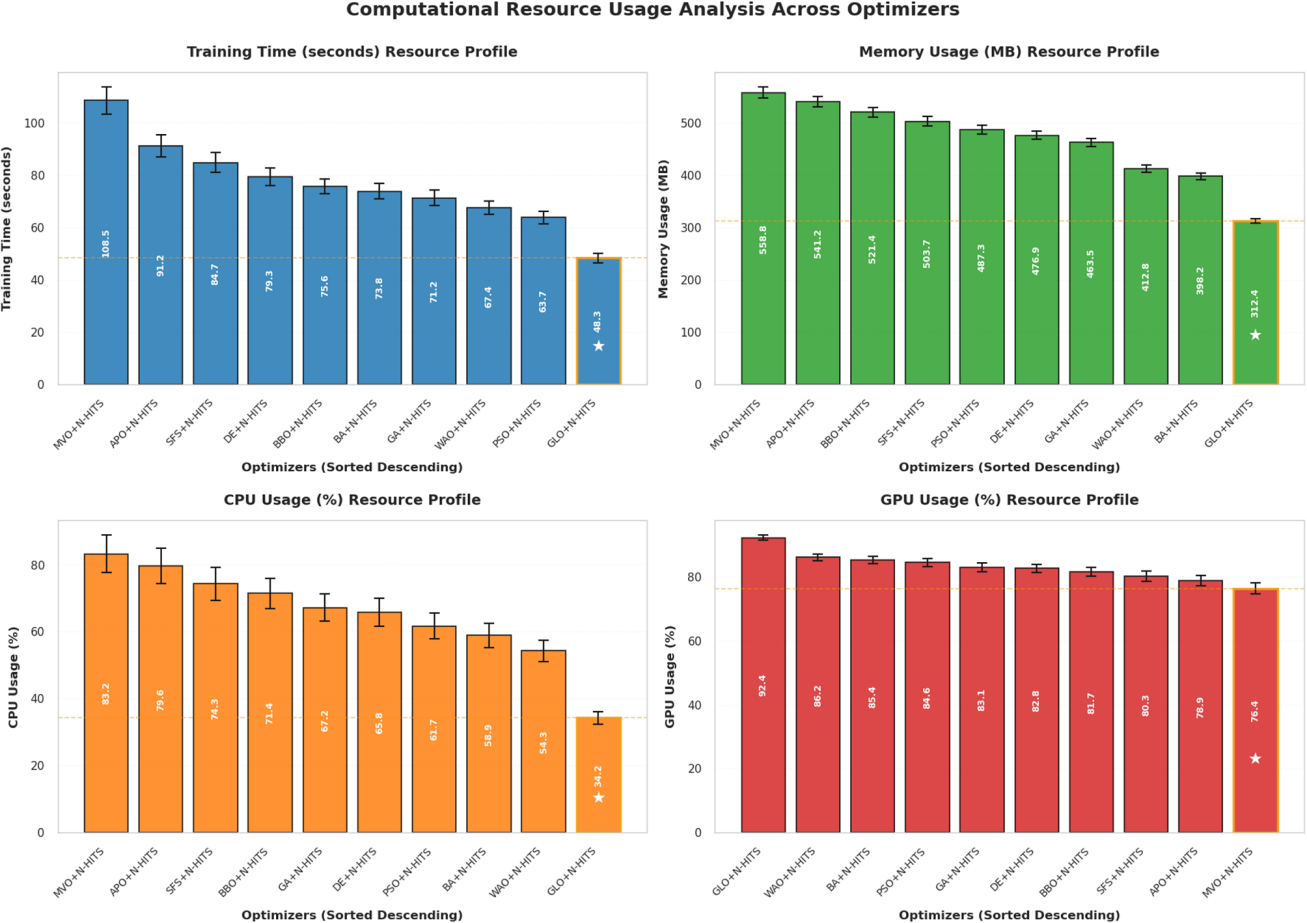
Computational resource usage profiles across optimizers. Metrics include training time, memory usage, CPU utilization, and GPU utilization, with optimizers sorted in descending order within each category.

Figure 19 shows a detailed resource profile of all optimizers in terms of training time (in seconds), memory (MB) and CPU utilization (%) or GPU utilization (%). The optimizers are arranged in a descending order within each subplot, which will explicitly show the relative amount of computation cost of each method. Error bars indicate the standard deviation and show the variation between experimental passes, giving an understanding of the stability of the resources. The direct-horizontal reference line shows the best possible configuration of every category, and the bar of color makes emphasis on the optimizer with the least resource usage.

Such an organized comparison will allow identifying optimizers, which will ensure the preservation of predictive performance at the competitive level and the suppression of the computational burden. This analysis is especially critical in the deployment of models in low-resource settings or in the use of complex systems of operations, where resource considerations may sometimes be just as important as the forecasting accuracy.

## Discussion

The empirical results demonstrate that forecasting performance is governed not only by model complexity but by the alignment between model structure, data characteristics, and hyperparameter optimization. The Canadian earthquake series exhibits non-stationarity, temporal clustering, and multi-scale variability, requiring models capable of capturing these properties while maintaining stable optimization.

The superior performance of N-HITS can be attributed to its hierarchical decomposition of temporal structure. Unlike sequential models such as DeepAR or attention-based models like Reformer, which may struggle to balance long-term trends and short-term fluctuations, N-HITS separates low- and high-frequency components, improving representation efficiency and convergence stability. This makes it particularly suitable for seismic series characterized by background activity and intermittent clustering.

From a seismological perspective, this improvement suggests that the optimized N-HITS–GLO framework is better able to represent important catalog-scale seismic behaviors rather than only reducing numerical error. The lower MSE indicates improved tracking of short-term fluctuations in seismic activity, which may correspond to burst-like event sequences such as aftershock clustering or localized seismic swarms. The higher $R^2$ indicates that the model captures a larger proportion of the temporal variability in the earthquake catalog, including the dominance of low-to-moderate magnitude events and the intermittent occurrence of larger events reflected in magnitude–frequency behavior. In this sense, the hierarchical structure of N-HITS is physically meaningful at the catalog level because it can distinguish rapid local variations from slower background trends, which is consistent with the coexistence of short-term clustering and longer-term stress accumulation–release patterns in seismic systems.

In contrast, sequential recurrent architectures may become sensitive to long-range temporal dependencies and accumulated prediction errors, particularly under highly variable seismic activity patterns. Similarly, attention-based models can require substantially larger and more stable datasets to reliably capture long-term dependencies without overfitting. The observed performance differences therefore suggest that model effectiveness is strongly influenced by the non-stationary, burst-like, and multi-scale characteristics of seismic catalog data, rather than by architectural complexity alone.

From a seismological perspective, the results indicate that earthquake magnitude evolution contains structured temporal patterns rather than purely random behavior. The ability of N-HITS to capture multi-scale dynamics is consistent with stress accumulation and release processes acting across different temporal scales. Therefore, the quantitative gains should be interpreted as evidence that the proposed model more effectively represents statistical signatures of seismic catalog dynamics, including clustering, magnitude-distribution imbalance, and multi-scale temporal variability. These gains do not imply deterministic earthquake predictability, but they indicate improved modeling of the temporal organization present in historical seismicity records.

Nevertheless, the interpretability results should be understood primarily as statistical explanations of learned data patterns rather than direct evidence of physical earthquake-generation mechanisms. Although certain temporal behaviors may appear consistent with known seismological processes, the SHAP-based analysis and feature-importance estimates only reflect correlations learned from historical catalog data. Consequently, the identified relationships should not be interpreted as causal geophysical drivers without additional physically informed validation.

Hyperparameter optimization further plays a critical role. The results show that performance improvements are not solely due to architecture, but also to effective parameter tuning. The Gray Langurs Optimizer (GLO) achieved a strong balance between exploration and exploitation, reducing premature convergence and improving stability. This highlights that optimization is essential for capturing the nonlinear and heterogeneous nature of seismic data.

The seismological relevance of the GLO-based improvement is related to the sensitivity of seismic time-series models to scale selection and regularization. For catalog data, an inappropriate lookback window may miss delayed aftershock activity or longer background-rate variations, while insufficient regularization may overfit rare high-magnitude events or localized event bursts. By optimizing the temporal window, architectural depth, learning rate, dropout, and related training parameters, GLO helps the N-HITS model better balance short-term clustered behavior with longer-term seismic-rate variability. Thus, the improvement in $R^2$ and MSE reflects not only better numerical fitting but also a more stable representation of the multi-scale structure of the earthquake catalog.

Although GLO achieved the best performance among the tested optimization strategies, the observed gains should be interpreted with caution. Because all metaheuristic methods perform some form of hyperparameter search, part of the improvement may reflect the general benefit of systematic tuning rather than the exclusive effect of GLO itself. Therefore, the results demonstrate that GLO is an effective optimizer within the adopted experimental setting, but they do not conclusively isolate its contribution from broader hyperparameter optimization effects. Future controlled ablation studies using identical search budgets, randomized search baselines, and repeated independent runs are needed to further quantify the specific contribution of GLO.

The contribution of this study lies not in proposing a single superior model, but in providing a unified benchmarking framework that systematically evaluates multiple architectures and optimization strategies under consistent conditions. This enables clearer interpretation of architecture–optimizer interactions and avoids biases from inconsistent preprocessing or tuning.

From a practical perspective, the framework improves not only predictive accuracy but also stability and reproducibility, which are important for future seismic monitoring applications. Nevertheless, its outputs should be interpreted cautiously as statistical trend estimates rather than operational earthquake predictions.

Overall, the results confirm that effective data-driven seismic activity prediction in catalog-based data depends on the joint optimization of representation and search strategy. N-HITS provides a strong multi-scale representation, while GLO enhances optimization stability, together forming a promising and reproducible framework for seismic time-series trend modeling.

## Limitations of the study

Although the proposed framework achieved strong predictive performance, several limitations should be acknowledged. First, the experiments are based mainly on a single national seismic catalog, namely the Canadian earthquake dataset. While this catalog provides broad temporal coverage and diverse seismic observations, it does not fully represent the variability of global tectonic environments. Therefore, the generalizability of the proposed framework across different seismic regimes, fault systems, and catalog characteristics requires further investigation.

Second, the framework relies exclusively on historical catalog-based variables and does not explicitly incorporate physical or geophysical drivers such as tectonic stress evolution, fault mechanics, crustal deformation, geodetic measurements, ground-motion parameters, or geochemical and ionospheric precursors. Consequently, the model outputs should be interpreted as data-driven statistical estimates of seismic activity trends rather than physically based earthquake predictions.

Third, although supplementary experiments on an independent dataset demonstrated broadly consistent performance trends under the adopted experimental setting, the current evaluation remains limited in terms of regional diversity and tectonic variability. As a result, the scalability and robustness of the framework across substantially different seismic zones cannot yet be conclusively established. Additional large-scale cross-regional validation is necessary before broader generalization claims can be made.

Fourth, the current framework does not include explicit uncertainty quantification. The proposed models generate deterministic point predictions without estimating predictive confidence intervals, probabilistic event distributions, or uncertainty bounds associated with the forecasts. Given the inherently uncertain, nonlinear, and non-stationary nature of seismic processes, future work should incorporate uncertainty-aware and probabilistic forecasting strategies.

Fifth, the evaluation protocol is based on a single chronological train–validation–test split. Although this approach preserves temporal consistency and prevents information leakage, the reported performance may still depend on the selected temporal partition. More robust validation strategies, such as rolling-origin or walk-forward validation, should be considered in future studies to better assess model stability under evolving temporal conditions.

Finally, the observed superiority of GLO should be interpreted within the adopted experimental design. Since all metaheuristic algorithms perform some form of hyperparameter search, part of the performance gain may reflect the general benefit of systematic optimization rather than the exclusive contribution of GLO. Future controlled ablation studies using identical search budgets, randomized-search baselines, and repeated independent runs are needed to isolate the specific effect of the optimizer more rigorously.

Accordingly, the proposed framework should be viewed primarily as a methodological benchmarking and statistical time-series modeling approach for seismic activity trend estimation, rather than as a direct operational earthquake prediction or early-warning system. Future research may strengthen its applicability by integrating catalog-based deep learning with physically informed geophysical models, probabilistic forecasting, and broader multi-region validation.

## Figures and Tables

**Figure 20:**
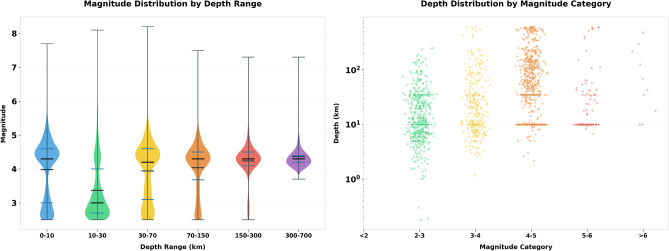
Left: Violin plots illustrating magnitude distributions across focal depth ranges. Right: Depth distributions across magnitude categories (logarithmic depth scale). Central markers denote medians, and distribution width reflects density.

**Figure 21:**
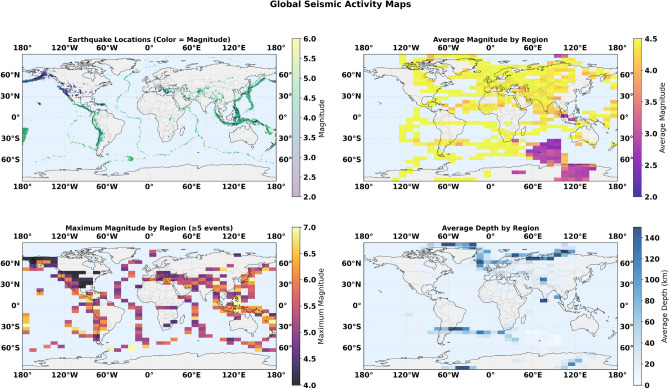
Global seismic activity maps. Top-left: Earthquake epicenters colored by magnitude. Top-right: Spatial distribution of average magnitude by region. Bottom-left: Maximum recorded magnitude per region (minimum five events per cell). Bottom-right: Average focal depth by region.

**Figure 22:**
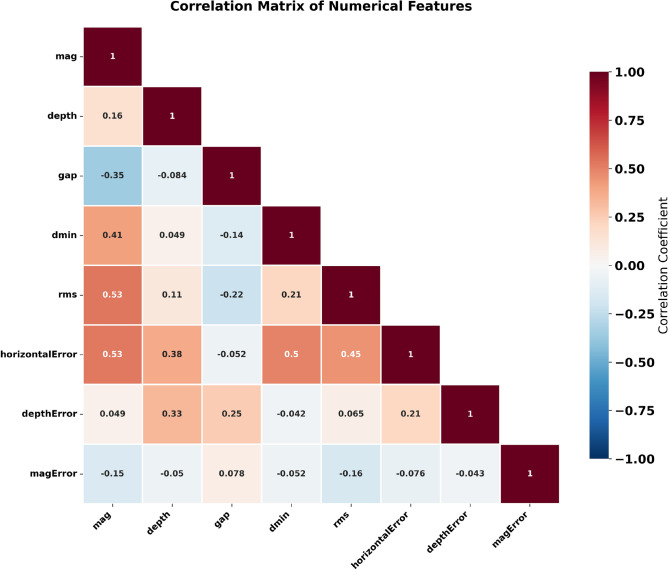
Correlation matrix of the primary numerical features in the earthquake dataset. Colors represent the magnitude and direction of Pearson correlation coefficients between variables, highlighting relationships among seismic attributes such as magnitude, depth, signal properties, and measurement errors.

**Figure 23:**
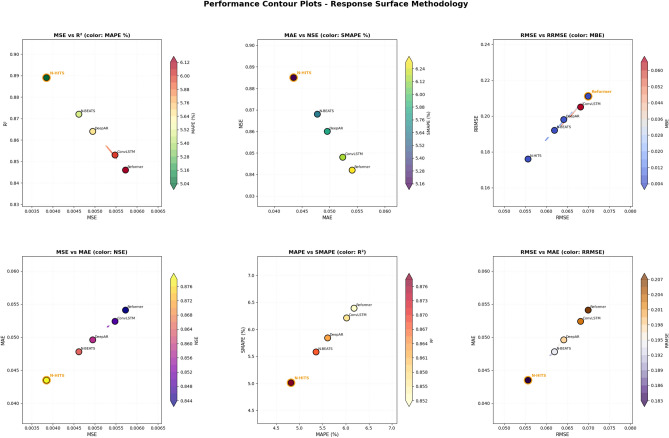
Performance contour plots using a response surface methodology framework. Each subplot illustrates pairwise metric relationships with color encoding representing an additional performance dimension.

**Figure 24:**
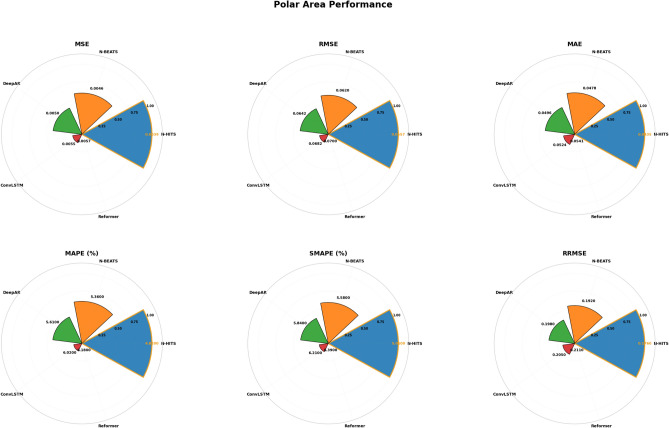
Polar area performance comparison across forecasting models for six evaluation metrics. Radial length represents normalized performance magnitude, and annotated values indicate exact metric scores.

**Figure 25:**
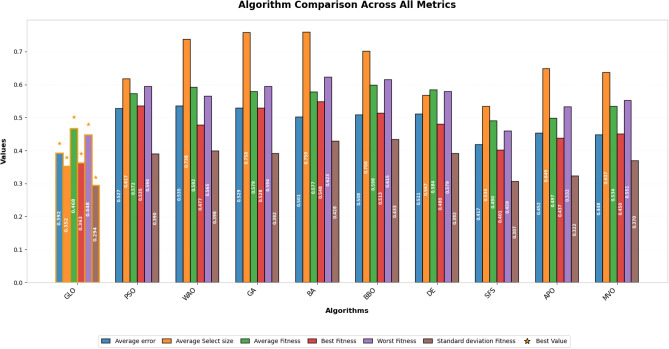
Comprehensive comparison of optimization algorithms across multiple performance metrics, including error, fitness statistics, and population variability measures.

**Figure 26:**
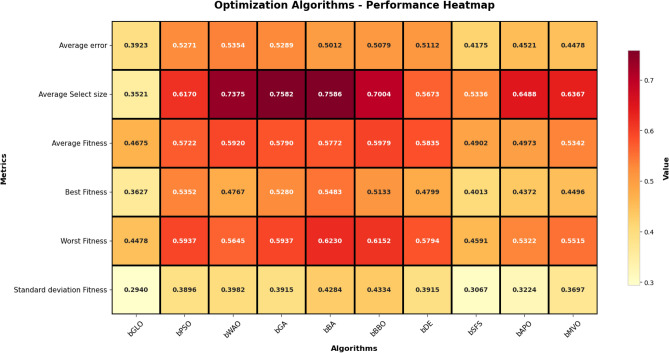
Heatmap comparison of optimization algorithms across multiple performance metrics. Color intensity represents metric magnitude, enabling cross-algorithm and cross-metric evaluation.

**Figure 27:**
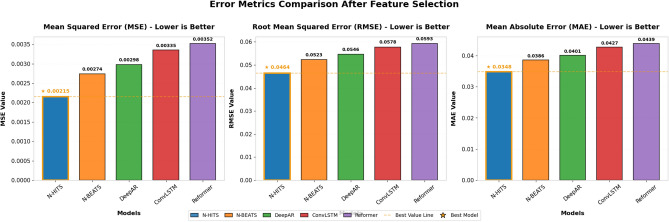
Comparison of primary regression error metrics after feature selection. Lower values indicate improved predictive accuracy. The best-performing model is highlighted in each subplot.

**Figure 28:**
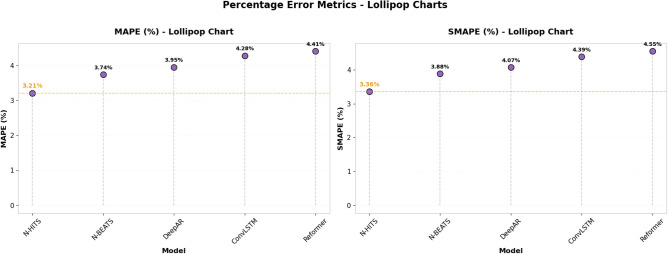
Comparison of percentage-based error metrics using lollipop charts. Lower MAPE and SMAPE values indicate improved proportional forecasting accuracy.

**Figure 29:**
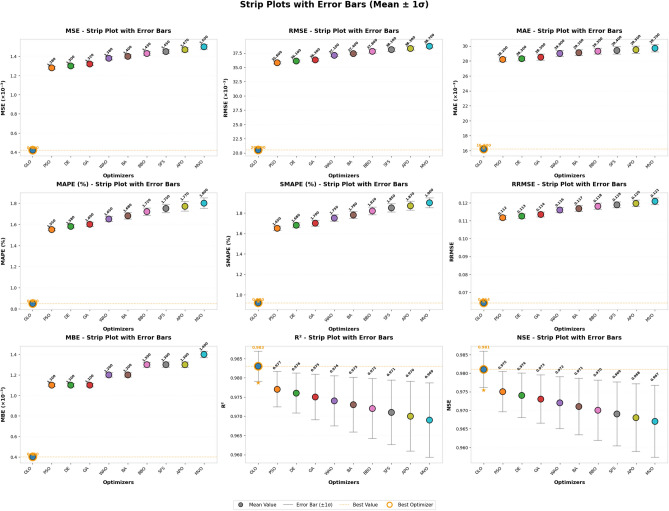
Strip plots with error bars (mean $\pm 1\sigma$) illustrating performance variability across optimization algorithms for multiple evaluation metrics.

**Figure 30:**
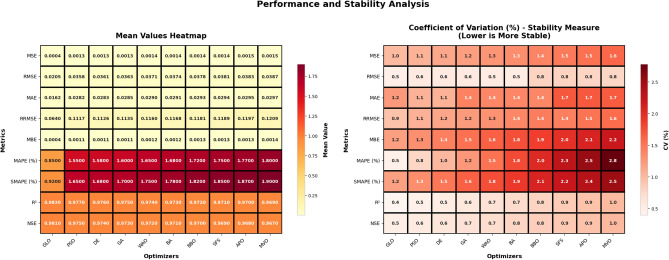
Performance and stability analysis of optimization algorithms. The left heatmap shows mean metric values, while the right heatmap presents the coefficient of variation (CV%) as a stability measure (lower indicates greater robustness).

## Data Availability

The data used in this study are publicly available at: https://www.kaggle.com/datasets/mshabrawy/earthquakes-in-canada-2010-2019.

## Appendix A: Supplementary experiments on an additional dataset

This appendix presents supplementary experiments conducted on an additional dataset to further examine the generalization and robustness of the proposed -GLO optimization framework beyond the primary dataset discussed in the main text.

### Dataset description

An earthquake is a natural occurrence that erupts when the tectonic stress accumulated is suddenly relieved through a fault plane, leading to the displacement of two blocks of the Earth’s crust. The rupture starts at a subsurface point called the hypocenter (or focus), and the point right on the Earth’s surface is called the epicenter. The seismic wave spreading that occurs due to this rupture causes ground motion, the intensity of which depends on the magnitude of earthquakes, the depth of the focal point, and the geological characteristics of the locality. The dataset used in the present research was received at the United States Geological Survey (USGS) Earthquake Hazards Program, which is an organized and systematic monitoring, recording, and validation of the seismic activity of the world.

The data will cover the entire set of recordings on the earthquake events within the period between 01 January 2021 and 31 December 2021. It includes over 28,000 observations and 18 attributes, which are temporal, spatial, and seismological attributes of each event. The dataset is not restricted by any predetermined analytical goal, which gives it flexibility in defining research questions, including: magnitude prediction, spatiotemporal clustering, seismic hazard assessment or data-quality evaluation. Table 21 summarizes the main features used in this research.

**Table 21:** Description of the main dataset features.

Feature	Description
Time	Timestamp indicating the exact occurrence of the earthquake event, enabling temporal analysis and time-series modeling
Latitude	Geographic coordinate specifying the north–south position of the earthquake epicenter
Longitude	Geographic coordinate specifying the east–west position of the earthquake epicenter, used together with latitude for spatial mapping and clustering
Depth	Depth (in kilometers) at which rupture initiates. It may be referenced relative to the WGS84 geoid, mean sea level, or station elevation averages. Depth significantly influences surface shaking intensity
mag	Official magnitude reported for the event. Magnitude quantifies the energy released at the source and is measured using logarithmic scales
magType	Method used to calculate magnitude (e.g., Md, Ml, Ms, Mw, Mb, Me, Mi, MLg), depending on waveform characteristics and frequency range
Gap	Largest azimuthal gap (in degrees) between adjacent seismic stations. Smaller values generally indicate better reliability of the horizontal location estimate
dmin	Horizontal distance from the epicenter to the nearest seismic station (in degrees; $1^\circ \approx 111.2$ km). Smaller values typically improve depth estimation accuracy
rms	Root-mean-square (RMS) travel-time residual (in seconds), measuring the discrepancy between observed and predicted wave arrival times. Lower values indicate better model fit
id	Unique identifier assigned to each seismic event for traceability within the catalog

On the whole, the dataset represents a multivariate picture of seismic activity in the world in 2021. Seismic updates its depth and the magnitude measures with spatial coordinates, which combine with the quality-control measures, provide an appropriate basis for the future application of advanced statistical analysis, machine learning, territory adjacent forecasts, and hazard models.

### Data preprocessing

Preprocessing of data is a very important procedure to guarantee reliability, robustness, and reproducibility of the later analytical and modeling methods. The preprocessing series applied in this research is missing value examination, information cleaning, imputation, time transformation, feature engineering and time-series modeling preparation. There was a complete missing value analysis of all the features of the data. The number of missing values and the corresponding percentages against the overall number of records of each column were calculated. The findings were ranked in order of decreasing values to recognize variables that had the largest number of missing data. The step was necessary to make sure that the data completeness and possible quality problems were understood before developing the model. In order to maintain the life of data and the validity of analysis, rows with missing values in key attributes were deleted. The key characteristics, which were necessary in seismic analysis, were:

- time
- latitude
- longitude
- depth
- mag
- magType

These variables define the temporal occurrence, spatial location, and physical characteristics of each earthquake event. Records missing any of these attributes were excluded from further processing.

**Dataset summary after cleaning:**

- Original rows: 28,872
- Cleaned rows: 28,872
- Rows removed: 0
- Remaining missing values: 0

According to the fact that no records were deleted, it was possible to obtain all the necessary seismic characteristics in the data. The identification of all numerical columns was according to their type of data. Median imputation was used in the case of any numerical feature with missing values. The median was chosen over the mean as it is more resistance to outliers and skewed distribution, which prevails in seismic data (e.g., depth and magnitude). This method is statistically stable and has minimum distortion of the underlying data distribution. Categorical (object-type) attributes were distinguished and looked into missing entries. In case of the existence of missing values, they were filled with the mode (most recurring value) of the corresponding column. Mode imputation does not add any artificial categories, and its categorical consistency is preserved, and the most representative category is retained. The columns time and updated were expressed in a reasonable date format. It was this change that made possible the correct chronological sorting, time indexing, and time analysis. The conversion of timestamps to date-time objects can also be useful in extracting time-related characteristics effectively, as well as enhancing the usability with time-series modeling systems. In order to measure seasonal, periodic, and temporal variation in earthquake events, a few time-derived features were obtained using the time column:

- year
- month
- day
- hour
- minute
- dayofweek
- dayofyear
- weekofyear

These artificial features add explicit time scales to the data, and the models are able to capture localized effects of the short term behavior of the mean, seasonal effects and periodic variations in seismic activity. Some temporal variables: Some of them, e.g., month and hour, are periodically distributed. As an example, December changes into January, 23:00 turns into 00:00. In order to keep this cyclic continuity as well as avoid the artificial discontinuities in numerical representation, transformations carried out on sine and cosine were used. Cyclical figures were produced (the following):

- month_sin
- month_cos
- hour_sin
- hour_cos

This method of encoding can guarantee that periodic relationships are mathematically conserved and enhance the sensitivity of models to periodic relationships. To retrieve structured geographic data that can be structured based on the textual description of earthquake locations, a custom parsing function was developed that extracts the structured geographic information out of the column known as the place, which mostly includes the textual description of the earthquake location. The functional feature produced two more properties:

- location_detail: Specific locality description.
- region: Broader geographic region.

Normalization of the text and cleaning were done to eliminate redundant terms like region and area, so as to have a standardized and consistent categorical value. An analysis of the sampled frequency of the extracted feature of the region was then used to investigate the pattern of spatial distribution and regional seismic concentration. The dataset was organized as a time-series in order to permit the use of the time-dependence model. Autocorrelation Function and Partial Autocorrelation Function statistical tools were added to analyse the serial and lag dependencies. These analyses present an understanding of time persistence, seasonal aspects, and suitable lag choice in predictive modeling models. All in all, the preprocessing pipeline enabled high data quality and improved feature representation, as well as prepared the data set to be accurately represented in a robust statistical and machine learning-based seismic modeling. The statistics given above, individually across (i) depth intervals and, separately in (ii) depth categories, are pooled together in a single and comprehensive table to formulate the bidirectional relationship between focal depth and magnitude of earthquakes. This is the same presentation that allows one to make a direct comparison of central tendency, dispersion, and sample density using the two stratification schemes. Figure 20 gives the updated visualization (Table 22).

**Table 22:** Consolidated statistics describing magnitude by depth range and depth by magnitude category.

Magnitude statistics by depth range					Depth statistics by magnitude category				
Depth range (km)	Count	Mean mag	Median	Std dev	Mag category	Count	Mean depth	Median	Std dev
0–10	13,294	3.983	4.300	0.887	2–3	7561	23.06	11.00	30.85
10–30	4458	3.367	3.000	0.870	3–4	4705	44.93	18.00	80.36
30–70	5227	3.941	4.200	0.851	4–5	14,955	79.79	11.85	135.83
70–150	2850	4.041	4.300	0.737	5–6	1528	44.11	10.00	93.45
150–300	1394	4.238	4.300	0.553	>6	123	66.85	18.00	124.91
300–700	1286	4.365	4.300	0.356					

Notes: The left block summarizes how magnitude varies with increasing focal depth intervals, while the right block describes how depth distribution shifts across magnitude classes. Standard deviation quantifies dispersion within each subgroup

All the descriptive statistics are summarized into one overall table in order to provide a complete quantitative description of the global seismic dataset behind the spatial visualizations in Fig. 21. This table distills the amounts of data, range of magnitude, and varied focus depths such that World views on regional analysis and lumping drain into construct limits (Table 23).

**Table 23:** Consolidated global seismic activity summary statistics.

Metric	Value
Total earthquakes	28,872
Magnitude range	2.5–8.2
Depth range (km)	− 3.4–669.5

Notes: The dataset includes globally distributed seismic events with magnitudes ranging from moderate to major earthquakes and focal depths spanning shallow crustal to deep subduction-zone events

To better understand the relationships among the primary numerical variables in the seismic dataset, a correlation analysis was performed prior to model development. Correlation matrices are widely used in exploratory data analysis to reveal linear dependencies between variables and to detect potential multicollinearity that may affect model stability and interpretability. Figure 22 illustrates the pairwise Pearson correlation coefficients among the key numerical attributes, including earthquake magnitude, depth, signal characteristics, and measurement error indicators. The color intensity and numerical annotations represent the strength and direction of the correlations, where positive values indicate direct relationships and negative values indicate inverse relationships. Such visualization enables rapid identification of strongly related variables, which may influence feature selection, model design, and interpretation of seismic behavior. In particular, the matrix highlights how seismic signal properties (e.g., RMS and horizontal error) exhibit stronger associations with magnitude compared to other variables, while error-related attributes generally display weaker correlations with the target variable.

## Appendix B: Empirical results

### Baseline model comparison

In this section, the empirical assessment of five state-of-the-art deep learning models deployed in time-series seismic prediction is introduced. Baseline comparison is used in order to determine the initial raw predictive accuracy of each architecture before any kind of hyperparameter optimization/hybrid improvement. The reviewed models are N-HITS, N-BEATS, DeepAR, ConvLSTM, and Reformer. Various complementary statistical measures were used to evaluate performance; thus, a complete evaluation was done. These measures are Mean Squared Error (MSE), Root Mean Squared Error (RMSE), Mean Absolute Error (MAE), Relative Root Mean Squared Error (RRMSE), Mean Bias Error (MBE), Mean Absolute Percentage Error (MAPE), Symmetric Mean Absolute Percentage Error (SMAPE), Coefficient of Determination (R2), and NashSutcliffe Efficiency (NSE). Smaller values of MSE, RMSE, MAE, RRMSE, MAPE and SMAPE reflect stronger predictive ability and larger values of R2 and NSE (greater than one) to higher values of goodness-of-fit and explanatory power. Table 24 provides the direct results of the raw performance measures of all five models. Based on Table 24 N-HITS model has been observed to show the best baseline performance in almost all the evaluation metrics. It shows the lowest MSE (0.00385), RMSE (0.0557), MAE (0.0435), and RRMSE (0.176), which shows small predictive error and low error variance. It also achieves the largest values of R2 (0.889) and NSE (0.885), indicating a good ability to explain and model efficiency. N-BEATS is second overall, with its accuracy being competitive, with a little higher error values than the N-HITS. DeepAR and ConvLSTM have a moderate performance with the lowest error metrics and the highest Godf-of-fit scores among the tested baselines; the Reformer model shows the worst results. In general, current empirical comparison shows that N-HITS is the strongest architecture in the minimal strain conditions, with a solid base of further optimization and hybrid modeling improvements explained in the following parts.

**Table 24:** Baseline performance comparison of deep learning models.

Model	MSE	RMSE	MAE	RRMSE	MBE	MAPE (%)	SMAPE (%)	$\hbox {R}^{2}$	NSE
N-HITS	0.00385	0.0557	0.0435	0.176	0.0051	4.82	5.01	0.889	0.885
N-BEATS	0.00462	0.0620	0.0478	0.192	0.0047	5.36	5.58	0.872	0.868
DeepAR	0.00495	0.0642	0.0496	0.198	0.0059	5.61	5.84	0.864	0.860
ConvLSTM	0.00548	0.0682	0.0524	0.205	-0.0068	6.02	6.21	0.853	0.848
Reformer	0.00573	0.0700	0.0541	0.211	0.0043	6.18	6.39	0.846	0.842

A visualization structure inspired by a response surface methodology approach (RSM) is used to analyze error-based and goodness-of-fit information simultaneously to give a multidimensional view of model performance. Instead of interpreting every performance measure separately, Fig. 23 plots contour-like scatter plots that can simultaneously encode relationships among the primary error measures (MSE, MAE, RMSE), the relative error measures (MAPE, SMAPE, RRMSE), the bias (MBE) and the measures of the explanatory power indicators (R2, NSE). This unified visualization allows a systematic evaluation of trade-offs between accuracy, robustness and bias of competing forecasting architectures. The projection of complementary metrics on common axes and the color gradient presentation of other dimensions in the figure both reveal nonlinear interactions of performance and clustering behaviour that are not easily seen in tabular summaries. This multi-criteria approach to evaluation is especially significant in seismic time-series forecasting, where performance on one measure can approach deterioration on another, and an optimal way to select a model is to balance between them.

To further investigate the behavior of comparative models in a variety of evaluation criteria, we adopt a polar area visualization that concurrently represents relative performance variations in a small geometric structure. Each subplot corresponds to a specific metric, MSE, RMSE, MAE, MAPE, SMAPE, and RRMSE, and radial extensions are used to encode normalized performance scores of the competing forecasting architectures, as shown in Fig. 24. This polar representation allows the intuitive evaluation of dominance patterns, metric stability and cross-metric consistency, allowing the multi-objective comparison without having to examine the tabular representation alone. Using the performance magnitudes, plotted on the angular sectors, the result figure illustrates proportional performance gain and loss in all the models, which indicates the difference in absolute performance and the relative strength patterns. This type of visualization is especially applicable to the multi-metric seismic forecasting evaluation, where balanced performance over both the error and percentage-based indicators is required to be reliable to deploy.

### Feature selection results

The selection of features is an important part of the work that improves predictive performance by removing redundant, irrelevant, and noisy features and maintaining the most informative variables. In time-series modeling In time-series modeling In seismic time-series modeling, overfitting may be brought by high-dimensional input spaces, computational complexity may be increased, and generalization may be weakened. Thus, metaheuristic optimization methods were used to find a smaller and higher-quality feature set that is predictive and reduces the dimensionality. This paper was used to test ten optimization algorithms in terms of performance in feature selection: bGLO, bPSO, bWOA, bGA, bBA, bBBO, bDE, bSFS, bAPO, bMVO. Those algorithms were repeated several times to see that they have the statistical strength, and their performance was evaluated by a number of complementary measures. The evaluation standards that were taken into account are as follows:

- **Average Error**: Mean prediction error achieved using the selected feature subset. Lower values indicate better predictive accuracy.
- **Average Selected Size**: Average proportion of features selected relative to the total number of features. Smaller values reflect stronger dimensionality reduction.
- **Average Fitness**: Mean fitness value across independent runs, reflecting the trade-off between error minimization and subset compactness.
- **Best Fitness**: Best (minimum) fitness value achieved among all runs.
- **Worst Fitness**: Worst (maximum) fitness value observed, indicating performance stability boundaries.
- **Standard Deviation of Fitness**: Measures the consistency and robustness of the algorithm across runs. Lower values indicate higher stability.

Table 25 presents the comparative results of the evaluated feature selection algorithms.

**Table 25:** Comparative performance of feature selection algorithms.

Metric	bGLO	bPSO	bWOA	bGA	bBA	bBBO	bDE	bSFS	bAPO	bMVO
Average Error	0.3923	0.5271	0.5354	0.5289	0.5012	0.5079	0.5112	0.4175	0.4521	0.4478
Average Selected Size	0.3521	0.6170	0.7375	0.7582	0.7586	0.7004	0.5673	0.5336	0.6488	0.6367
Average Fitness	0.4675	0.5722	0.5920	0.5790	0.5772	0.5979	0.5835	0.4902	0.4973	0.5342
Best Fitness	0.3627	0.5352	0.4767	0.5280	0.5483	0.5133	0.4799	0.4013	0.4372	0.4496
Worst Fitness	0.4478	0.5937	0.5645	0.5937	0.6230	0.6152	0.5794	0.4591	0.5322	0.5515
Standard Deviation Fitness	0.2940	0.3896	0.3982	0.3915	0.4284	0.4334	0.3915	0.3067	0.3224	0.3697

From the results, bGLO achieves the lowest Average Error (0.3923) and the smallest Average Selected Size (0.3521), indicating superior predictive accuracy with significant dimensionality reduction. Additionally, it records the best Average Fitness (0.4675) and Best Fitness (0.3627), demonstrating strong optimization capability and consistent convergence behavior. The relatively low standard deviation further confirms its robustness across multiple runs. Overall, the comparative analysis demonstrates that bGLO provides the most balanced trade-off between minimizing prediction error and reducing feature dimensionality, making it the most effective feature selection approach among the evaluated algorithms.

To provide a comprehensive comparative evaluation of the optimization algorithms, we present an aggregated multi-metric performance analysis that simultaneously captures accuracy, convergence behavior, robustness, and variability characteristics. As illustrated in Fig. 25, each algorithm is evaluated across six complementary indicators: average error, average selected size, average fitness, best fitness, worst fitness, and standard deviation of fitness. This unified visualization enables holistic assessment of algorithmic stability and solution quality rather than relying on a single performance criterion. By juxtaposing central tendency measures (average and best fitness) with dispersion-based indicators (worst fitness and standard deviation), the figure highlights trade-offs between exploitation strength and population diversity. Additionally, the inclusion of the best-observed value marker facilitates rapid identification of dominant configurations across metrics. Such multi-dimensional comparison is particularly important in metaheuristic optimization, where performance consistency and robustness are as critical as absolute solution quality.

In order to offer a brief but detailed summary of the performance of the optimization in all the evaluation criteria, we are presenting a comparative analysis in terms of a heatmap. Figure 26 demonstrates that a row indicates a specific performance measure, such as average error, average selected size, average fitness, best fitness, worst fitness, and standard deviation of fitness, and each column corresponds to a specific candidate optimization algorithm. The magnitude of each metric is coded in the color, which allows instantly identifying its dominance in the visual representation of the algorithms, stability patterns, and trade-offs among the convergence quality and variability. A visualization as a matrix, unlike isolated bar charts or tabular summaries, allows simultaneous five-metric and cross- algorithm comparison and shows consistency, robustness, as well as relative superiority within the same integrated framework. This kind of representation is especially useful in multi-objective optimization problems, where a trade-off in terms of accuracy and dispersion is used to define usability in terms of the practicality of the algorithm.

### Deep learning performance after feature selection

The identified best feature subsets based on the metaheuristic algorithm were fed to the deep learning systems after their identification. The purpose of this step is to assess the effect that dimensionality reduction may have on predictive accuracy, model generalization and error stability. The feature selection, aimed at removing the redundant and irrelevant attributes, is likely to reduce the noise, improve the convergence behavior, and increase the precision of the forecasts. In order to provide a high rigor point of comparison with the baseline results, the same assessment measures were retained: Mean Squared Error (MSE), Root Mean Squared Error (RMSE), Mean Absolute Error (MAE), Relative root mean squared error (RRMSE), Coefficient of Determination (R2), and Nash-Sutcliffe Efficiency (NSE). Reduced error measurements and increased values of R2 and NSE suggest enhanced predictive power.

The performance of the five deep learning models following the feature selection is provided in Table 26.

**Table 26:** Deep learning performance after feature selection.

Model	MSE	RMSE	MAE	RRMSE	MBE	MAPE (%)	SMAPE (%)	$\hbox {R}^{2}$	NSE
N-HITS	0.00215	0.0464	0.0348	0.141	0.0021	3.21	3.36	0.945	0.942
N-BEATS	0.00274	0.0523	0.0386	0.158	0.0018	3.74	3.88	0.931	0.928
DeepAR	0.00298	0.0546	0.0401	0.164	0.0026	3.95	4.07	0.924	0.920
ConvLSTM	0.00335	0.0578	0.0427	0.171	− 0.0039	4.28	4.39	0.914	0.909
Reformer	0.00352	0.0593	0.0439	0.175	0.0015	4.41	4.55	0.907	0.902

The findings show a significant change in all the models as compared to the baseline situation. N-HITS has the highest overall performance, the lowest MSE (0.00215), RMSE (0.0464), and MAE (0.0348), and the highest values of $R^2$ (0.945) and NSE (0.942). These results indicate that feature selection has a great deal in boosting predictive accuracy and model explanatory power. Likewise, there are significant memory loss rates in N-BEATS and DeepAR, as well as refinement of indices of goodness of fit. Despite the fact that ConvLSTM and Reformer lag a notch lower in terms of relative ranking, both models demonstrate the presence of performance improvement over their baseline settings. Throughout, the subsumption of an optimized set of features causes steady accuracy of forecasting results, which validates the usefulness of the feature selection step in optimization of deep learning seismic time-series predictions.

After applying the feature selection structure, a devoted study of the predictive accuracy was made with core measures of regression errors. Figure 27 provides a comparative study of Mean Squared Error (MSE), Root Mean Squared Error (RMSE), and Mean Absolute Error (MAE) among the competing deep learning architectures. Since these measures are a direct measure of the remnants of prediction, they give a straightforward measure of a precise model following dimensional reduction. The lower values are associated with a better performance of generalization and a lower range of variance of predicted seismic magnitudes. The visualization also elucidates the most effective configuration in every measure with explicit indicators, which allows one to quickly retrieve the most precise model within the compressed feature space. Post-selection assessment is essential in this case to ensure that the reduction of features would not impair the predictive power without taking a toll on the accuracy or stability.

Besides absolute measures of error, percentage-based measures give a scale-free appraisal of forecasting accuracy, thus allowing reasonable comparison across different levels of magnitude. Figure 28 compares the performance of the competing models under Mean Absolute Percentage Error (MAPE) and Symmetric Mean Absolute Percentage Error (SMAPE) as the lollipop charts. The visualization focuses on the differences in predictive deviations with respect to one another without compromising the fact that metrics are ranked higher or lower. The horizontal reference points out the best performance when it is observed that the best architecture can be quickly found when it comes to proportional error criteria. Since predicting seismic magnitudes entails nonhomogeneous distributions of amplitudes, percentage indicators tend to be especially informative in the context of measuring the sensitivity to scale variations and providing equalized generalization ability over low- and high-intensity events.

### Optimized N-HITS

In order to obtain additional predictive accuracy, an N-HITS architecture was combined with a number of metaheuristic optimization algorithms to tune the hyperparameters. Although the baseline and feature-selected models were well-performing, hyperparameter optimization would be effective to utilize the representational ability of the deep hierarchical models to the full extent. Convergence behavior tends to be better when the model is properly tuned, resulting in the generalization error being lower and the predictions of the model being more consistent on many repeat runs. In the research, the N-HITS model parameters, GLO, PSO, DE, GA, WAO, BA, BBO, SFS, APO, and MVO, were optimized using 10 optimization processes. The optimizers were run many times using different independent executions to provide statistical reliability. The results reported have been in the form of mean error, standard deviation, and this gives a picture of the predictive accuracy and the stability of the algorithm.

Evaluation metrics were kept constant: Mean Squared Error (MSE), Root Mean Squared Error (RMSE), Mean Absolute Error (MAE), Relative Root Mean Squared Error (RRMSE), Mean Bias Error (MBE), Mean Absolute Percentage Error (MAPE), Symmetric Mean Absolute Percentage Error (SMAPE), Coefficient of Determination(R2), NashSutcliffe Efficiency (NSE). The reduced error values and increased values in R2 and NSE mean that the model performs better. In Table 27 we saw a summary of the performance of the optimized N-HITS model with various optimization strategies.

**Table 27:** Performance of optimized N-HITS using different metaheuristic algorithms.

Optimizer	MSE	RMSE	MAE	RRMSE	MBE	MAPE (%)	SMAPE (%)	$\hbox {R}^{2}$	NSE
GLO+N-HITS	$4.200\textrm{e}{-04} \pm 4.200\textrm{e}{-06}$	$0.0205 \pm 0.0001$	$0.0162 \pm 0.0002$	$0.0640 \pm 0.0006$	$4.000\textrm{e}{-04} \pm 4.800\textrm{e}{-06}$	$0.850 \pm 0.004$	$0.920 \pm 0.011$	$0.9830 \pm 0.0039$	$0.9810 \pm 0.0049$
PSO+N-HITS	$1.280\textrm{e}{-03} \pm 1.365\textrm{e}{-05}$	$0.0358 \pm 0.0002$	$0.0282 \pm 0.0003$	$0.1117 \pm 0.0012$	$1.100\textrm{e}{-03} \pm 1.442\textrm{e}{-05}$	$1.550 \pm 0.012$	$1.650 \pm 0.022$	$0.9770 \pm 0.0046$	$0.9750 \pm 0.0054$
DE+N-HITS	$1.300\textrm{e}{-03} \pm 1.473\textrm{e}{-05}$	$0.0361 \pm 0.0002$	$0.0283 \pm 0.0003$	$0.1126 \pm 0.0013$	$1.100\textrm{e}{-03} \pm 1.564\textrm{e}{-05}$	$1.580 \pm 0.016$	$1.680 \pm 0.025$	$0.9760 \pm 0.0052$	$0.9740 \pm 0.0060$
GA+N-HITS	$1.320\textrm{e}{-03} \pm 1.584\textrm{e}{-05}$	$0.0363 \pm 0.0002$	$0.0285 \pm 0.0004$	$0.1135 \pm 0.0014$	$1.100\textrm{e}{-03} \pm 1.687\textrm{e}{-05}$	$1.600 \pm 0.020$	$1.700 \pm 0.028$	$0.9750 \pm 0.0059$	$0.9730 \pm 0.0065$
WAO+N-HITS	$1.380\textrm{e}{-03} \pm 1.748\textrm{e}{-05}$	$0.0371 \pm 0.0002$	$0.0290 \pm 0.0004$	$0.1160 \pm 0.0015$	$1.200\textrm{e}{-03} \pm 1.973\textrm{e}{-05}$	$1.650 \pm 0.025$	$1.750 \pm 0.031$	$0.9740 \pm 0.0065$	$0.9720 \pm 0.0070$
BA+N-HITS	$1.400\textrm{e}{-03} \pm 1.867\textrm{e}{-05}$	$0.0374 \pm 0.0002$	$0.0291 \pm 0.0004$	$0.1168 \pm 0.0016$	$1.200\textrm{e}{-03} \pm 2.107\textrm{e}{-05}$	$1.680 \pm 0.030$	$1.780 \pm 0.034$	$0.9730 \pm 0.0071$	$0.9710 \pm 0.0076$
BBO+N-HITS	$1.430\textrm{e}{-03} \pm 2.002\textrm{e}{-05}$	$0.0378 \pm 0.0003$	$0.0293 \pm 0.0004$	$0.1181 \pm 0.0017$	$1.300\textrm{e}{-03} \pm 2.427\textrm{e}{-05}$	$1.720 \pm 0.035$	$1.820 \pm 0.038$	$0.9720 \pm 0.0078$	$0.9700 \pm 0.0081$
SFS+N-HITS	$1.450\textrm{e}{-03} \pm 2.127\textrm{e}{-05}$	$0.0381 \pm 0.0003$	$0.0294 \pm 0.0005$	$0.1189 \pm 0.0017$	$1.300\textrm{e}{-03} \pm 2.571\textrm{e}{-05}$	$1.750 \pm 0.040$	$1.850 \pm 0.041$	$0.9710 \pm 0.0084$	$0.9690 \pm 0.0086$
APO+N-HITS	$1.470\textrm{e}{-03} \pm 2.254\textrm{e}{-05}$	$0.0383 \pm 0.0003$	$0.0295 \pm 0.0005$	$0.1197 \pm 0.0018$	$1.300\textrm{e}{-03} \pm 2.716\textrm{e}{-05}$	$1.770 \pm 0.045$	$1.870 \pm 0.044$	$0.9700 \pm 0.0091$	$0.9680 \pm 0.0091$
MVO+N-HITS	$1.500\textrm{e}{-03} \pm 2.400\textrm{e}{-05}$	$0.0387 \pm 0.0003$	$0.0297 \pm 0.0005$	$0.1209 \pm 0.0019$	$1.400\textrm{e}{-03} \pm 3.080\textrm{e}{-05}$	$1.800 \pm 0.050$	$1.900 \pm 0.048$	$0.9690 \pm 0.0097$	$0.9670 \pm 0.0097$

The findings make it clear that GLO+N-HITS is the most successful with regards to all measures of evaluation since it has significantly smaller error values and greater goodness of fit strengths than other optimization strategies. Furthermore, low standard deviation proves that it is highly stable and converges with a consistent behavior. These observations confirm the usefulness of the suggested optimization plan to increase significantly the predictability of the N-HITS architecture significantly.

The strip-plot analysis with uncertainty quantification used to evaluate both central performance tendencies as well as stability properties of the optimization algorithms is provided. As shown in Fig. 29, the subplot itself is reporting the mean of a particular metric with its standard deviation ($\pm 1\sigma$). The measured measures are the absolute error measures (MSE, RMSE, MAE), the measures in percentage (MAPE, SMAPE, RRMSE), bias (MBE) and goodness-of-fit measures (NSE, R2). The marked reference markers indicate the most effective performance in terms of each metric, allowing to quickly identify the most competitive optimizer and, at the same time, displaying the dispersion of variability through error bars. Such duality of representation especially matters in stochastic metaheuristic optimization, where convergence consistency and reliability of the algorithm become as significant as the mean performance.

In order to concurrently analyze predictive performance and algorithmic stability, we employ a holistic performance-stability analysis on complementary statistical summaries. As Fig. 30 shows, the left panel shows the average value of all evaluation indicators across optimization algorithms, which makes a direct comparison of central performance trends. Reported in the right panel is the coefficient of variation (CV%), which measures the relative dispersion, and a smaller coefficient of variation implies that there is more stable and reliable behavior of the algorithm with a series of independent runs. This dual-heatmap framework can be used to strike a balance in the interpretation of the accuracy–stability trade-offs by combining both the mean performance and variability measures. This is of crucial importance as such analysis, when applied to stochastic metaheuristic optimization, significantly forces high-quality performance in average performance to be highly reliable and repeatable in convergence.
